# Superparamagnetic nanoparticle-catalyzed coupling of 2-amino pyridines/pyrimidines with *trans*-chalcones†

**DOI:** 10.1039/c9ra00097f

**Published:** 2019-02-13

**Authors:** Oanh T. K. Nguyen, Pha T. Ha, Ha V. Dang, Yen H. Vo, Tung T. Nguyen, Nhan T. H. Le, Nam T. S. Phan

**Affiliations:** Faculty of Chemical Engineering, HCMC University of Technology, VNU-HCM 268 Ly Thuong Kiet, District 10 Ho Chi Minh City Vietnam lthnhan@hcmut.edu.vn ptsnam@hcmut.edu.vn

## Abstract

An aerobic coupling of 2-aminopyrimidines or 2-aminopyridines with *trans*-chalcones to afford aroylimidazo[1,2-*a*]pyrimidines and aroylimidazo[1,2-*a*]pyridines is reported. Reactions proceed in the presence of CuFe_2_O_4_ superparamagnetic nanoparticle catalyst, two equivalents of iodine, oxygen oxidant, and 1,4-dioxane solvent. The catalyst is superior to many common copper or iron complexes. Copper ferrite could be easily separated by magnetic decantation and reused up to 5 times without a major loss of activity. The method described here marks a rare example of using a simple, heterogeneous catalyst for synthesis of fused heterocycles. To our best knowledge, aroylimidazo[1,2-*a*]pyrimidines and aroylimidazo[1,2-*a*]pyridines were not previously synthesized using this protocol.

## Abstract 2

An aerobic coupling of 2-aminopyrimidines or 2-aminopyridines with *trans*-chalcones to afford aroylimidazo[1,2-*a*]pyrimidines and aroylimidazo[1,2-*a*]pyridines is reported.

## Graphical abstract

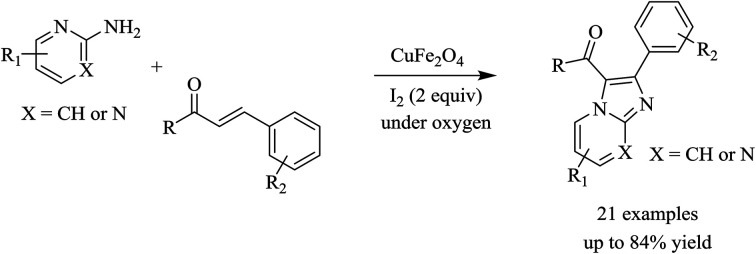

## 1. Introduction

Imidazo[1,2-*a*]pyridines find uses as carbene-typed ligands, organic materials, and in commercial drugs.^1^ Derivatization of imidazo[1,2-*a*]pyridines has recently emerged with regards to simple, efficient methods.^2^ A few prototypical derivatives such as aroylimidazo[1,2-*a*]pyridines are attracting attention of synthetic chemists due to potent bioactivities.^1^ Common methods are mostly focusing on coupling of α,β-unsaturated ketones or ketimines with 2-aminopyridines, since the starting materials are readily available or easily obtained.^3^ Notably, a few reports use prefunctionalized starting materials, thus limiting the practicality.^4^ Moreover, the use of strong bases, harsh conditions, ligand requirement, and nonreusable catalysts is notoriously challenging with regard to green chemistry. Thus, methods using simple reagents in combination with reusable catalysts for the synthesis of aroylimidazo[1,2-*a*]pyridines or aroylimidazo[1,2-*a*]pyrimidines still remain to be developed.

**Scheme 1:**
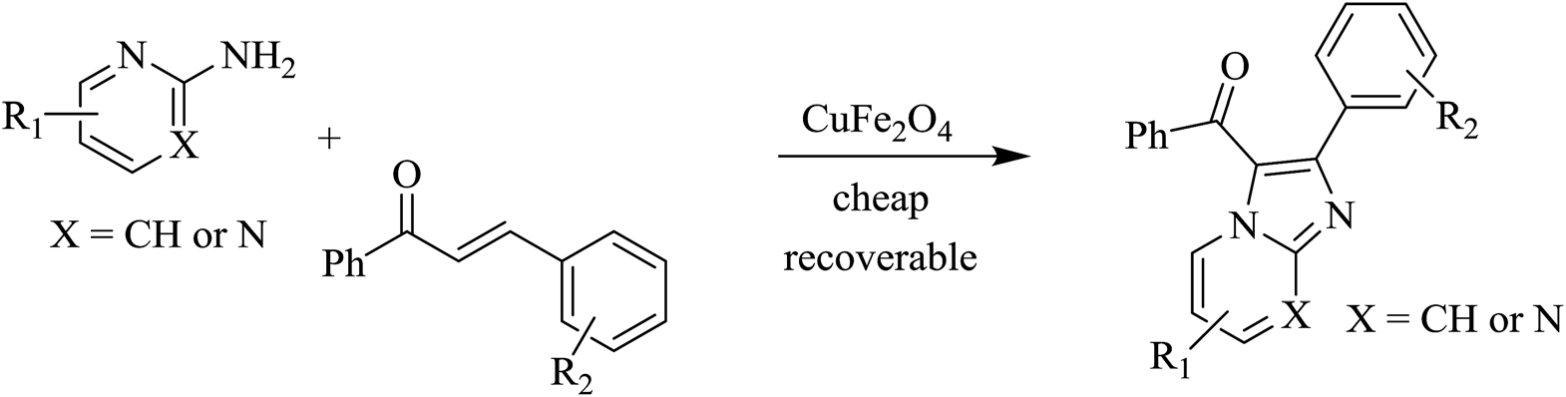
Heterogeneous copper-catalyzed coupling of 2-amino pyridines/pyrimidines with chalcones.

Nanocatalysis comprises methods of using nanoparticles to facilitate chemical transformations. Over the last two decades, significant efforts have led to the use of many nanoparticles in synthetic chemistry, bridging the gap between traditional heterogeneous and homogeneous catalysis.^5^ The catalytic activity of nanocatalysts is directly referred to surface areas of the particles; thus, most active sites would be readily accessible to reactants at nanometer dimension. Practicality of the nanoparticles, however, suffers from challenges of separation and recovery.^5*a*^ Using superparamagnetic particles in catalysis would be a prominent solution as the nanoparticles could be recovered by magnetic decantation instead of filtration or centrifugation. Consequently, loss of the catalyst would be thwarted. Many reports of using superparamagnetic catalysts for organic reactions are known.^6^ Due to uniquely high activity of copper catalysis for cross couplings, the use of magnetic copper-based catalysts is exponentially growing.^7^ A couple of seminal reports by Glorius and co-workers emphasize a prominent role of copper impregnated on magnetite (CuO/Fe_3_O_4_) catalyst for C–H arylation.^7^ As our continuous interest in leveraging catalytic activity of copper ferrite nanoparticles,^8^ we report here a method for CuFe_2_O_4_-catalyzed coupling of 2-amino pyridines/pyrimidines with *trans*-chalcones to synthesize aroylimidazo[1,2-*a*]pyridines/aroylimidazo[1,2-*a*]pyrimidines (Scheme 1). The conditions allow a mild and straightforward route to prepare fused N-heterocycles in the presence of a heterogeneous catalyst, which is still limited in the literature.

## 2. Experimental section

CuFe_2_O_4_ nanoparticles were purchased from Sigma-Aldrich and used as received. A typical reaction follows the general procedure: to a pressurized vial was charged *trans*-chalcone (62 mg, 0.3 mmol), 2-aminopyrimidine (48 mg, 0.5 mmol), I_2_ (254 mg, 0.6 mmol), CuFe_2_O_4_, diphenyl ether (51 mg, 0.3 mmol) internal standard, and 1,4-dioxane (2.5 mL). The vial was heated at 140 °C for 7 h under an oxygen atmosphere. The reaction progress was monitored by withdrawing an aliquot of the reaction mixture and quenching with Na_2_S_2_O_3_ solution (10% v/v, 1 mL). Organic compounds then were extracted with ethyl acetate (2 mL × 2). Combined organic phases were dried over Na_2_SO_4_, filtered, and analyzed by gas chromatography.

To test the reusability of the superparamagnetic nanoparticles, the copper-iron oxide CuFe_2_O_4_ (8 mg, 30 μmol) was added to a mixture of *trans*-chalcone (62 mg, 0.3 mmol), 2-aminopyrimidine (48 mg, 0.5 mmol), I_2_ (254 mg, 0.6 mmol), and 1,4-dioxane (2.5 mL). The mixture was heated at 140 °C for 7 h under an oxygen atmosphere. The solid catalyst was collected by magnetic decantation after reaction was complete. The recovered superparamagnetic CuFe_2_O_4_ nanoparticles were carefully washed with ethyl acetate (10 mL), ethanol (10 mL), and acetone (10 mL) to remove impurities. The resulting solids were then heated at 120 °C under strong vacuum for 6 h. The catalyst was then used for a new catalytic reaction using the procedure of the first run.

Yields presented in Table 2 are isolated yields. All of the entries were done using the following procedure unless notice: to a pressurized vial was charged chalcone (0.3 mmol), 2-aminopyrimidine or 2-aminopyrimidine (0.5 mmol), I_2_ (254 mg, 0.6 mmol), CuFe_2_O_4_ (30 μmol, 8 mg), and 1,4-dioxane (2.5 mL). In Scheme 3, the amount of reactants and reagents were used as: dibenzylideneacetone (0.4 mmol, 94 mg), 2-aminopyridine (0.8 mmol, 75 mg), CuFe_2_O_4_ (40 μmol, 10 mg), I_2_ (0.8 mmol, 203 mg), 1,4-dioxane (3 mL). The vial was heated at 140 °C for 7 h under an oxygen atmosphere. The reaction progress was monitored by withdrawing an aliquot of the reaction mixture and quenching with Na_2_S_2_O_3_ solution (10% v/v, 1 mL). Organic compounds then were extracted with ethyl acetate (2 mL × 2). Combined organic phases were dried over Na_2_SO_4_, filtered, and concentrated. Purification by column chromatography afforded desire products. Characterization of the compounds used ^1^H, ^13^C, and ^19^F spectra, which were recorded on Bruker AV 500, JEOL EC-400, or JEOL EC-600 spectrometers using residual solvent peaks as references.

**Table 1:** Optimization conditions

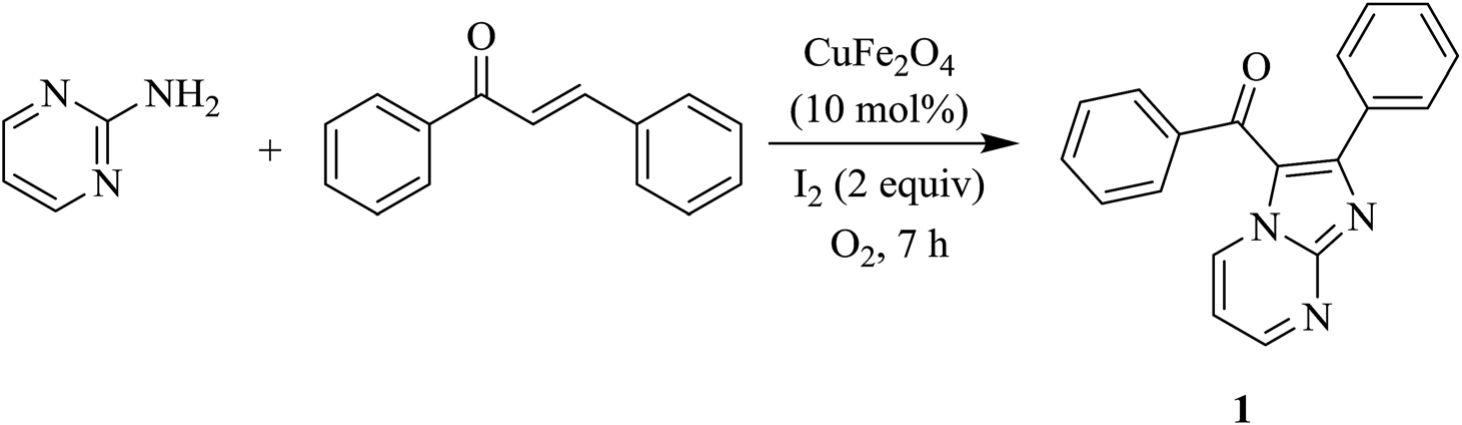					
Entry	Catalyst	Temperature (°C)	2-Aminopyrimidine (equiv.)	Solvent	Yield^a^, %
1	CuFe_2_O_4_	60	1.6	1,4-Dioxane	7
2	CuFe_2_O_4_	120	1.6	1,4-Dioxane	66
3	CuFe_2_O_4_	140	1.6	1,4-Dioxane	84, 81^b^
4	CuFe_2_O_4_	140	1	1,4-Dioxane	70
5	CuFe_2_O_4_	140	2.3	1,4-Dioxane	63
6	CuFe_2_O_4_	140	1.6	DMF	9
7	CuFe_2_O_4_	140	1.6	DMSO	23
8	CuFe_2_O_4_	140	1.6	Chlorobenzene	70
9^c^	CuFe_2_O_4_	140	1.6	1,4-Dioxane	0
10^d^	CuFe_2_O_4_	140	1.6	1,4-Dioxane	49
11^e^	CuFe_2_O_4_	140	1.6	1,4-Dioxane	15
12^f^	CuFe_2_O_4_	140	1.6	1,4-Dioxane	56
13	—	140	1.6	1,4-Dioxane	15
14^g^	CuFe_2_O_4_	140	1.6	1,4-Dioxane	24
15	CuI	140	1.6	1,4-Dioxane	38
16	CuBr_2_	140	1.6	1,4-Dioxane	58
17	Cu(OAc)_2_	140	1.6	1,4-Dioxane	34
18	FeCl_2_	140	1.6	1,4-Dioxane	67
19	FeCl_3_	140	1.6	1,4-Dioxane	41
20	Fe_2_O_3_^h^	140	1.6	1,4-Dioxane	21
21	CuO^h^	140	1.6	1,4-Dioxane	29

a *Trans*-Chalcone (0.3 mmol), CuFe_2_O_4_ (30 μmol, 10 mol%), I_2_ (0.6 mmol), solvent (2.5 mL), under O_2_, 7 h. Yields are GC yields using diphenyl ether internal standard.

b Isolated yield.

c No I_2_.

d I_2_ (0.45 mmol).

e I_2_ (0.3 mmol).

f CuFe_2_O_4_ (22.5 μmol, 7.5 mol%).

g Under argon.

h Nano oxides.

**Table 2:** Scope of aroylimidazo[1,2-*a*]pyrimidine/aroylimidazo[1,2-*a*]pyridine synthesis^a^

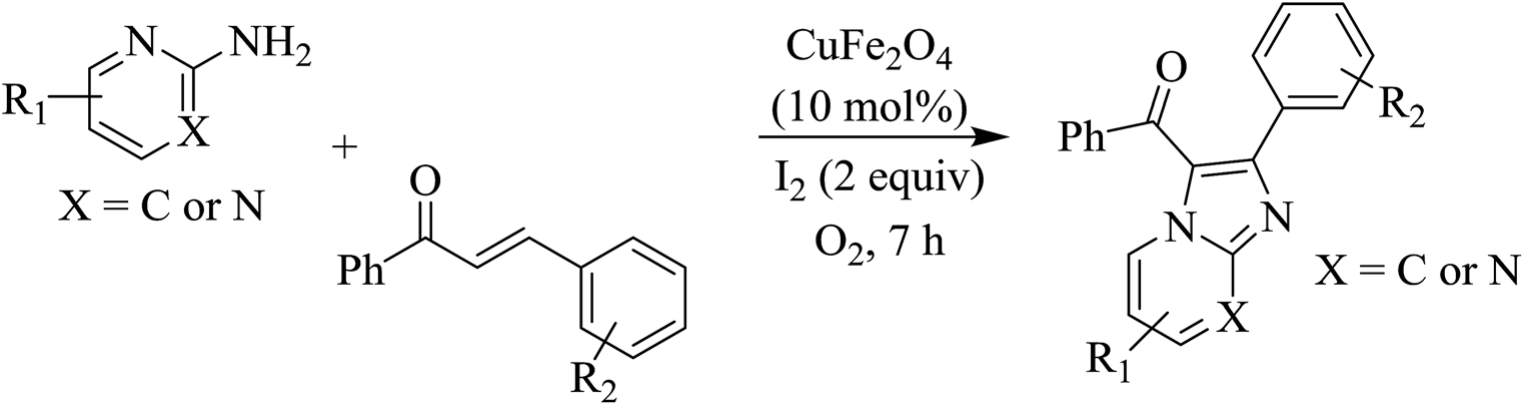				
Entry	Chalcones	2-Aminopyrimi-dines or 2-aminopyridines	Products	Yield^b^, %
1	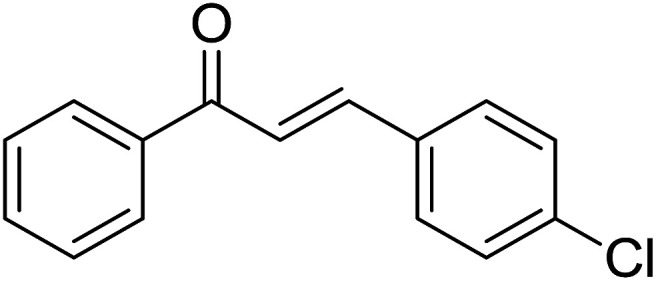	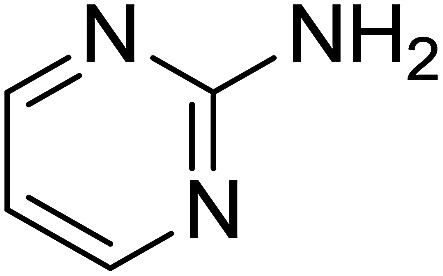	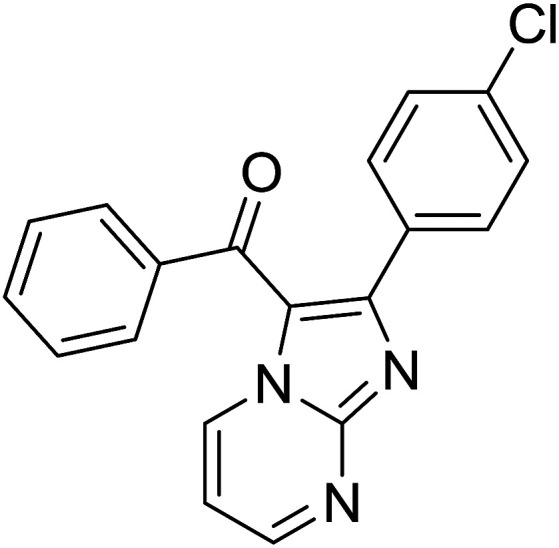	76
2	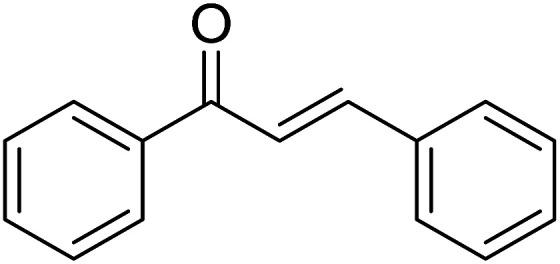	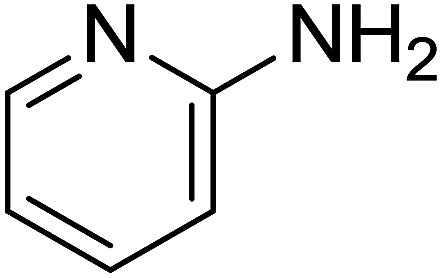	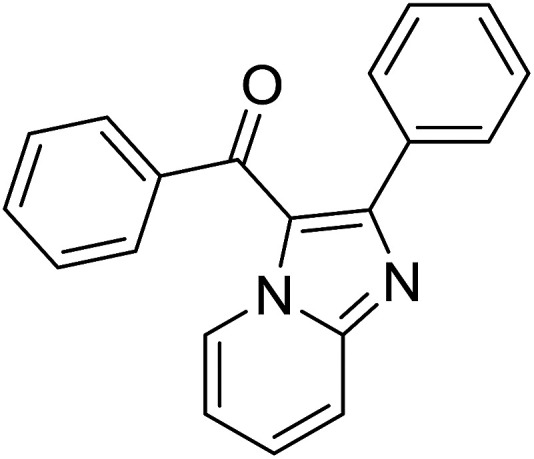	65
3	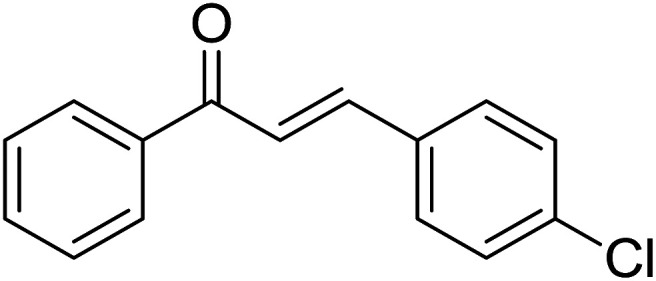	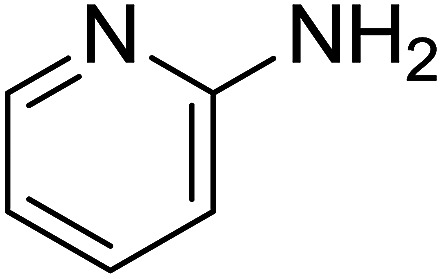	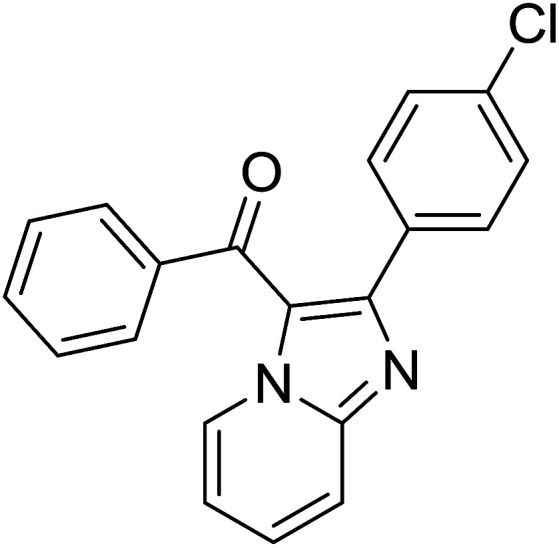	67
4	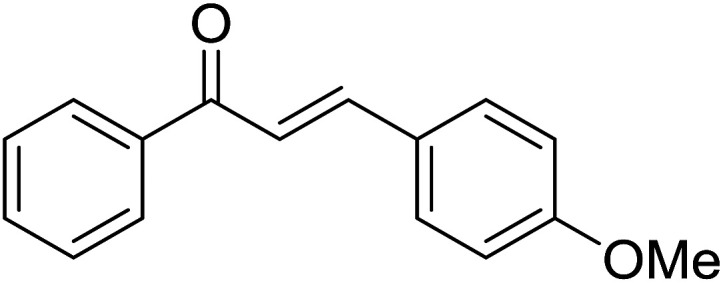	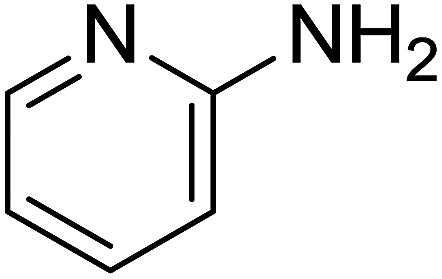	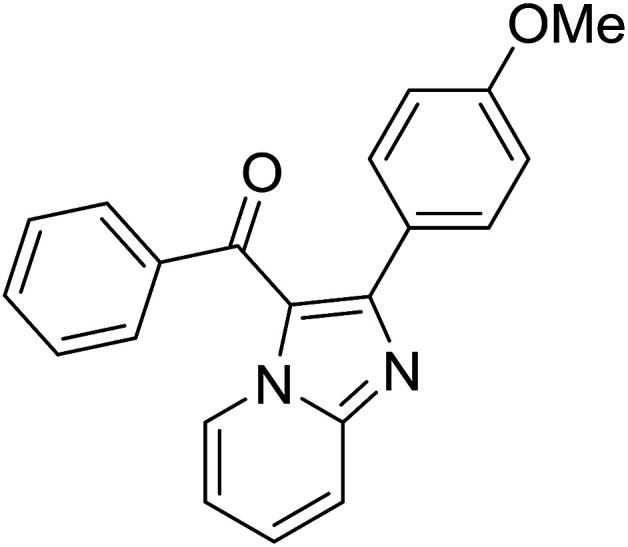	68
5	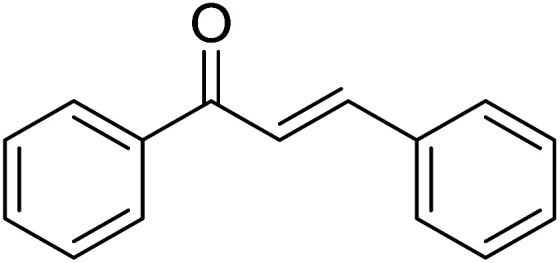	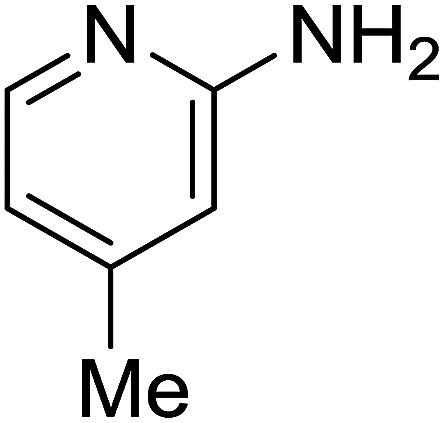	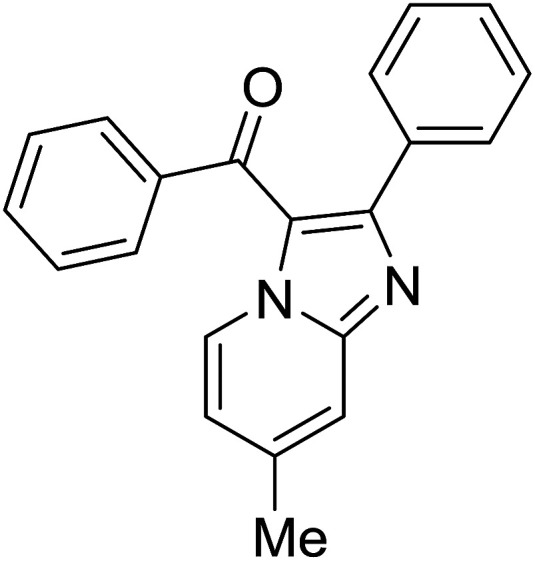	75
6	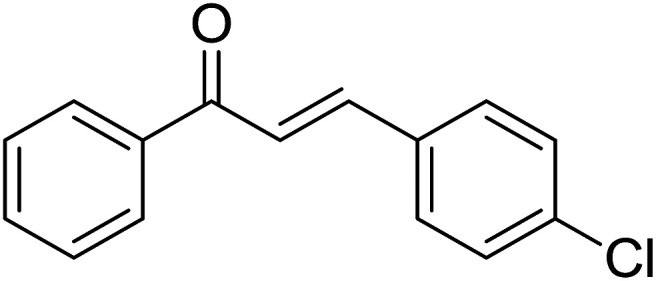	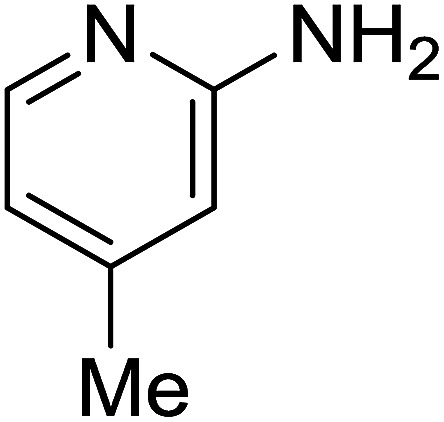	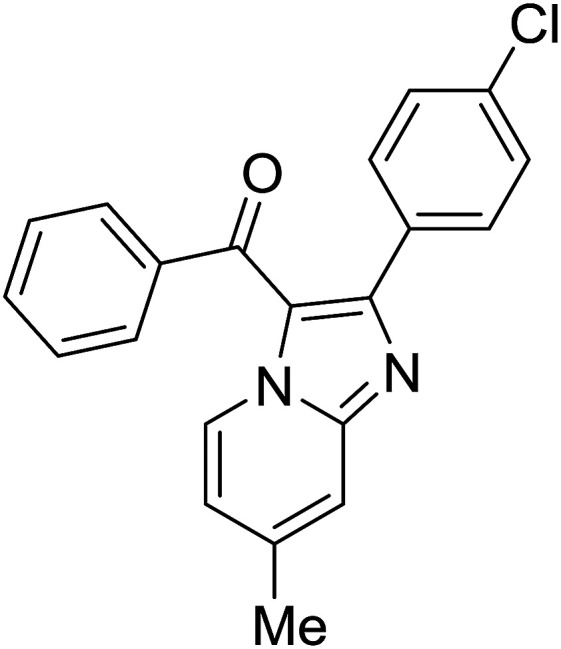	69
7	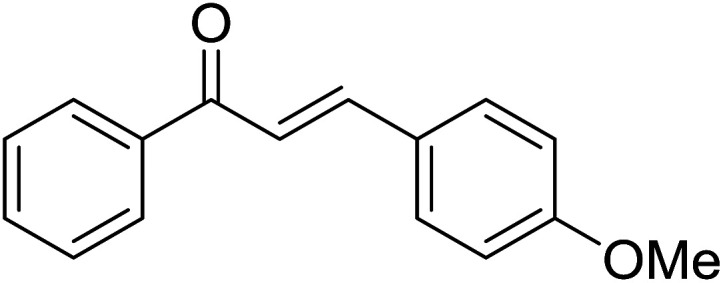	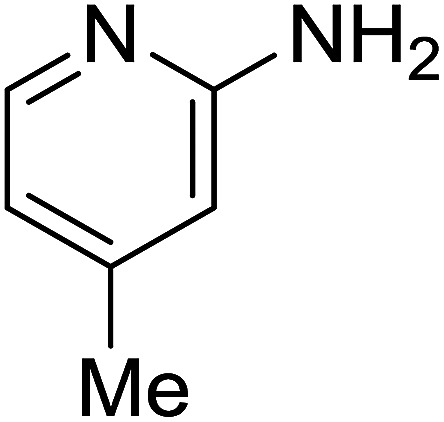	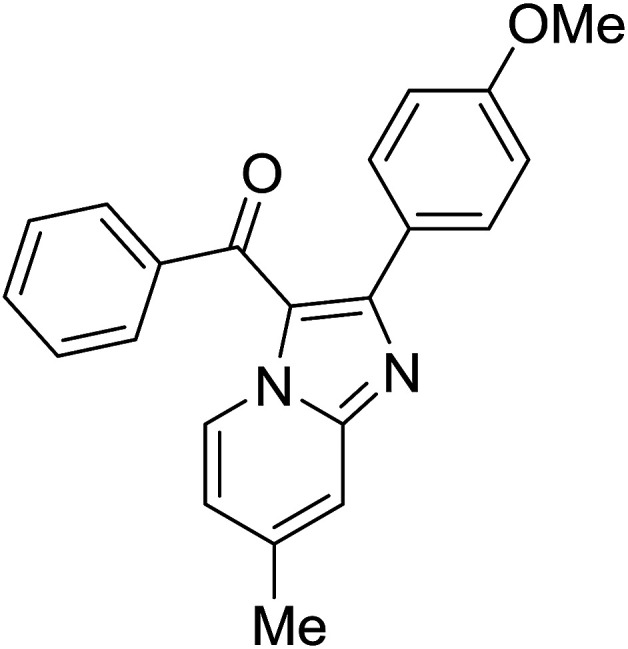	70
8	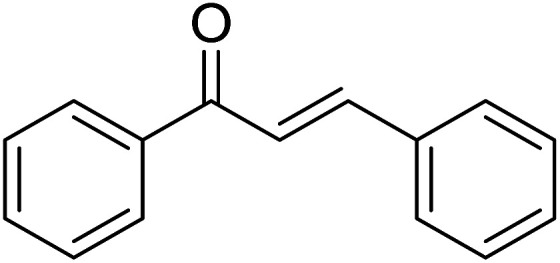	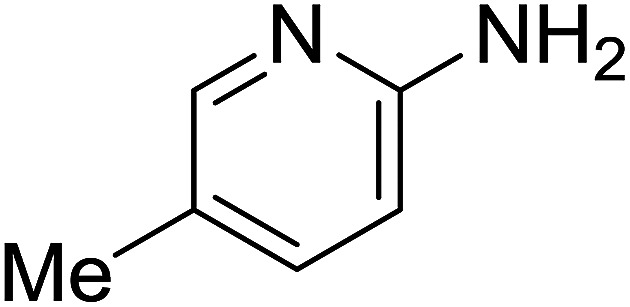	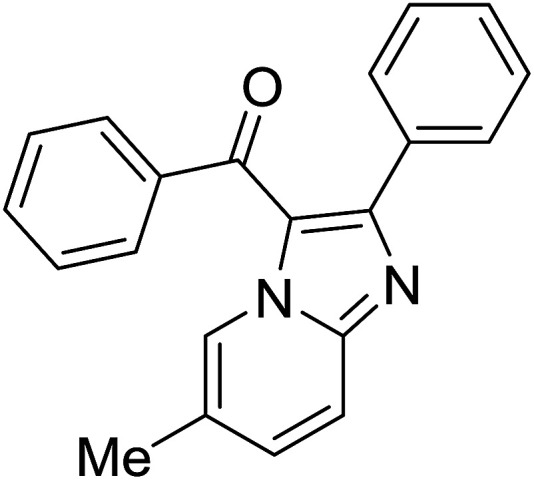	71
9	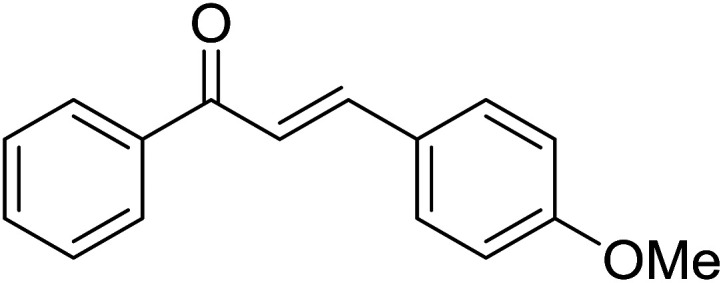	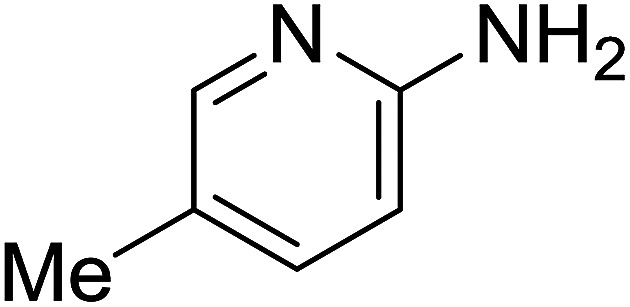	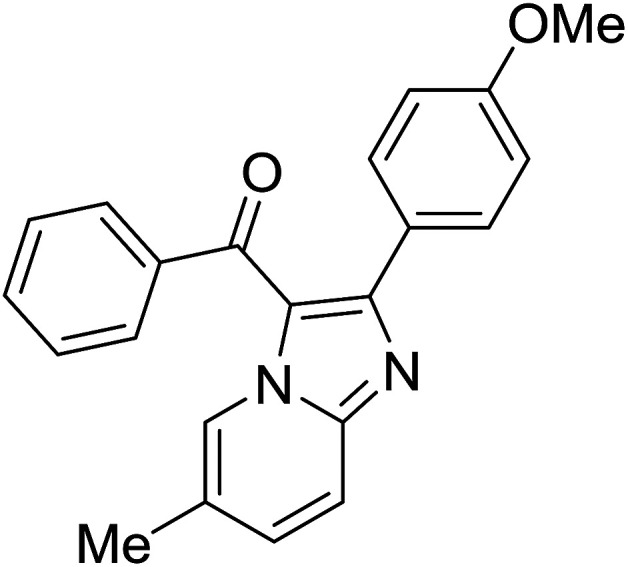	75
10	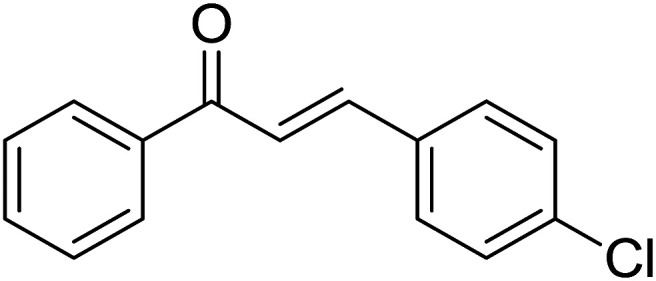	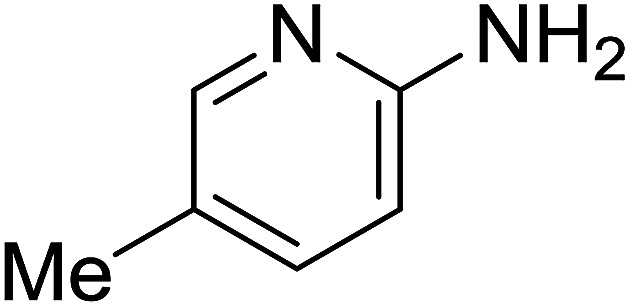	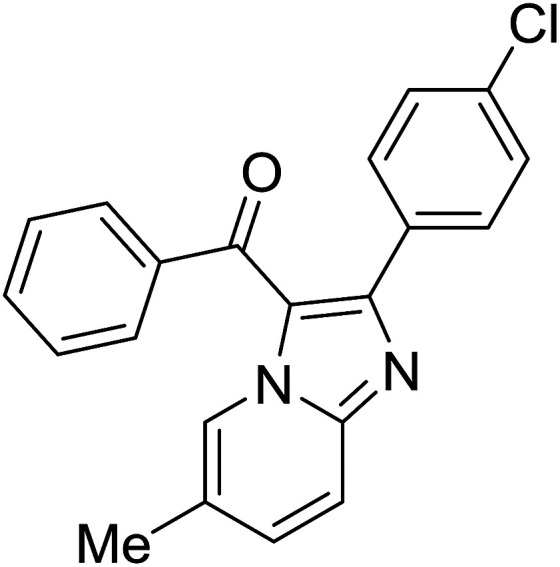	75
11	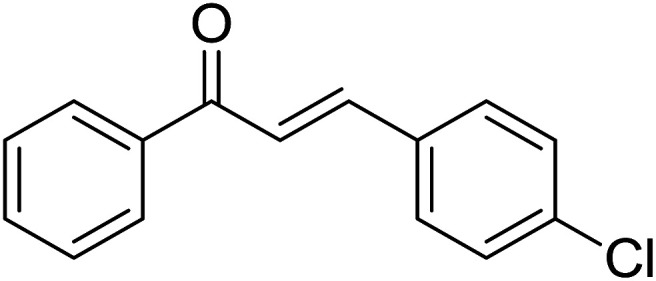	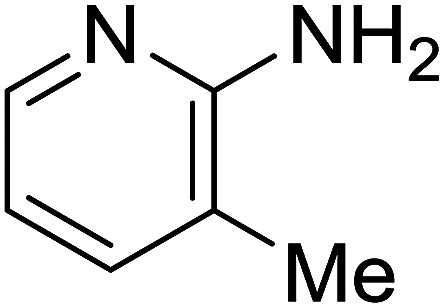	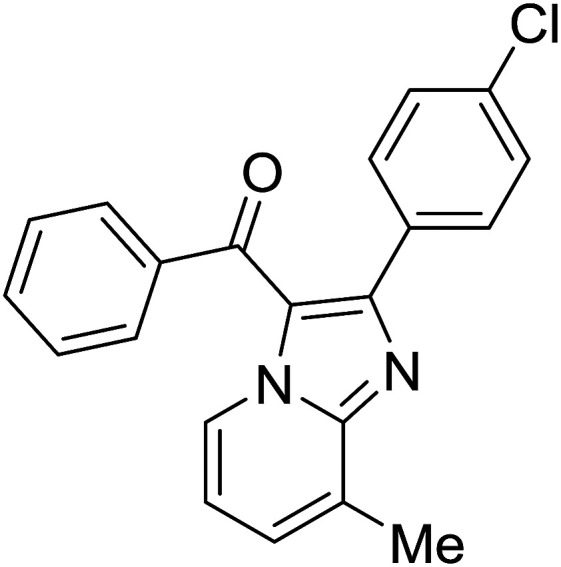	73
12	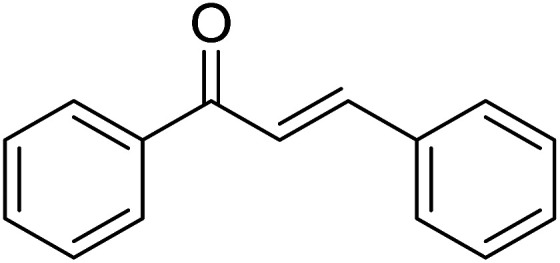	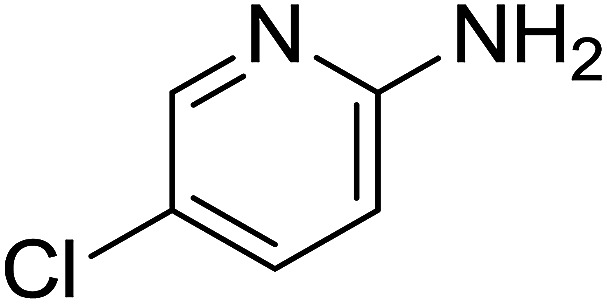	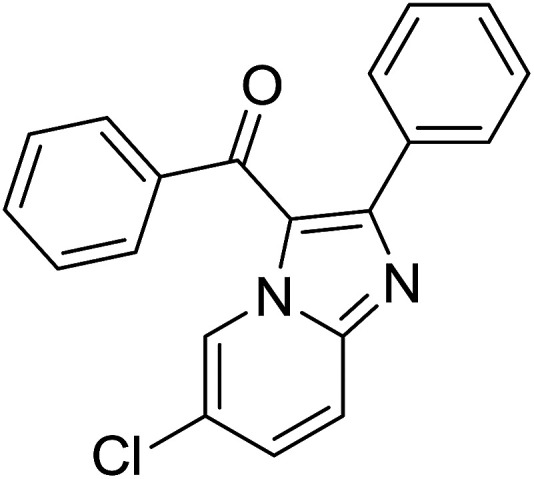	80
13	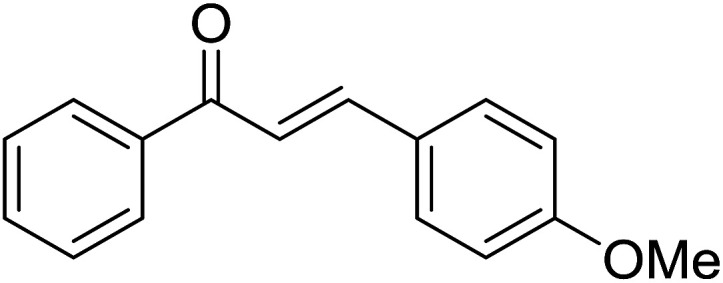	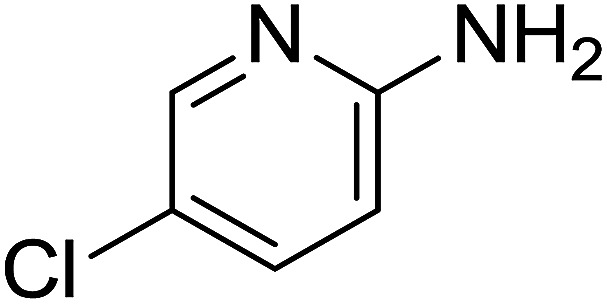	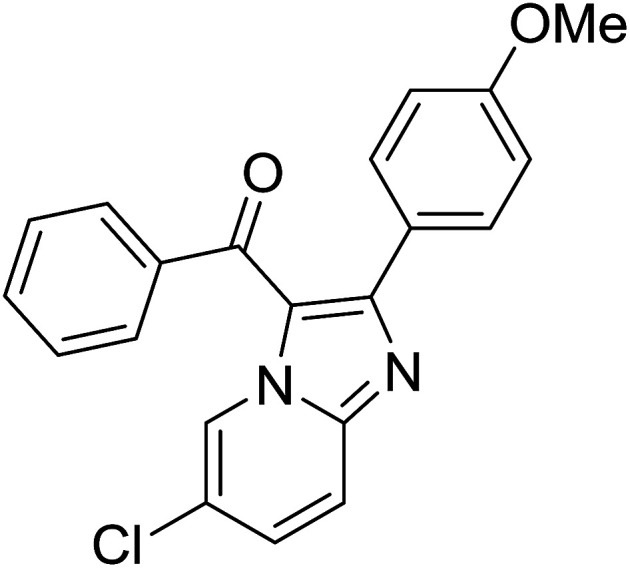	68
14	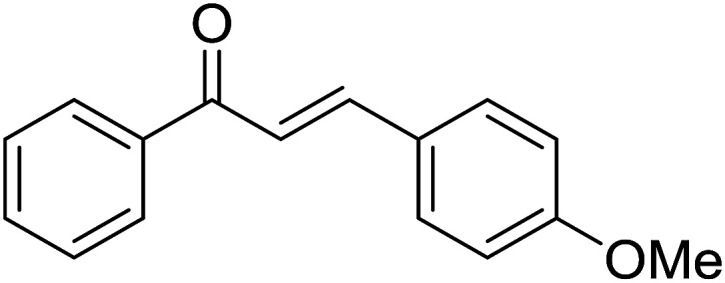	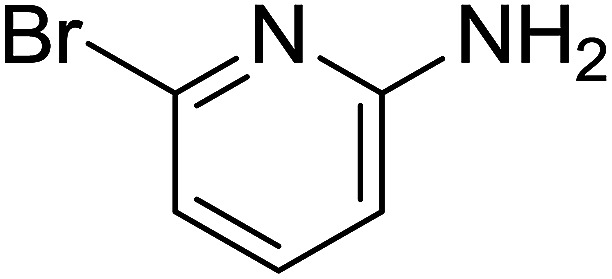	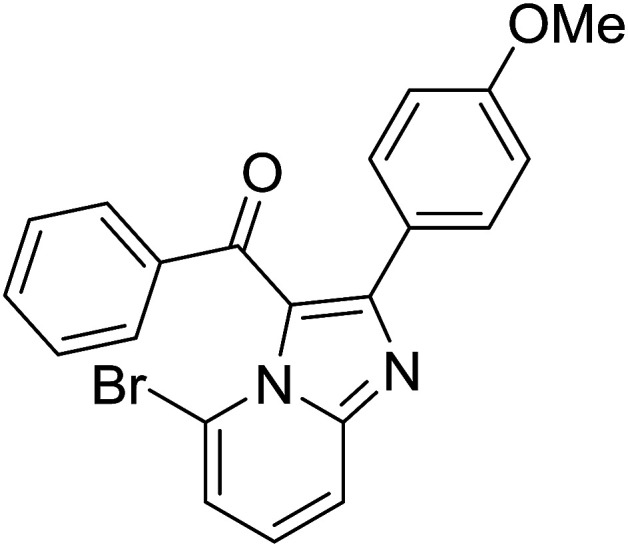	65
15	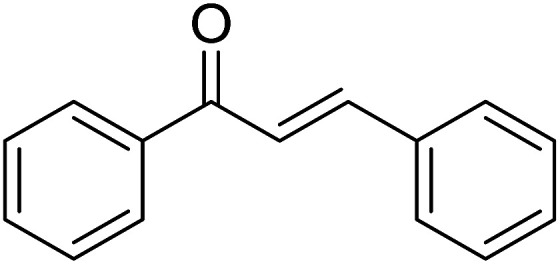	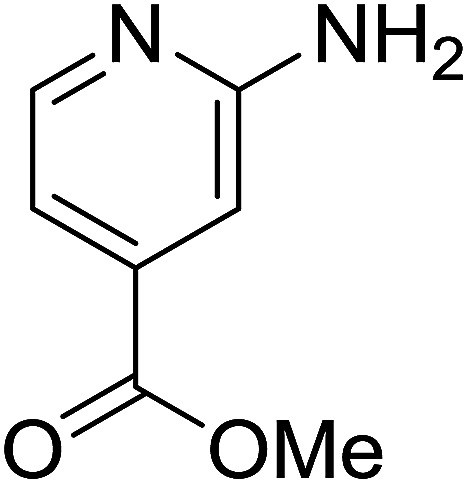	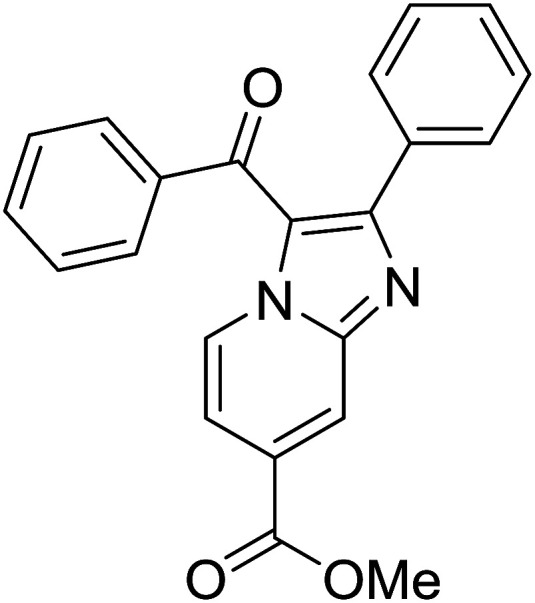	65
16	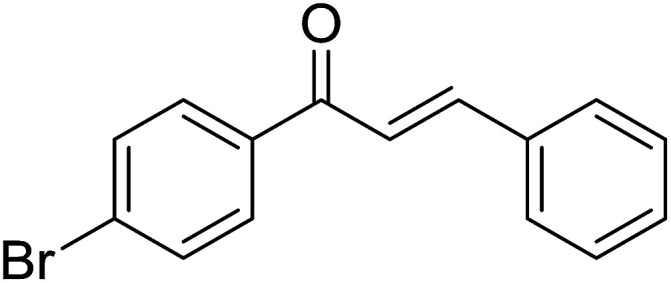	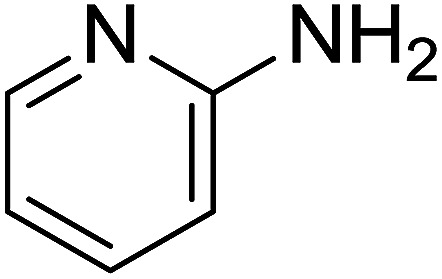	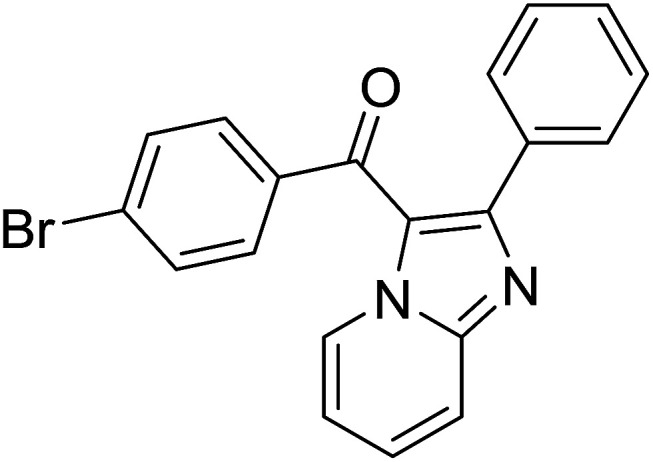	59
17	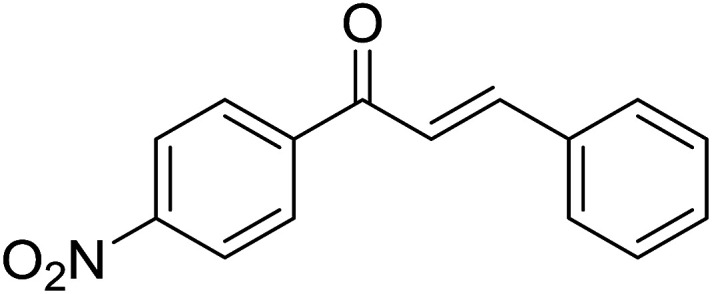	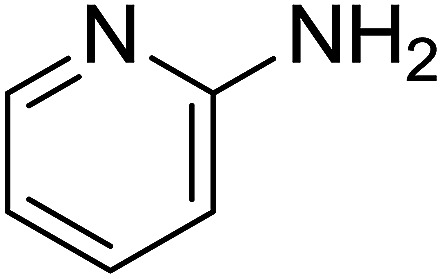	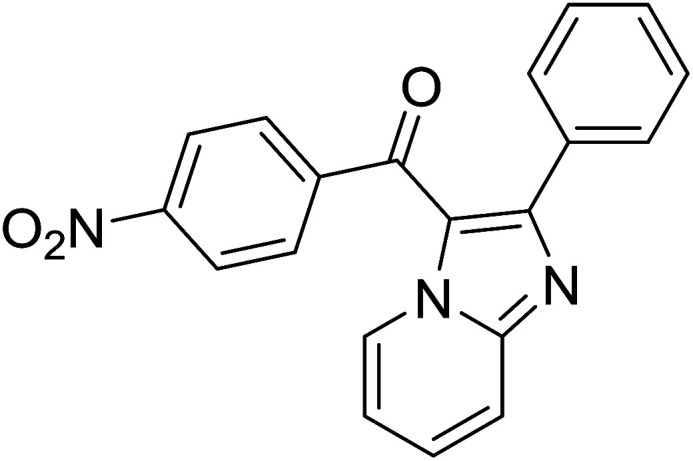	18
18	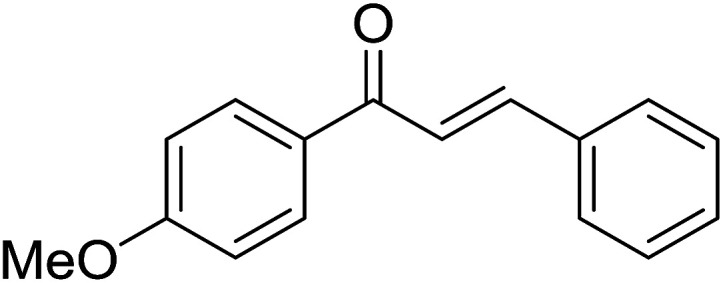	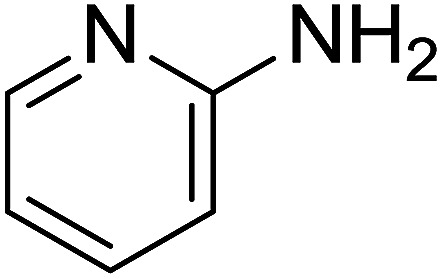	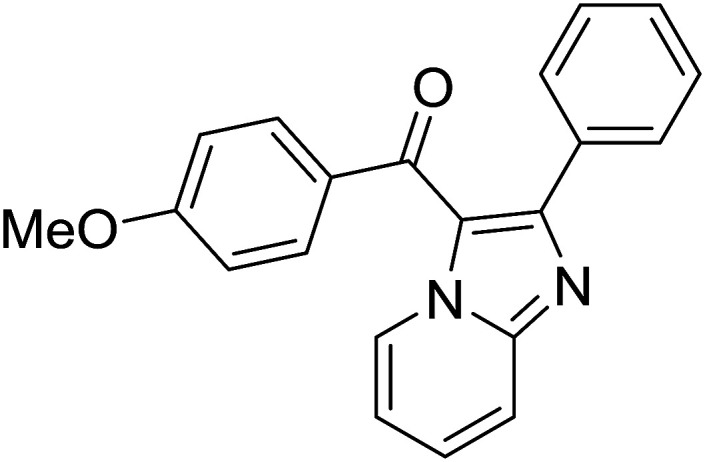	72
19^b^	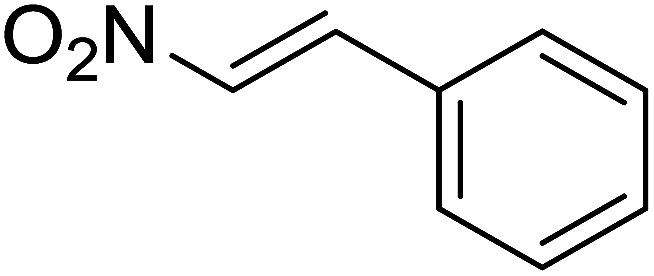	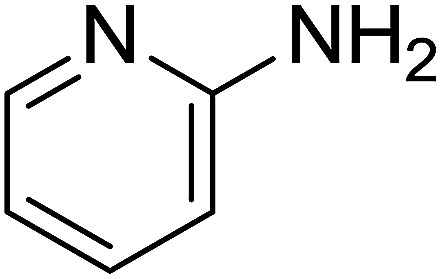	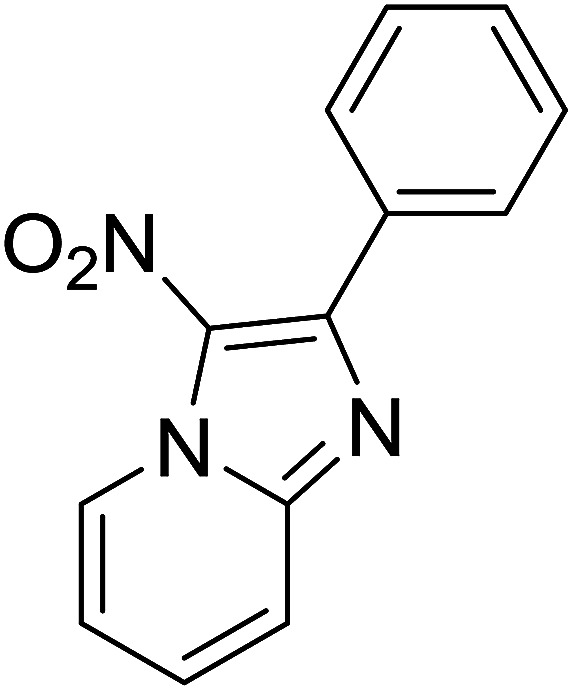	63

a *Trans*-Chalcones (0.3 mmol), 2-aminopyridines or 2-aminopyrimidine (1.6 equiv.), I_2_ (2 equiv.), 1,4-dioxane (2.5 mL), 140 °C, 7 h. Yields are isolated yields. Please see the ESI for details.

b 2-Aminopyridine (0.5 mmol).

**Scheme 2:**
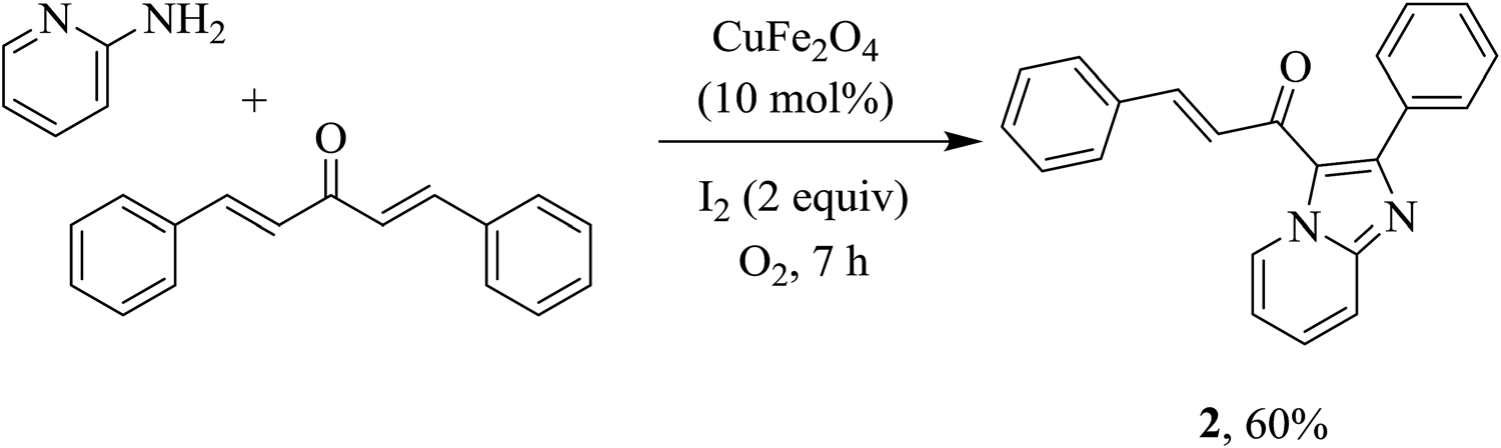
Copper ferrite-catalyzed coupling of dibenzylideneacetone and 2-aminopyridine.

**Scheme 3:**
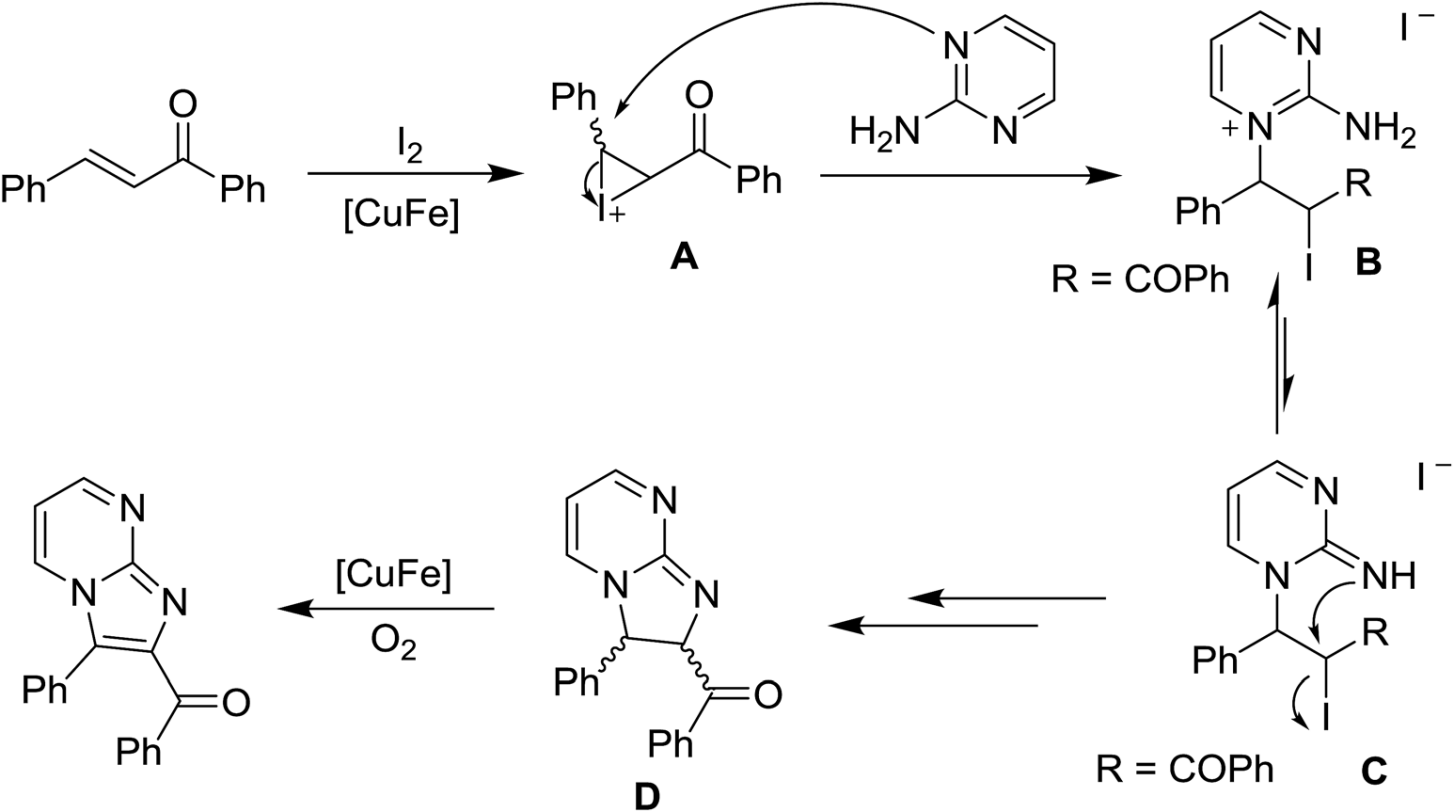
Plausible mechanism.

## 3. Results and discussion

The cyclization of *trans*-chalcone and 2-aminopyrimidine was investigated with respect to reaction temperature, amount of 2-aminopyrimidine, solvents, amount of iodine, catalyst concentration, and different copper or iron salts (Table 1). Only trace amount of the product 1 was obtained if the reaction was run at 60 °C (entry 1). Increasing temperature resulted in better yield (entries 2 and 3). At 140 °C, a complete conversion of *trans*-chalcone was observed. 2-Aminopyrimidine should be slightly excess to afford good yield of 1 (entry 4). Interestingly, using more than two equivalents of 2-aminopyrimidine lowered the yield (entry 5). Strongly coordinated solvents such as DMF or DMSO are not suitable for this reaction (entries 6 and 7), while chlorobenzene is inferior to 1,4-dioxane (entry 8). No product was obtained if I_2_ was omitted (entry 9). Using less than 2 equivalents of I_2_ plummeted the yield of 1 (entries 10 and 11). Low yield was also obtained if the amount of CuFe_2_O_4_ was decreased (entry 12). Running the reaction in the absence of copper ferrite gave 1 in 15% yield (entry 13). The coupling was not successful under argon atmosphere (entry 14), proving that oxygen oxidant is crucial. Such a copper-mediated aerobic transformation is known.^3*a*,3*e*,3*f*^

To highlight the prominent role of CuFe_2_O_4_ for the coupling, uses of other heterogeneous or commercial catalysts were examined. The reaction is tolerant of many copper and iron salts, albeit at lower yields (entries 15–21, Table 1). Interestingly, nanoparticles of copper or iron oxides gave product 1 in extremely low yields (entries 20 and 21). Although we could not rationalize the reason for this phenomenon, the results emphasize the synergistic effect of copper-iron cluster. It should be noted that, Glorius and co-workers, when studying the copper ferrite-catalyzed, direct C–H arylation of heteroarenes, also observed inexplicably low yields when copper and iron oxides were solely used.^7*a*^

Reaction scope is presented in Table 2. Reaction of 2-aminopyrimidine with a chloro derivative of *trans*-chalcone gave the product in 76% yield (entry 1). The conditions are also used to couple 2-aminopyridines with *trans*-chalcones (entries 2–19). Good yields are obtained regardless of electronic properties of *trans*-chalcones (entries 2–14). The reaction tolerates methyl, chloro, bromo, and methoxy groups. That halogenated 2-aminopyridines successfully coupled with *trans*-chalcone (entries 12–14) is helpful since the products could be modified *via* cross-coupling reactions. A 2-aminopyridine bearing carboxylate functionality gave the product in 65% yield (entry 15). With respect to the substituents on the keto moiety of chalcones, electron donating groups were superior to electron withdrawing groups (entries 16–18). The conditions are applied for synthesis of a 3-nitroimidazo[1,2-*a*]pyridine derivative in moderate yield (entry 19).

To confirm whether double annulation is possible or not, a highly conjugated chalcone such as dibenzylideneacetone was used to couple with 2-aminopyridine under standard conditions. The reaction only afforded 60% yield of a mono-coupling product 2 even in the presence of two equivalents of 2-aminopyridine (Scheme 2). Interestingly, a similar result was observed by Hajra and co-workers when a copper-phenanthroline system was used for the reaction.^3*a*^

A possible mechanism to obtain 3-benzoylimidazo[1,2-*a*]pyrimidine is presented in Scheme 3. We speculated that the reaction may proceed through the formation of some α-iodo ketone-typed compounds (A → B ↔ C).^3*d*,3*g*^ The copper iron oxide could increase the electrophilicity of the carbon–carbon double bond through binding, thus helping the first nucleophilic substitution. Oxidation of the α,β carbon–carbon single bond to form the enone-typed compound may also be facilitated by CuFe_2_O_4_ species in the presence of oxygen oxidant. Such a copper-mediated aerobic oxidation has been reported by Hajra and Kumar.^3*a*,3*e*,3*f*^ The rate-determining step is perhaps the oxidation of C–C bond, since low yields were obtained in the case of electron-poor ketones. A possibility of involving iodine-free cyclocuprate species in the mechanism could be ruled out under our conditions,^3*a*^ because running the reaction in the absence of iodine did not afford the desire product.

**Fig. 1:**
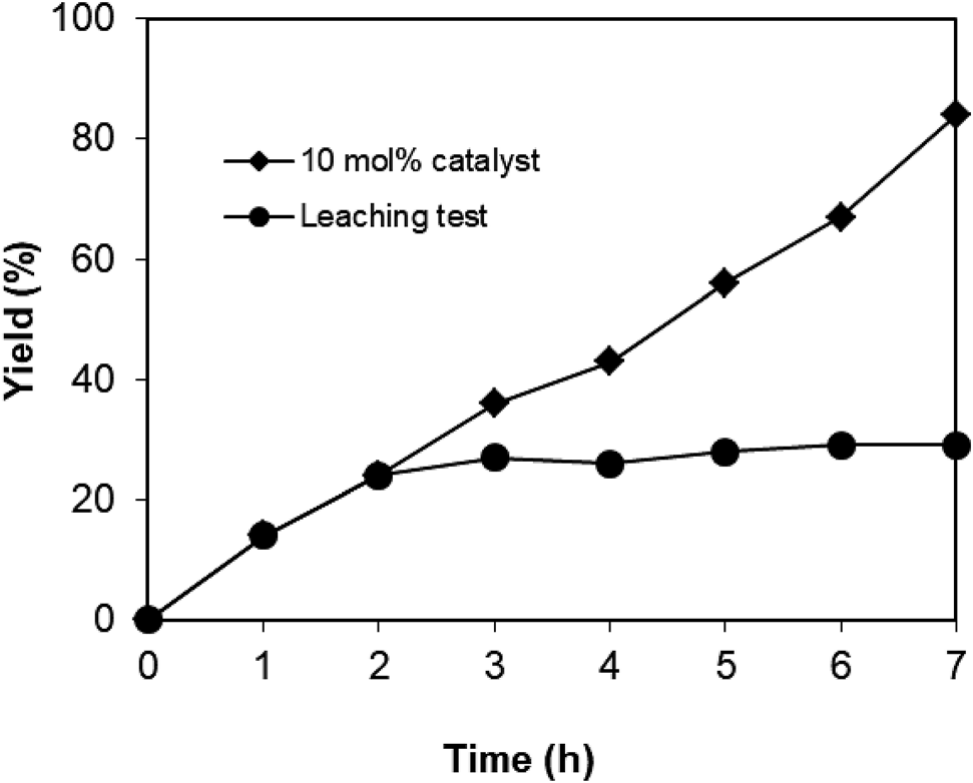
Kinetic profile for the coupling of 2-aminopyrimidine with *trans*-chalcone in the presence of CuFe_2_O_4_. The circle-dot line represents the profile when CuFe_2_O_4_ was filtered after 2 hours.

A hot filtration test was carried out to confirm the heterogeneity of the catalyst, since the formation of product 1 could be possibly attributed to soluble active species.^7*a*^ The reaction of 2-aminopyrimidine and *trans*-chalcone proceeded in the presence of 10 mol% CuFe_2_O_4_, oxygen oxidant, and 1,4-dioxane solvent at 140 °C for 7 h. After two hours, CuFe_2_O_4_ was removed by magnetic decantation. The mother liquor was decanted and transferred to a new pressurized vial, followed by heated for additional 5 h at 140 °C. Yield of 1 did not substantially improve after the catalyst was removed, proving that leaching species if possible is negligible (Fig. 1). Notably, product 1 was obtained in 84% yield after 7 h if the magnetic copper oxide was not separated.

**Fig. 2:**
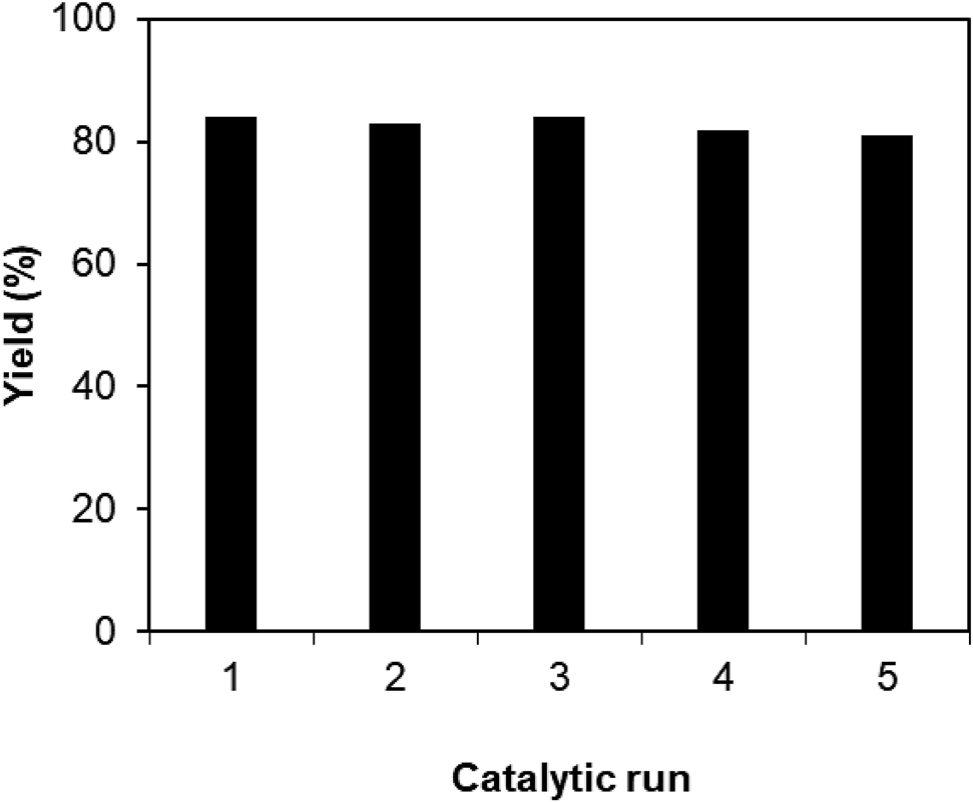
Reuse of copper ferrite for the coupling of 2-aminopyrimidine with *trans*-chalcone.

**Fig. 3:**
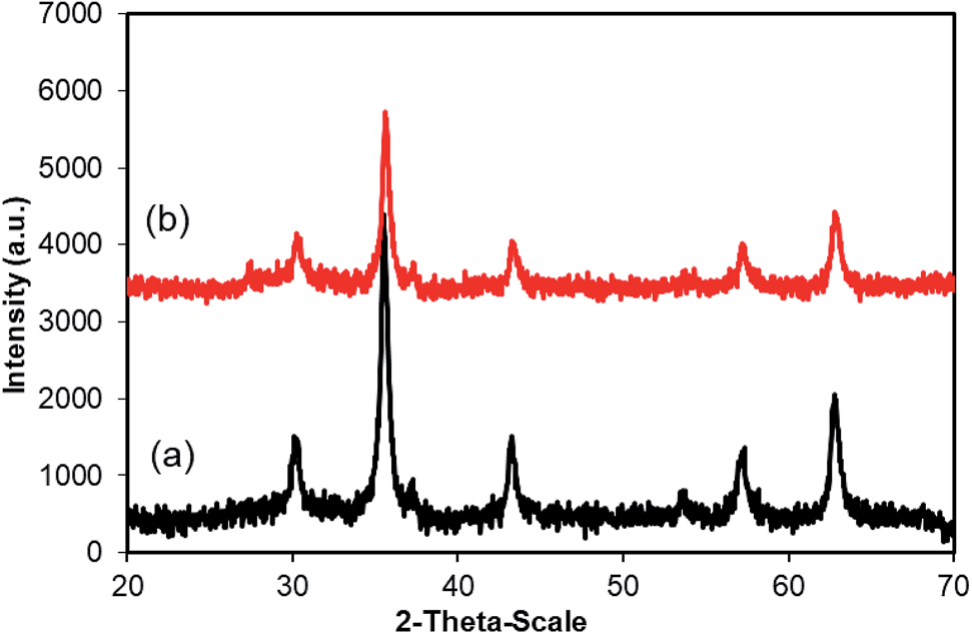
XRD patterns of the fresh (a) and reused (b) CuFe_2_O_4_ for the coupling of 2-aminopyrimidine with *trans*-chalcone.

Furthermore, reusability of the copper ferrite is also investigated. The recovered CuFe_2_O_4_ nanoparticles were carefully washed with ethyl acetate, ethanol, and acetone. The copper ferrite was then heated at 120 °C under strong vacuum for 6 h. The dried particles were then used for new catalytic reactions under standard conditions. The result shows that CuFe_2_O_4_ catalyst could be reused up to 5 times, while yield of the 5^th^ time run was still 81% (Fig. 2). Additionally, XRD analysis of the recovered CuFe_2_O_4_ revealed that the structure of the superparamagnetic nanoparticles was still maintained during the course of the reaction (Fig. 3).

It should be noted that the methods for direct synthesis of aroylimidazo[1,2-*a*]pyridines and aroylimidazo[1,2-*a*]pyrimidines from 2-aminopyridines and 2-aminopyrimidines, respectively, are known. Some typical protocols are presented in Table 3. Until now, most of the methods still suffer from notorious challenges. For example, early works of Hajra or Kumar relied on the use of nonreusable catalyst (CuCl_2_) and/or an external ligand (1,10-phenanthroline).^3*a*,3*f*^ Later, Kumar developed an one-pot method for synthesis of 3-aroylimidazo[1,2-*a*]pyridines which employed the excess amount of inorganic base K_2_CO_3_.^3*e*^ Recently, a transition-metal-free protocol was also presented; however, the practicality of the method is still questionable since the procedure required the use of AlCl_3_ which is extremely moisture sensitive.^3*g*^ Compared to the previous reports, our method would offer a possibility to prepare imidazo[1,2-*a*]pyridines towards green chemistry. The conditions use superparamagnetic CuFe_2_O_4_ nanoparticles, which are viably recoverable, for synthesis of fused N-heterocycles in good yields and high compatibility of functional groups.

**Table 3:** Advantages and disadvantages of available methods for coupling of 2-aminopyridines/2-aminopyrimidines and chalcones

Methods	Pros	Cons	Reference
Cu(OAc)_2_ + 1,10-phenanthroline	Wide scope of substrates	Requirement of ligand	3*a*
		Nonreusable catalyst	
CuCl_2_	Ligandless conditions	Nonreusable catalyst	3*f*
CuCl_2_ + K_2_CO_3_	One pot reaction	Excess inorganic base	3*e*
I_2_ + AlCl_3_	Wide scope of substrates	Moisture sensitivity	3*g*
Our method	Wide scope of substrates		
	Recoverable catalyst		
	No need of ligand and/or base		

## 4. Conclusions

In conclusion, we develop a method for coupling of 2-aminopyridines or 2-aminopyrimidines with *trans*-chalcone to obtain aroylimidazo[1,2-*a*]pyrimidines and aroylimidazo[1,2-*a*]pyridines. Reactions proceed in the presence of CuFe_2_O_4_ catalyst, two equivalents of iodine, and 1,4-dioxane solvent. Functionalities such as methyl, methoxy, chloro, bromo, and ester groups are compatible with reaction conditions. The catalyst is superior to many common copper or iron complexes. The superparamagnetic copper oxide nanoparticles is truly heterogeneous and reusable, up to 5 times, without a major loss of structure or activity. Our method for synthesis of fused N-heterocycles is useful for later studies towards sustainable chemistry.

## Conflicts of interest

There are no conflicts to declare.

## Supplementary Material

RA-009-C9RA00097F-s001
