# Supplementary material for: Superparamagnetic nanoparticle-catalyzed coupling of 2-amino pyridines/pyrimidines with trans-chalcones
Source: RSC Adv. 2019 Feb 13;9(10):5501–11. doi: 10.1039/c9ra00097f (PMC9060791; doi:10.1039/c9ra00097f)
Supplement: RA-009-C9RA00097F-s001 [file RA-009-C9RA00097F-s001.pdf]

Electronic Supplementary Material (ESI) for RSC Advances.
This journal is © The Royal Society of Chemistry 2019

Superparamagnetic nanoparticle-catalyzed coupling of 2-amino pyridines/pyrimidines with *trans*-chalcones

Oanh T. K. Nguyen, Pha T. Ha, Ha V. Dang, Yen H. Vo, Tung T. Nguyen, Nhan T. H. Le,* Nam T. S. Phan*

Faculty of Chemical Engineering, HCMC University of Technology, VNU-HCM,

268 Ly Thuong Kiet, District 10, Ho Chi Minh City, Viet Nam

*Email: lthnhan@hcmut.edu.vn, ptsnam@hcmut.edu.vn

1. Materials and instrumentation

All reagents and starting materials were obtained from Sigma-Aldrich and Merck and were used as received without any further purification unless otherwise noted. X-ray powder diffraction (XRD) patterns were recorded using a Cu Kα radiation source on a D8 Advance Bruker powder diffractometer. Gas chromatographic (GC) analyses were performed using a Shimadzu GC 2010-Plus equipped with a flame ionization detector (FID) and an SPB-5 column (length = 30 m, inner diameter = 0.25 mm, and film thickness = 0.25 μm). In the GC temperature program, the sample of the reaction was held at 100 °C for 1 min, heated from 100 to 280 °C at 40°C/min, and held at 280 °C for 6.5 min. GC yields of the reaction were calculated using diphenyl ether internal standard. GC-MS analyses were performed using a Shimadzu GCMS-QP2010Ultra with a ZB-5MS column (length = 30 m, inner diameter = 0.25 mm, and film thickness = 0.25 μm). The temperature program for GC-MS analysis held samples at 50 °C for 2 min, heated samples from 50 to 280 °C at 10 °C/min, and held them at 280 °C for 10 min. Inlet temperature was set constant at 280 ^{o}C. The spectra were compared with that gathered in the NIST library. The ^{1}H, ^{13}C, and ^{19}F spectra were recorded on Bruker AV 500, JEOL EC-400, or JEOL EC-600 spectrometers using residual solvent peaks as references.

2. Experimental and results

2.1. General procedure

To a pressurized vial was charged *trans*-chalcone (62 mg, 0.3 mmol), 2-aminopyrimidine (48 mg, 0.5 mmol), I_2 (254 mg, 0.6 mmol), $CuFe_2O_4$, diphenyl ether (51 mg, 0.3 mmol)

internal standard, and 1,4-dioxane (2.5 mL). The vial was heated at 140 °C for 7 h under an oxygen atmosphere. The reaction progress was monitored by withdrawing an aliquot of the reaction mixture and quenching with $Na_2S_2O_3$ solution (10% v/v, 1 mL). Organic compounds then were extracted with ethyl acetate (2 mL x 2). Combined organic phases were dried over Na_2SO_4, filtered, and concentrated. Purification by column chromatography afforded desire products.

2.2. Product characterization

Phenyl(2-phenylimidazo[1,2-*a*]pyrimidin-3-yl)methanone (1, Table 1)

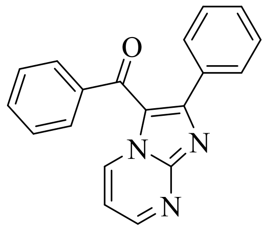

Following the general experimental procedure: *trans*-chalcone (0.3 mmol, 63 mg), 2-aminopyrimidine (0.5 mmol, 48 mg), $CuFe_2O_4$ (30 μmol, 8 mg), I_2 (0.6 mmol, 152 mg), 1,4-dioxane (2.5 mL), 140 °C, 7 h. After column chromatography on silica gel (hexane: ethyl acetate = 2 : 3), 73 mg (81%) of a yellow solid was obtained. This compound is known.[1]

m.p. = 168–170 °C.

^{1}H NMR (500 MHz, $CDCl_3$, ppm) δ 9.77 (dd, *J* = 6.9, 2.0 Hz, 1H), 8.81 (dd, *J* = 4.2, 2.1 Hz, 1H), 7.52 (dd, *J* = 8.2, 1.2 Hz, 2H), 7.41 – 7.37 (m, 2H), 7.30 (dt, *J* = 8.7, 1.2 Hz, 1H), 7.19 – 7.15 (m, 2H), 7.15 – 7.08 (m, 4H).

[1] X. Meng, J. Zhang, B. Chen, Z. Jing, P. Zhao, *Catal. Sci. Technol.* **2016**, *6*, 890.

^{13}C NMR (126 MHz, $CDCl_3$, ppm) δ 187.6, 156.0, 153.5, 150.0, 138.0, 1360, 133.2, 132.2, 130.5, 129.6, 128.8, 127.9, 127.8, 118.2, 110.8.

(2-(4-Chlorophenyl)imidazo[1,2-*a*]pyrimidin-3-yl)(phenyl)methanone (entry 1, Table 2)

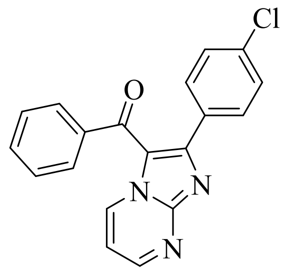

Following the general experimental procedure: 4-chlorochalcone (0.3 mmol, 73 mg), 2-aminopyrimidine (0.5 mmol, 48 mg), $CuFe_2O_4$ (30 μmol, 8 mg), I_2 (0.6 mmol, 152 mg), 1,4-dioxane (2.5 mL), 140 °C, 7 h. After column chromatography on silica gel (hexane: chloroform: ethyl acetate = 80 : 20 : 5), 76 mg (76%) of a yellow solid was obtained.

m.p. = 173–175 °C.

^1H NMR (500 MHz, $CDCl_3$, ppm) δ 9.75 (dd, J = 6.9, 2.1 Hz, 1H), 8.81 (dd, J = 4.2, 2.1 Hz, 1H), 7.55 – 7.49 (m, 2H), 7.40 – 7.35 (m, 1H), 7.35 – 7.32 (m, 2H), 7.20 – 7.14 (m, 3H), 7.11 – 7.06 (m, 2H).

^{13}C NMR (126 MHz, $CDCl_3$, ppm) δ 187.3, 154.5, 153.7, 150.0, 137.9, 136.0, 135.1, 132.5, 131.7, 131.7, 129.6, 128.2, 128.1, 118.2, 110.9.

Phenyl(2-phenylimidazo[1,2-*a*]pyridin-3-yl)methanone (entry 2, Table 2)

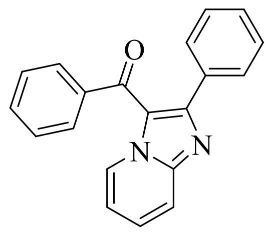

Following the general experimental procedure: *trans*-chalcone (0.3 mmol, 63 mg), 2-aminopyridine (0.5 mmol, 47 mg), $CuFe_2O_4$ (30 μmol, 8 mg), I_2 (0.6 mmol, 152 mg), 1,4-dioxane (2.5 mL), 140 °C, 7 h. After column chromatography on silica gel (hexane: ethyl acetate = 4 : 1), 58 mg (65%) of a yellow solid was obtained. This compound is known.[1]

m.p. = 130–132 °C.

^{1}H NMR (500 MHz, $CDCl_3$, ppm) δ 9.58 (d, *J* = 6.9 Hz, 1H), 7.84 (d, *J* = 8.9 Hz, 1H), 7.58 – 7.53 (m, 3H), 7.35 (d, *J* = 7.0 Hz, 2H), 7.27 (t, *J* = 8 Hz, 1H, overlapping with $CHCl_3$ signal), 7.19 – 7.07 (m, 6H).

^{13}C NMR (126 MHz, $CDCl_3$, ppm) δ 187.4, 154.9, 147.4, 138.6, 133.9, 131.8, 130.2, 129.6, 129.3, 128.3, 127.8, 120.0, 117.5, 114.7. Two carbon signals could not be located.

(2-(4-Chlorophenyl)imidazo[1,2-*a*]pyridin-3-yl)(phenyl)methanone (entry 3, Table 2)

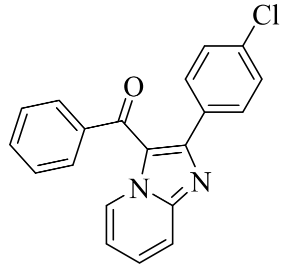

Following the general experimental procedure: 4-chlorochalcone (0.3 mmol, 73 mg), 2-aminopyridine (0.5 mmol, 47 mg), $CuFe_2O_4$ (30 μmol, 8 mg), I_2 (0.6 mmol, 152 mg), 1,4-

dioxane (2.5 mL), 140 °C, 7 h. After column chromatography on silica gel (hexane: chloroform: ethyl acetate = 80 : 20 : 5), 67 mg (67%) of a yellow solid was obtained. This compound is known.[1]

m.p. = 122–123 °C.

^{1}H NMR (500 MHz, $CDCl_3$, ppm) δ 9.53 (dt, J = 7.0, 1.1 Hz, 1H), 7.80 (dt, J = 9.0, 1.1 Hz, 1H), 7.54 (ddd, J = 8.9, 6.9, 1.3 Hz, 1H), 7.52 – 7.49 (m, 2H), 7.36 – 7.31 (m, 1H), 7.27 (t, J = 2.2 Hz, 1H, overlapping with $CHCl_3$ signal), 7.25 – 7.24 (m, 1H, overlapping with $CHCl_3$ signal), 7.16 – 7.09 (m, 3H), 7.08 – 7.04 (m, 2H).

^{13}C NMR (126 MHz, $CDCl_3$, ppm) δ 187.2, 153.6, 147.5, 138.6, 134.5, 132.6, 132.0, 131.4, 129.6, 129.4, 128.3, 128.0, 120.1, 117.5, 114.8. One carbon signal could not be located.

(2-(4-Methoxyphenyl)imidazo[1,2-*a*]pyridin-3-yl)(phenyl)methanone (entry 4, Table 2)

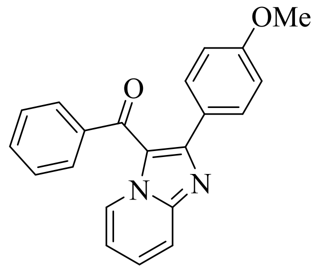

Following the general experimental procedure: 4-methoxychalcone (0.3 mmol, 72 mg), 2-aminopyridine (0.5 mmol, 47 mg), $CuFe_2O_4$ (30 μmol, 8 mg), I_2 (0.6 mmol, 152 mg), 1,4-dioxane (2.5 mL), 140 °C, 7 h. After column chromatography on silica gel (hexane:

ethyl acetate = 4 : 1), 67 mg (68%) of a yellow solid was obtained. This compound is known.[1]

m.p. = 153–155 °C.

^{1}H NMR (500 MHz, $CDCl_3$, ppm) δ 9.53 (dt, *J* = 7.0, 1.1 Hz, 1H), 7.78 (dt, *J* = 8.9, 1.1 Hz, 1H), 7.56 – 7.46 (m, 3H), 7.31 – 7.23 (m, 3H, overlapping with $CHCl_3$ signal), 7.15 – 7.09 (m, 2H), 7.07 (td, J = 6.9, 1.3 Hz, 1H), 6.62 – 6.61 (m, 2H), 3.72 (s, 3H).

^{13}C NMR (126 MHz, $CDCl_3$, ppm) δ 187.4, 159.8, 154.8, 147.5, 138.7, 131.7, 131.6, 129.6, 129.1, 128.3, 127.8, 126.5, 119.7, 117.3, 114.4, 113.3, 55.3.

(7-Methyl-2-phenylimidazo[1,2-*a*]pyridin-3-yl)(phenyl)methanone (entry 5, Table 2)

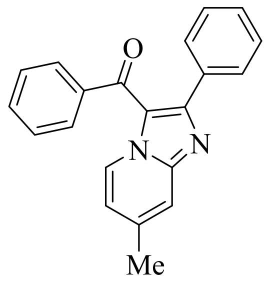

Following the general experimental procedure: *trans*-chalcone (0.3 mmol, 63 mg), 4-methylpyridin-2-amine (0.5 mmol, 54 mg), $CuFe_2O_4$ (30 μmol, 8 mg), I_2 (0.6 mmol, 152 mg), 1,4-dioxane (2.5 mL), 140 °C, 7 h. After column chromatography on silica gel (hexane: ethyl acetate = 3 : 2), 70 mg (75%) of a yellow solid was obtained. This compound is known.[2]

m.p. = 139–140 °C.

[2] K. Monir, A. K. Bagdi, S. Mishra, A. Majee, A. Hajra, *Adv. Synth. Catal.* **2014**, *356*, 1105.

^{1}H NMR (500 MHz, $CDCl_3$, ppm) δ 9.40 (s, 1H), 7.74 (d, J = 9.0 Hz, 1H), 7.53 (d, J = 7.7 Hz, 2H), 7.42 (d, J = 9.0 Hz, 1H), 7.33 (d, J = 7.3 Hz, 2H), 7.28 (t, J = 7.5 Hz, 1H, overlapping with $CHCl_3$ signal), 7.12 (m, 5H), 2.48 (s, 3H).

^{13}C NMR (126 MHz, $CDCl_3$, ppm) δ 187.3, 154.7, 146.3, 138.8, 134.0, 132.2, 131.7, 130.2, 129.6, 128.2, 127.7, 126.2, 124.7, 119.9, 116.7, 18.5. One carbon signal could not be located.

(7-Methyl-2-(4-chlorophenyl)imidazo[1,2-*a*]pyridin-3-yl)(phenyl)methanone (entry 6, Table 2)

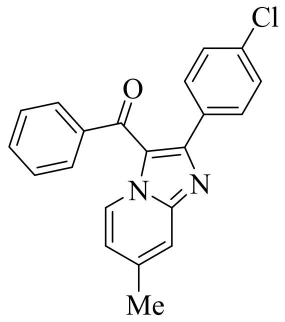

Following the general experimental procedure: 4-chlorochalcone (0.3 mmol, 73 mg), 4-methylpyridin-2-amine (0.5 mmol, 54 mg), $CuFe_2O_4$ (30 μmol, 8 mg), I_2 (0.6 mmol, 152 mg), 1,4-dioxane (2.5 mL), 140 °C, 7 h. After column chromatography on silica gel (5% ethyl acetate in a mixture of 5:1 hexane: chloroform), 72 mg (69%) of a yellow solid was obtained. This compound is known.[2]

m.p. = 123–125 °C.

^{1}H NMR (500 MHz, $CDCl_3$, ppm) δ 9.43 (d, J = 7.1 Hz, 1H), 7.59 – 7.53 (m, 1H), 7.49 – 7.44 (m, 2H), 7.32 (t, J = 7.5 Hz, 1H), 7.25 – 7.20 (m, 2H), 7.16 – 7.09 (m, 2H), 7.07 – 7.01 (m, 2H), 6.94 (dd, J = 7.1, 1.7 Hz, 1H), 2.52 (s, 3H).

^{13}C NMR (126 MHz, $CDCl_3$, ppm) δ 186.9, 153.9, 147.9, 141.1, 138.8, 134.4, 132.8, 131.8, 131.4, 129.5, 127.9, 127.5, 117.3, 116.1, 89.7, 21.6. One carbon signal could not be located.

(7-Methyl-2-(4-methoxyphenyl)imidazo[1,2-*a*]pyridin-3-yl)(phenyl)methanone (entry 7, Table 2)

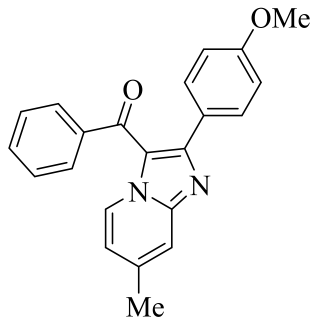

Following the general experimental procedure: 4-methoxychalcone (0.3 mmol, 72 mg), 4-methylpyridin-2-amine (0.5 mmol, 54 mg), $CuFe_2O_4$ (30 μmol, 8 mg), I_2 (0.6 mmol, 152 mg), 1,4-dioxane (2.5 mL), 140 °C, 7 h. After column chromatography on silica gel (hexane: ethyl acetate = 1:1), 72 mg (70%) of a yellow solid was obtained.

m.p. = 160–162 °C.

^{1}H NMR (500 MHz, $CDCl_3$, ppm) δ 9.44 (d, J = 7.1 Hz, 1H), 7.53 (s, 1H), 7.50 (dd, J = 8.2, 1.1 Hz, 2H), 7.28 – 7.23 (m, 3H, overlapping with $CHCl_3$ signal), 7.11 (t, J = 7.8 Hz, 2H), 6.91 (dd, J = 7.1, 1.6 Hz, 1H), 6.64 – 6.55 (m, 2H), 3.72 (s, 3H), 2.51 (s, 3H).

^{13}C NMR (126 MHz, $CDCl_3$, ppm) δ 187.1, 159.7, 155.2, 147.9, 140.8, 138.9, 131.5, 129.6, 127.8, 127.5, 126.6, 119.5, 116.9, 115.9, 113.3, 55.2, 21.6. One carbon signal could not be located.

(6-Methyl-2-phenylimidazo[1,2-*a*]pyridin-3-yl)(phenyl)methanone (entry 8, Table 2)

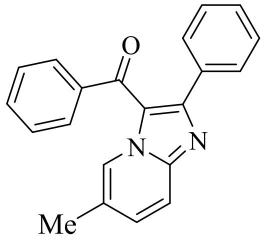

Following the general experimental procedure: *trans*-chalcone (0.3 mmol, 63 mg), 5-methylpyridin-2-amine (0.5 mmol, 54 mg), $CuFe_2O_4$ (30 µmol, 8 mg), I_2 (0.6 mmol, 152 mg), 1,4-dioxane (2.5 mL), 140 °C, 7 h. After column chromatography on silica gel (hexane: ethyl acetate = 3:2), 67 mg (71%) of a yellow solid was obtained. This compound is known.[1]

m.p. = 162–163 °C.

^{1}H NMR (500 MHz, $CDCl_3$, ppm) δ 9.38 (s, 1H), 7.71 (d, J = 9.0 Hz, 1H), 7.51 (dd, J = 8.2, 1.1 Hz, 2H), 7.38 (dd, J = 9.0, 1.4 Hz, 1H), 7.31 (dd, J = 8.1, 1.2 Hz, 2H), 7.24 (d, J = 7.5 Hz, 1H), 7.15 – 7.03 (m, 5H), 2.45 (s, 3H).

^{13}C NMR (126 MHz, $CDCl_3$, ppm) δ 187.3, 154.9, 146.5, 138.8, 134.2, 132.1, 131.7, 130.2, 129.6, 128.1, 127.71, 127.70, 126.2, 124.6, 119.9, 116.7, 18.5.

(6-Methyl-2-phenylimidazo[1,2-*a*]pyridin-3-yl)(phenyl)methanone (entry 9, Table 2)

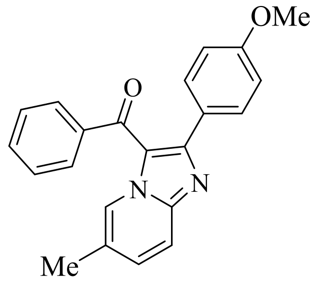

Following the general experimental procedure: 4-methoxychalcone (0.3 mmol, 72 mg), 5-methylpyridin-2-amine (0.5 mmol, 54 mg), $CuFe_2O_4$ (30 μmol, 8 mg), I_2 (0.6 mmol, 152 mg), 1,4-dioxane (2.5 mL), 140 °C, 7 h. After column chromatography on silica gel (hexane: ethyl acetate = 3:2), 77 mg (75%) of a yellow solid was obtained.

m.p. = 178–180 °C.

^{1}H NMR (500 MHz, $CDCl_3$, ppm) δ 9.36 (dt, *J* = 1.8, 1.0 Hz, 1H), 7.70 – 7.65 (m, 1H), 7.54 – 7.49 (m, 2H), 7.37 (dd, *J* = 9.0, 1.7 Hz, 1H), 7.30 – 7.23 (m, 3H, overlapping with $CHCl_3$ signal), 7.16 – 7.08 (m, 2H), 6.63 – 6.58 (m, 2H), 3.72 (s, 3H), 2.44 (s, 3H).

^{13}C NMR (126 MHz, $CDCl_3$, ppm) δ 187.4, 159.6, 154.7, 146.4, 138.8, 132.1, 131.6, 131.5, 129.6, 127.8, 126.6, 126.2, 124.4, 119.6, 116.5, 113.3, 55.2, 18.5.

(2-(4-Chlorophenyl)-6-methylimidazo[1,2-*a*]pyridin-3-yl)(phenyl)methanone (entry 10, Table 2)

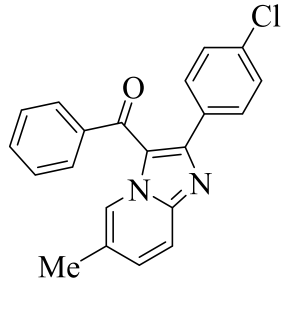

Following the general experimental procedure: 4-chlorochalcone (0.3 mmol, 73 mg), 5-methylpyridin-2-amine (0.5 mmol, 54 mg), $CuFe_2O_4$ (30 μmol, 8 mg), I_2 (0.6 mmol, 152 mg), 1,4-dioxane (2.5 mL), 140 °C, 7 h. After column chromatography on silica gel (hexane: ethyl acetate = 3:2), 78 mg (75%) of a yellow solid was obtained.

m.p. = 144–146°C.

^{1}H NMR (500 MHz, $CDCl_3$, ppm) δ 9.35 (s, 1H), 7.69 (d, J = 9.0 Hz, 1H), 7.49 (d, J = 8.1 Hz, 2H), 7.39 (d, J = 9.0 Hz, 1H), 7.36 – 7.30 (m, 1H), 7.24 (d, J = 8.4 Hz, 2H), 7.13 (t, J = 7.5 Hz, 2H), 7.05 (d, J = 8.3 Hz, 2H), 2.45 (s, 3H).

^{13}C NMR (126 MHz, $CDCl_3$, ppm) δ 187.1, 153.4, 146.5, 138.7, 134.3, 132.8, 132.3, 131.9, 131.3, 129.6, 127.9, 126.2, 124.8, 119.9, 116.7, 18.5.

(2-(4-Chlorophenyl)-8-methylimidazo[1,2-*a*]pyridin-3-yl)(phenyl)methanone (entry 11, Table 2)

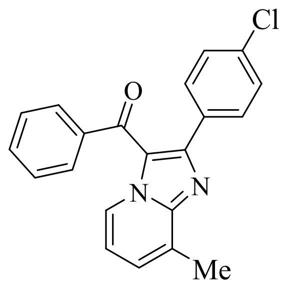

Following the general experimental procedure: 4-chlorochalcone (0.3 mmol, 73 mg), 3-methylpyridin-2-amine (0.5 mmol, 54 mg), $CuFe_2O_4$ (30 μmol, 8 mg), I_2 (0.6 mmol, 152 mg), 1,4-dioxane (2.5 mL), 140 °C, 7 h. After column chromatography on silica gel (hexane: chloroform = 3:1), 76 mg (73%) of a yellow solid was obtained. This compound is known.[2]

m.p. = 123–125°C.

^{1}H NMR (500 MHz, $CDCl_3$, ppm) δ 9.39 (d, *J* = 6.9 Hz, 1H), 7.49 (dd, *J* = 8.2, 1.2 Hz, 2H), 7.35 – 7.29 (m, 2H), 7.29 – 7.24 (m, 2H, overlapping with $CHCl_3$ signal), 7.16 – 7.10 (dt, *J* = 6.5, 1.5 Hz, 2H), 7.08 – 7.04 (m, 2H), 7.02 (t, *J* = 7.0 Hz, 1H), 2.73 (s, 3H).

^{13}C NMR (126 MHz, $CDCl_3$, ppm) δ 187.3, 153.1, 147.7, 138.7, 134.3, 132.9, 131.9, 131.5, 129.5, 128.3, 127.93, 127.90, 127.6, 126.0, 120.5, 114.8, 17.1.

(6-Chloro-2-phenylimidazo[1,2-*a*]pyridin-3-yl)(phenyl)methanone (entry 12, Table 2)

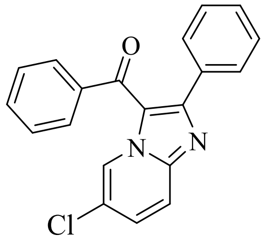

Following the general experimental procedure: *trans*-chalcone (0.3 mmol, 63 mg), 5-chloropyridin-2-amine (0.5 mmol, 64 mg), $CuFe_2O_4$ (30 μmol, 8 mg), I_2 (0.6 mmol, 152 mg), 1,4-dioxane (2.5 mL), 140 °C, 7 h. After column chromatography on silica gel (hexane: ethyl acetate = 3:2), 80 mg (80%) of a yellow solid was obtained. This compound is known.[2]

m.p. = 121–123 °C.

^{1}H NMR (500 MHz, $CDCl_3$, ppm) δ 9.62 (d, *J* = 1.4 Hz, 1H), 7.75 (d, *J* = 9.4 Hz, 1H), 7.54 – 7.47 (m, 3H), 7.35 – 7.27 (m, 3H), 7.18 – 7.06 (m, 5H).

^{13}C NMR (126 MHz, $CDCl_3$, ppm) δ 187.4, 155.1, 145.7, 138.2, 133.6, 132.1, 130.4, 130.2, 129.6, 128.5, 127.85, 127.84, 126.3, 122.9, 117.7. One carbon signal could not be located.

(6-Chloro-2-(4-methoxyphenyl)imidazo[1,2-*a*]pyridin-3-yl)(phenyl)methanone (entry 13, Table 2)

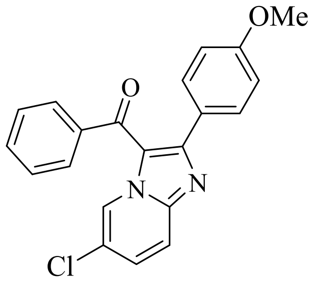

Following the general experimental procedure: 4-methoxychalcone (0.3 mmol, 72 mg), 5-chloropyridin-2-amine (0.5 mmol, 64 mg), $CuFe_2O_4$ (30 μmol, 8 mg), I_2 (0.6 mmol, 152 mg), 1,4-dioxane (2.5 mL), 140 °C, 7 h. After column chromatography on silica gel (hexane: ethyl acetate = 3:2), 74 mg (68%) of a yellow solid was obtained.

m.p. = 137–138 °C.

^{1}H NMR (500 MHz, $CDCl_3$, ppm) δ 9.60 (dd, J = 2.0, 0.8 Hz, 1H), 7.71 (dd, J = 9.4, 0.7 Hz, 1H), 7.54 – 7.50 (m, 2H), 7.47 (dd, J = 9.4, 2.1 Hz, 1H), 7.30 (t, J = 7.4 Hz, 1H), 7.26 – 7.22 (m, 2H), 7.16 – 7.07 (m, 2H), 6.65 – 6.58 (m, 2H), 3.72 (s, 3H).

^{13}C NMR (126 MHz, $CDCl_3$, ppm) δ 187.4, 159.9, 154.9, 145.7, 138.2, 132.0, 131.5, 130.3, 129.6, 127.9, 126.2, 126.0, 122.6, 119.9, 117.5, 113.4, 55.3.

(5-Bromo-2-(4-methoxyphenyl)imidazo[1,2-*a*]pyridin-3-yl)(phenyl)methanone (entry 14, Table 2)

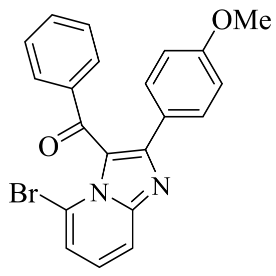

Following the general experimental procedure: 4-methoxychalcone (0.3 mmol, 72 mg), 6-bromopyridin-2-amine (0.5 mmol, 87 mg), $CuFe_2O_4$ (30 μmol, 8 mg), I_2 (0.6 mmol, 152 mg), 1,4-dioxane (2.5 mL), 140 °C, 7 h. After column chromatography on silica gel (hexane: ethyl acetate = 3:2), 79 mg (65%) of a yellow solid was obtained.

m.p. = 149–151 °C.

^{1}H NMR (500 MHz, $CDCl_3$, ppm) δ 9.54 (dt, *J* = 7.0, 1.1 Hz, 1H), 7.78 (dt, *J* = 8.9, 1.1 Hz, 1H), 7.55 – 7.49 (m, 3H), 7.30 – 7.26 (m, 1H, overlapping with $CHCl_3$ signal), 7.26–7.25 (m, 1H) 7.15 – 7.10 (m, 2H), 7.07 (td, *J* = 6.9, 1.3 Hz, 1H), 6.66 – 6.56 (m, 2H), 3.72 (s, 3H).

^{13}C NMR (126 MHz, $CDCl_3$, ppm) δ 187.4, 159.8, 154.8, 147.4, 138.7, 131.7, 131.5, 129.6, 129.1, 128.2, 127.8, 126.5, 119.7, 117.3, 114.4, 113.3, 55.2.

Methyl 3-benzoyl-2-phenylimidazo[1,2-*a*]pyridine-7-carboxylate (entry 15, Table 2)

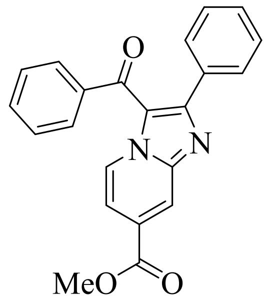

Following the general experimental procedure: *trans*-chalcone (0.3 mmol, 63 mg), methyl 2-aminopyridine-4-carboxylate (0.5 mmol, 76 mg), $CuFe_2O_4$ (30 µmol, 8 mg), I_2 (0.6 mmol, 152 mg), 1,4-dioxane (2.5 mL), 140 °C, 7 h. After column chromatography on silica gel (hexane: ethyl acetate = 1:1), 69 mg (65%) of a green foam was obtained. For this reason, melting point of the compound could not be recorded.

^{1}H NMR (500 MHz, $CDCl_3$, ppm) δ 9.51 – 9.45 (m, 1H), 8.49 (s, 1H), 7.66 (dd, *J* = 7.2, 1.7 Hz, 1H), 7.55 – 7.51 (m, 2H), 7.37 – 7.33 (m, 2H), 7.29 (t, *J* = 7.4 Hz, 1H), 7.18 – 7.14 (m, 1H), 7.14 – 7.08 (m, 4H), 4.02 (s, 3H).

(4-Bromophenyl)(2-phenylimidazo[1,2-*a*]pyridin-3-yl)methanone (entry 16, Table 2)

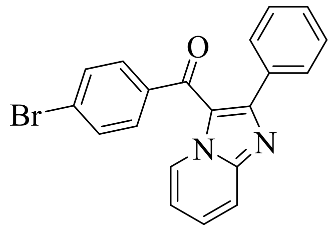

Following the general experimental procedure: 4'-bromochalcone (0.3 mmol, 71 mg), 2-aminopyridine (0.5 mmol, 47 mg), $CuFe_2O_4$ (30 µmol, 8 mg), I_2 (0.6 mmol, 152 mg), 1,4-dioxane (2.5 mL), 140 °C, 7 h. After column chromatography on silica gel (hexane: ethyl acetate = 1:1), 71 mg (65%) of a yellow oil was obtained. This compound is known.[3]

^{1}H NMR (500 MHz, $CDCl_3$, ppm) δ 9.54 (d, *J* = 6.9 Hz, 1H), 7.82 (d, *J* = 8.9 Hz, 1H), 7.59 – 7.52 (m, 1H), 7.38 – 7.33 (m, 2H), 7.33 – 7.28 (m, 2H), 7.24 – 7.18 (m, 3H), 7.12 (dt, *J* = 6.7, 4.4 Hz, 3H).

[3] M.-M. Xing, M. Xin, C. Shen, J.-R. Gao, J.-H. Jia, Y.-J. Li, *Tetrahedron* **2016**, *72*, 4201.

^{13}C NMR (126 MHz, $CDCl_3$, ppm) δ 186.0, 155.3, 147.6, 137.5, 133.8, 131.00, 130.97, 130.2, 129.5, 128.5, 128.3, 127.9, 126.5, 119.9, 117.6, 114.8.

(4-Nitrophenyl)(2-phenylimidazo[1,2-*a*]pyridin-3-yl)methanone (entry 17, Table 2)

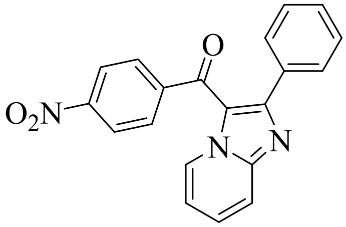

Following the general experimental procedure: 4'-nitrochalcone (0.3 mmol, 76 mg), 2-aminopyridine (0.5 mmol, 47 mg), $CuFe_2O_4$ (30 μmol, 8 mg), I_2 (0.6 mmol, 152 mg), 1,4-dioxane (2.5 mL), 140 °C, 7 h. After column chromatography on silica gel (hexane: ethyl acetate = 1:1), 19 mg (18%) of a yellow foam was obtained. For this reason, melting point of the compound could not be recorded. This compound is known.[3]

^{1}H NMR (500 MHz, $CDCl_3$, ppm) δ 9.67 (d, J = 7 Hz, 1H), 7.92 (d, J = 8.7 Hz, 2H), 7.86 (d, J = 8.9 Hz, 1H), 7.65 – 7.56 (m, 3H), 7.26 (d, J = 7 Hz, 2H, overlapping with $CHCl_3$ signal), 7.22 – 7.13 (m, 2H), 7.09 (t, J = 7.5 Hz, 2H).

^{13}C NMR (126 MHz, $CDCl_3$, ppm) δ 184.7, 156.4, 149.0, 148.0, 144.3, 133.6, 130.3, 130.23, 130.19, 129.0, 128.6, 128.0, 122.8, 119.9, 117.7, 115.4.

(4-Methoxyphenyl)(2-phenylimidazo[1,2-*a*]pyridin-3-yl)methanone (entry 18, Table 2)

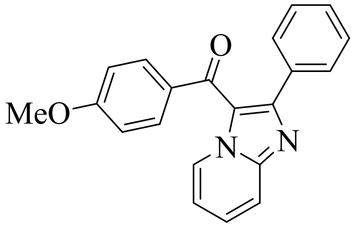

Following the general experimental procedure: 4’-methoxychalcone (0.3 mmol, 76 mg), 2-aminopyridine (0.5 mmol, 47 mg), $CuFe_2O_4$ (30 μmol, 8 mg), I_2 (0.6 mmol, 152 mg), 1,4-dioxane (2.5 mL), 140 °C, 7 h. After column chromatography on silica gel (hexane: ethyl acetate = 1:1), 71 mg (72%) of a yellow solid was obtained. This compound is known.[3]

m.p. = 145–147 °C.

^{1}H NMR (500 MHz, $CDCl_3$, ppm) δ 9.39 (d, J = 7.0 Hz, 1H), 7.80 (d, J = 8.9 Hz, 1H), 7.57 – 7.53 (m, 2H), 7.50 (d, J = 8.1 Hz, 1H), 7.38 (dd, J = 7.7, 1.4 Hz, 2H), 7.15 (dd, J = 8.8, 7.0 Hz, 3H), 7.08 – 7.02 (m, 1H), 6.60 (d, J = 8.8 Hz, 2H), 3.73 (s, 3H).

3-Nitro-2-phenylimidazo[1,2-*a*]pyridine (entry 19, Table 2)

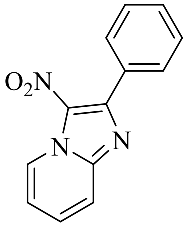

Following the general experimental procedure: *trans*-β-nitrostyrene (0.5 mmol, 75 mg), 2-aminopyridine (0.8 mmol, 75 mg), $CuFe_2O_4$ (50 μmol, 12 mg), I_2 (1 mmol, 254 mg), 1,4-dioxane (4 mL), 140 °C, 7 h. After column chromatography on silica gel (hexane:

ethyl acetate = 2:1), 75 mg (63%) of a yellow solid was obtained. This compound is known.[4]

m.p. = 170–172 °C.

^{1}H NMR (500 MHz, $CDCl_3$) δ 9.51 (d, *J* = 7.0 Hz, 1H), 7.91 (dd, *J* = 6.2, 2.8 Hz, 2H), 7.84 (d, *J* = 8.9 Hz, 1H), 7.66 (t, *J* = 7.7 Hz, 1H), 7.54 – 7.46 (m, 3H), 7.27 (d, *J* = 8.8 Hz, 1H).

^{13}C NMR (126 MHz, $CDCl_3$) δ 150.3, 145.2, 131.9, 130.9, 130.2, 130.1, 128.7, 128.2, 126.1, 118.3, 116.5.

(*E*)-3-Phenyl-1-(2-phenylimidazo[1,2-*a*]pyridin-3-yl)prop-2-en-1-one (2, Scheme 2)

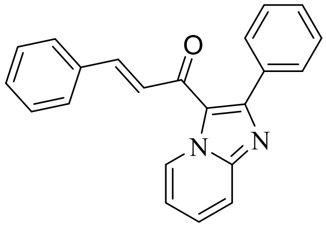

Following the general experimental procedure: dibenzylideneacetone (0.4 mmol, 94 mg), 2-aminopyridine (0.8 mmol, 75 mg), $CuFe_2O_4$ (40 μmol, 10 mg), I_2 (0.8 mmol, 203 mg), 1,4-dioxane (3 mL), 140 °C, 7 h. After column chromatography on silica gel (hexane: ethyl acetate = 10:1), 78 mg (60%) of a yellow oil which slowly solidifies was obtained. For this reason, melting point of the compound could not be recorded. This compound is known.[2]

[4] Y. Tachikawa, Y. Nagasawa, S. Furuhashi, L. Cui, E. Yamaguchi, N. Tada, T. Miura, A. Itoh, *RSC Adv.* **2015**, *5*, 9591.

^{1}H NMR (500 MHz, $CDCl_3$, ppm) δ 9.86 (d, *J* = 5.2 Hz, 1H), 7.80 – 7.78 (m, 1H), 7.72 – 7.68 (m, 3H), 7.53–7.51 (m, 4H), 7.31 – 7.26 (m, 3H, overlapping with $CHCl_3$), 7.14–7.11 (m, 3H), 6.88 (dd, *J* = 15.6, 3.1 Hz, 1H).

^{13}C NMR (126 MHz, $CDCl_3$, ppm) δ 180.7, 154.3, 145.8, 141.6, 137.4, 134.7, 134.6, 133.9, 130.5, 130.2, 129.5, 128.8, 128.6, 128.3, 125.1, 121.8, 118.3. One carbon signal could not be located.

3. Copies of ^{1}H and ^{13}C NMR spectra

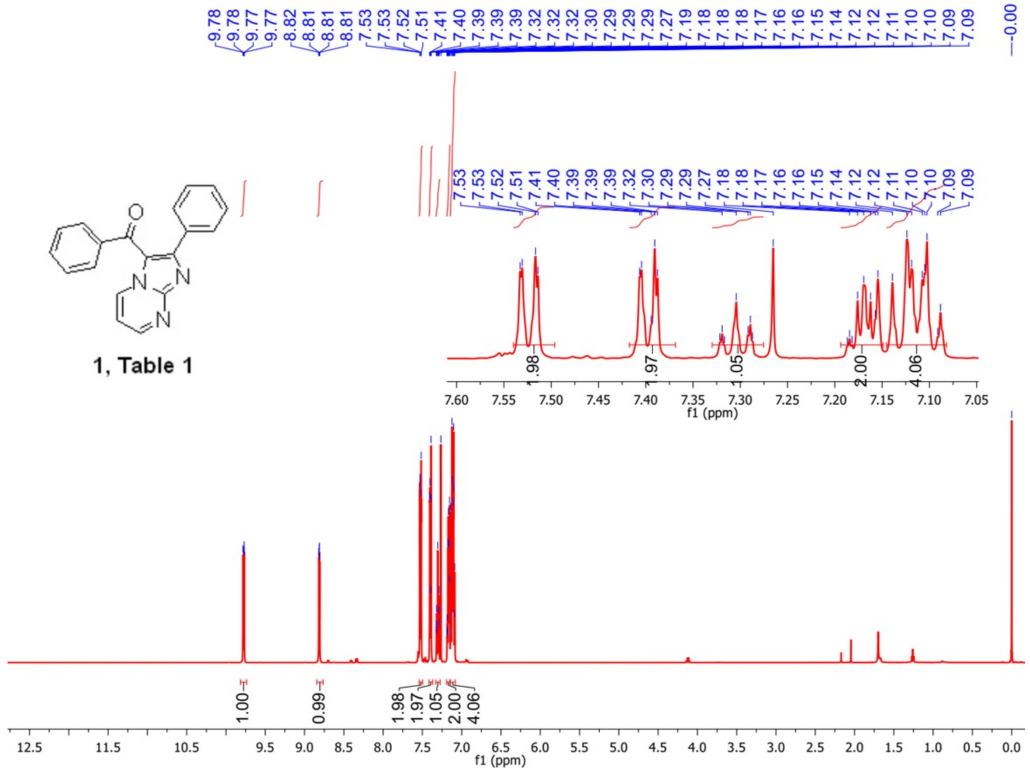

Fig. S1 ^{1}H spectrum of phenyl(2-phenylimidazo[1,2-*a*]pyrimidin-3-yl)methanone.

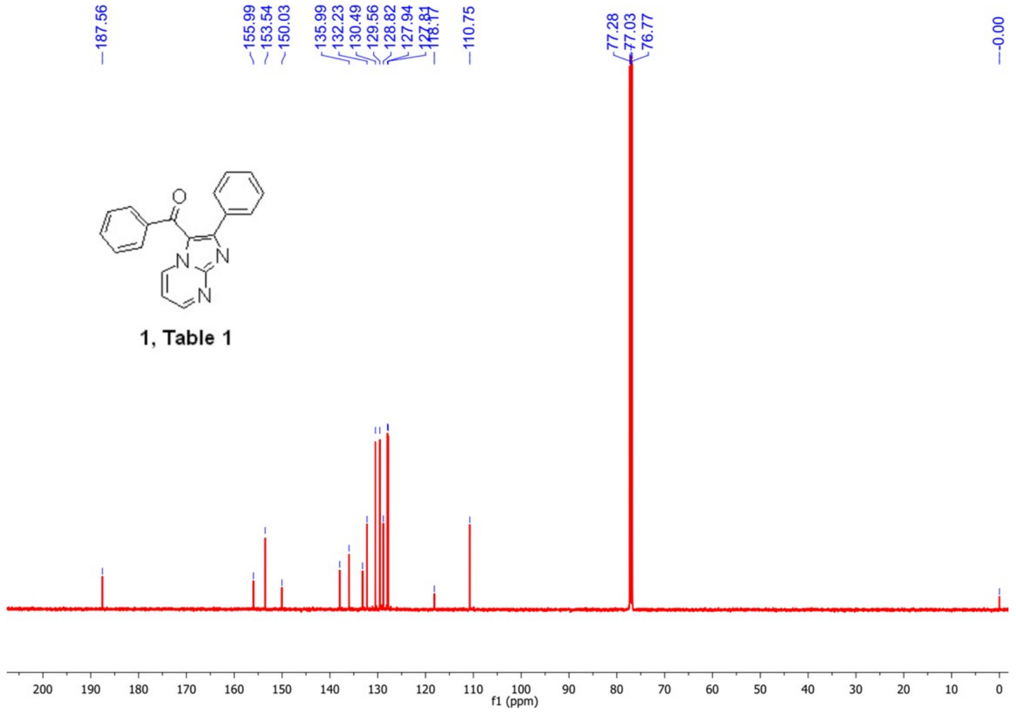

Fig. S2 ^{13}C spectrum of phenyl(2-phenylimidazo[1,2-*a*]pyrimidin-3-yl)methanone.

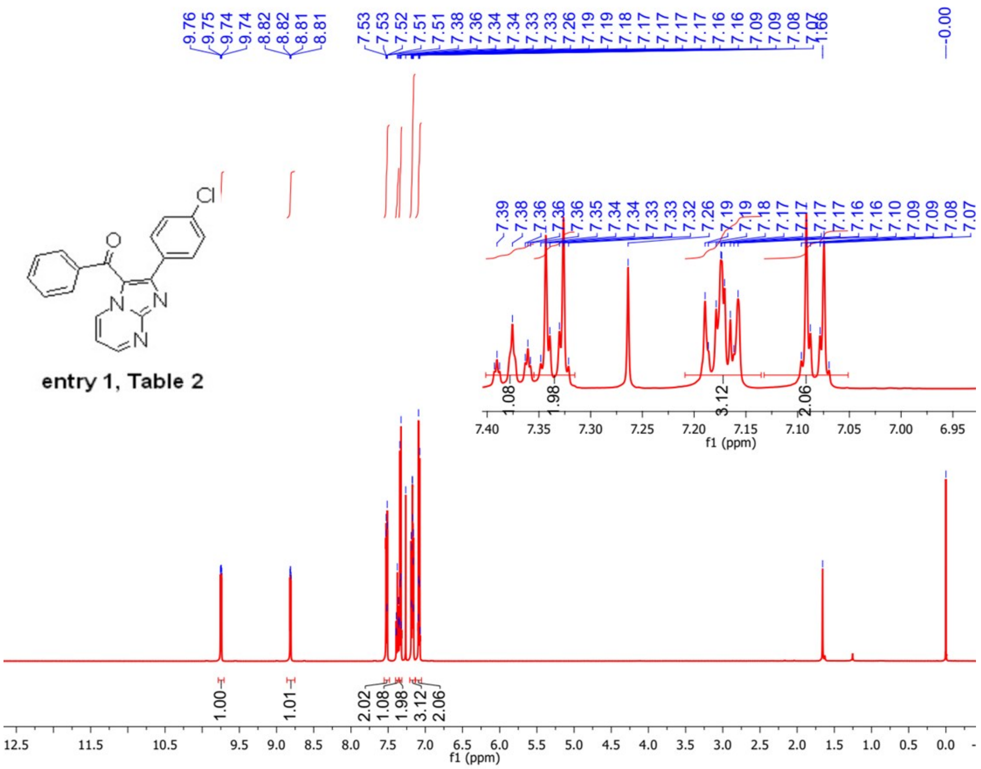

Fig. S3 ^{1}H spectrum of phenyl(2-(4-chlorophenyl)imidazo[1,2-*a*]pyrimidin-3-yl)methanone.

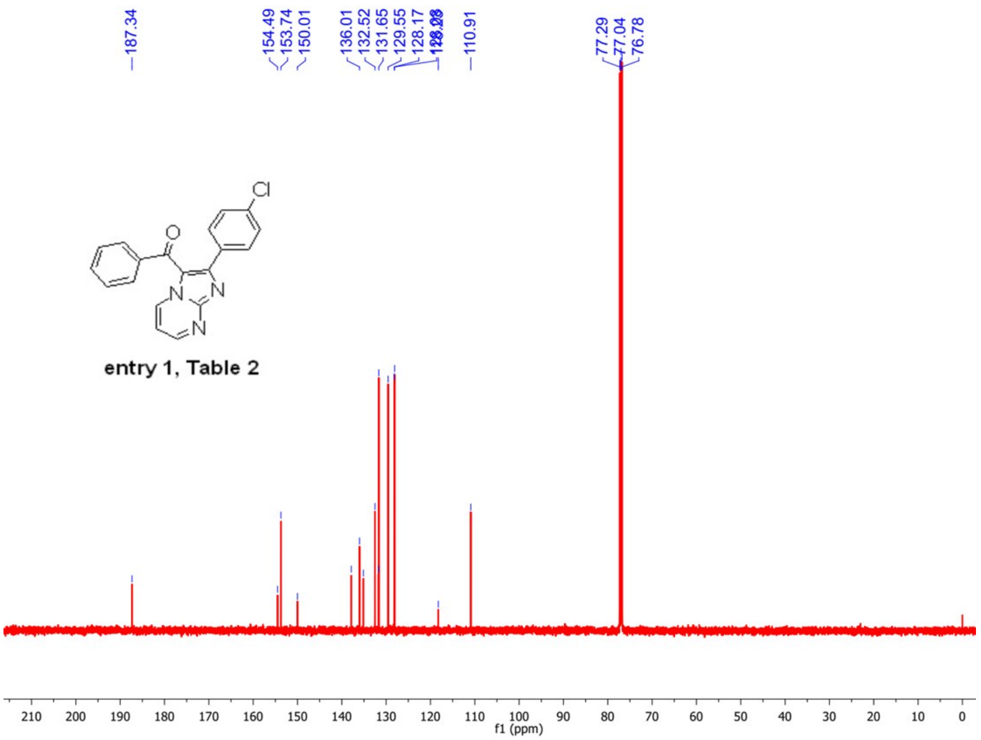

Fig. S4 ^{13}C spectrum of phenyl(2-(4-chlorophenyl)imidazo[1,2-*a*]pyrimidin-3-yl)methanone.

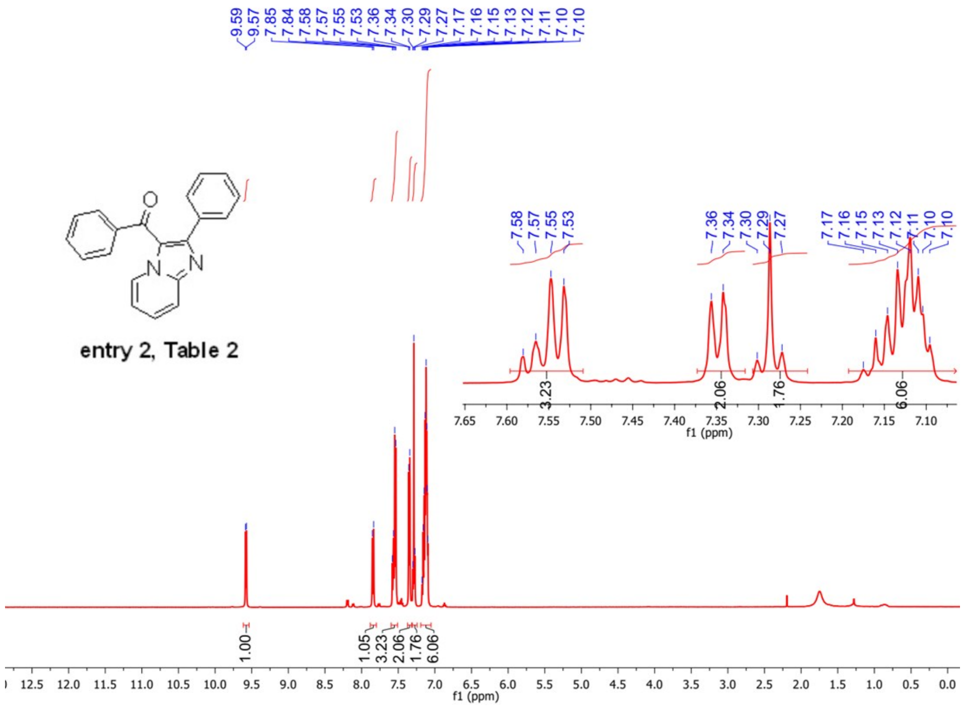

Fig. S5 ^{1}H spectrum of phenyl(2-phenylimidazo[1,2-*a*]pyridin-3-yl)methanone.

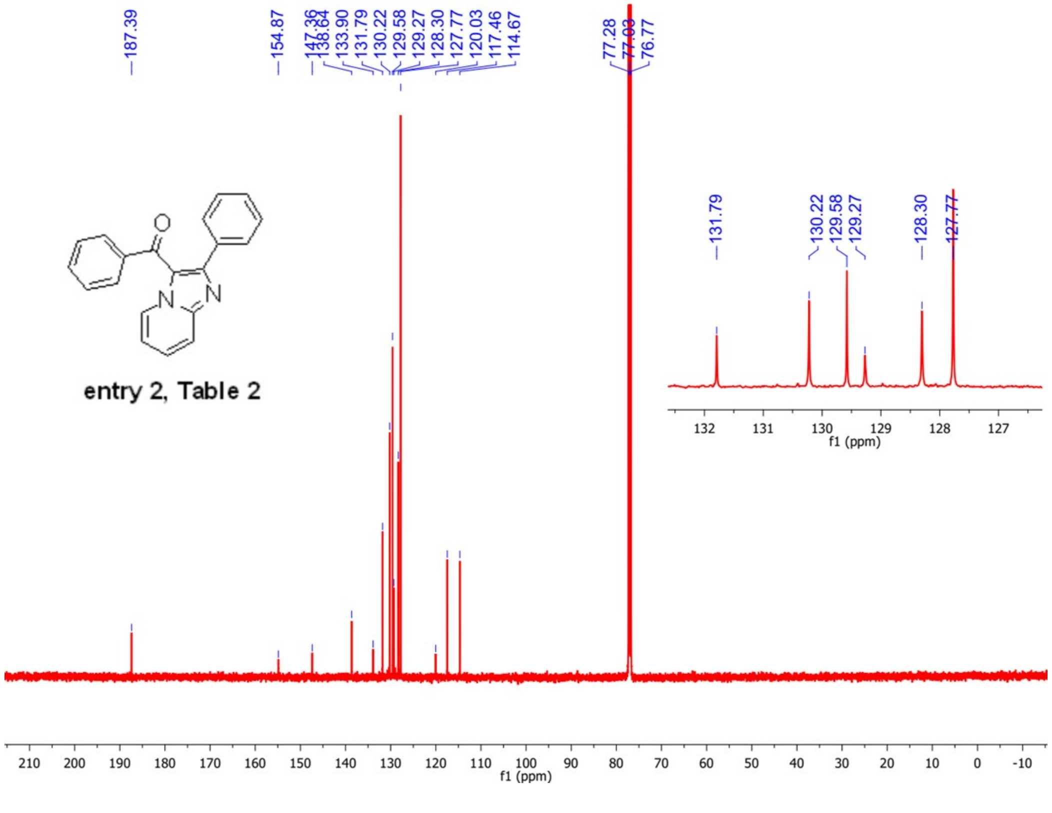

Fig. S6 ^{13}C spectrum of phenyl(2-phenylimidazo[1,2-*a*]pyridin-3-yl)methanone.

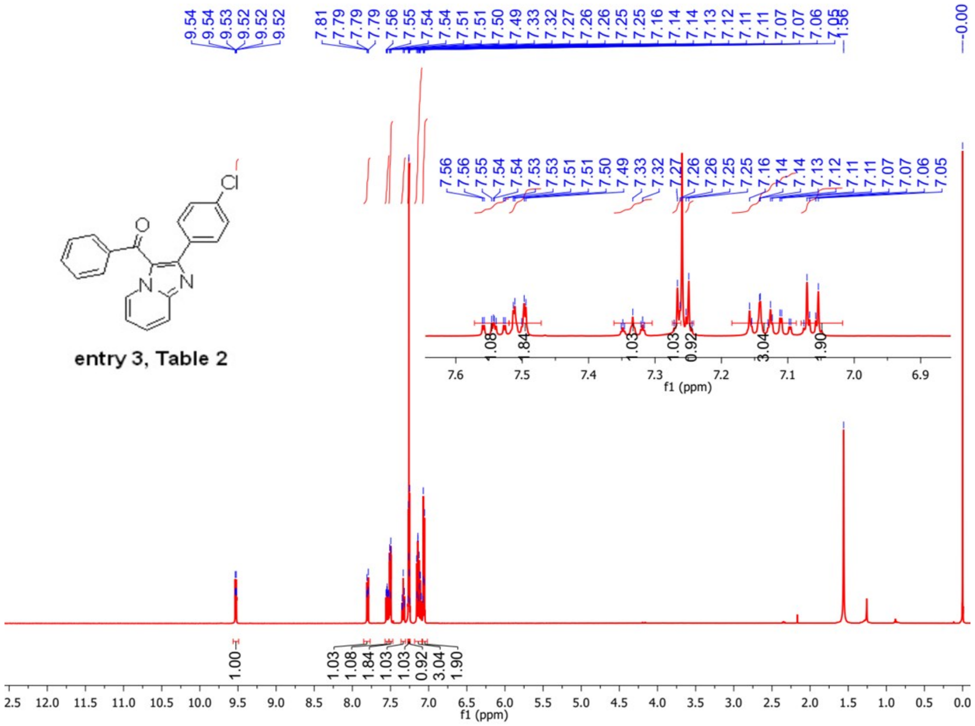

Fig. S7 ^{1}H spectrum of (2-(4-chlorophenyl)imidazo[1,2-*a*]pyridin-3-yl)(phenyl)methanone.

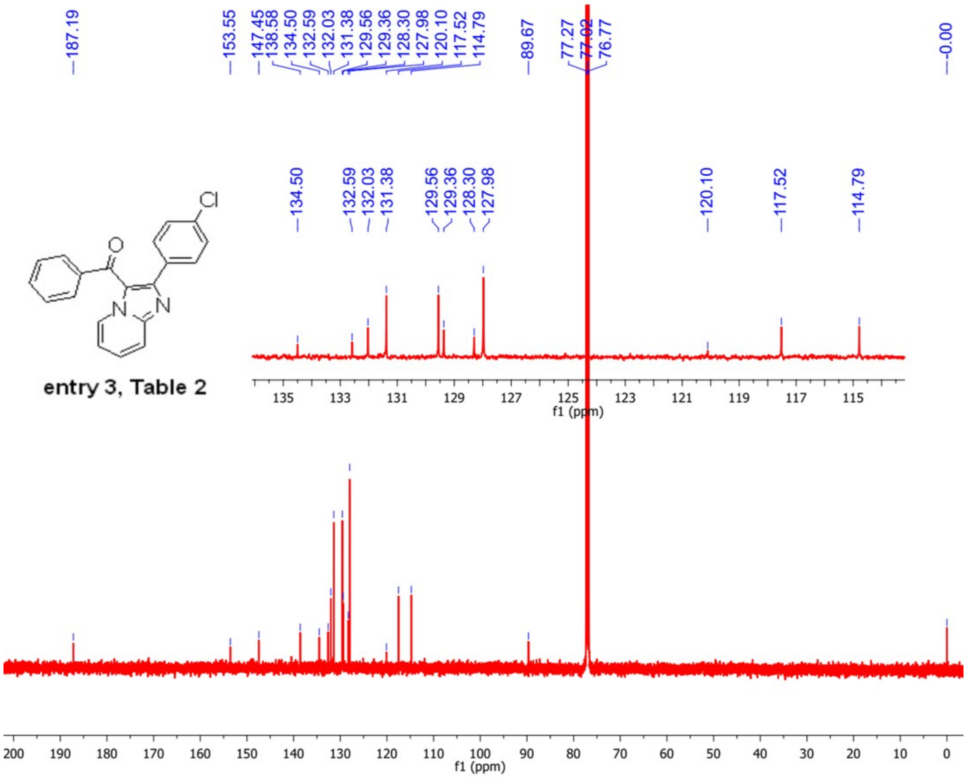

Fig. S8 ^{13}C spectrum of (2-(4-chlorophenyl)imidazo[1,2-*a*]pyridin-3-yl)(phenyl)methanone.

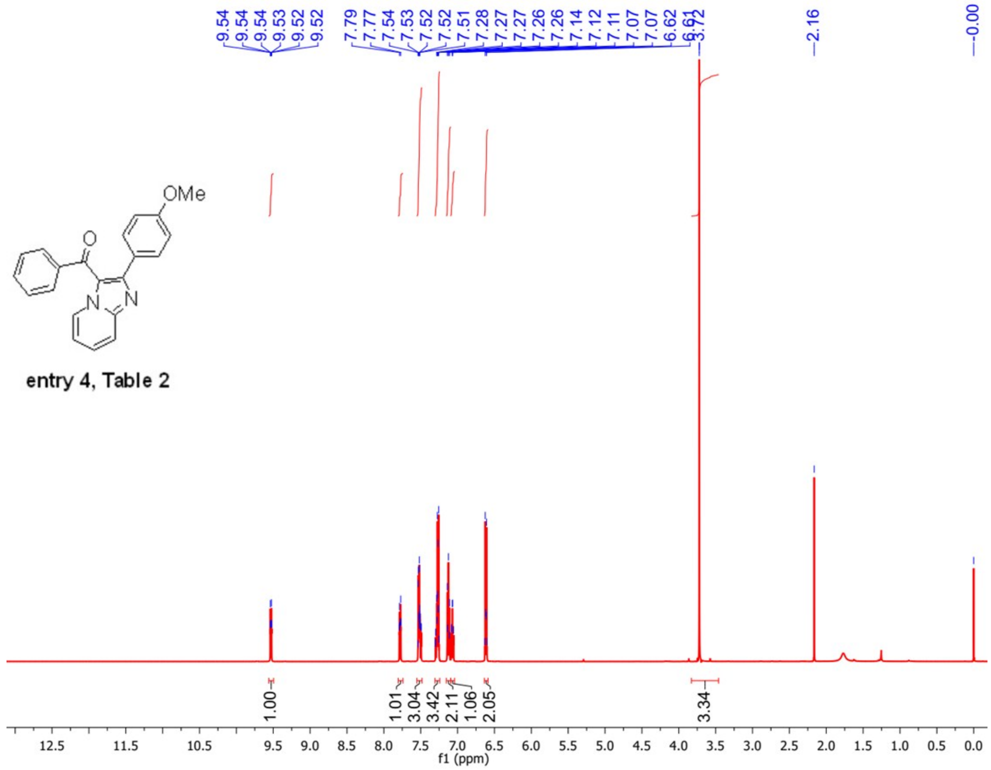

Fig. S9 ^{1}H spectrum of (2-(4-methoxyphenyl)imidazo[1,2-*a*]pyridin-3-yl)(phenyl)methanone.

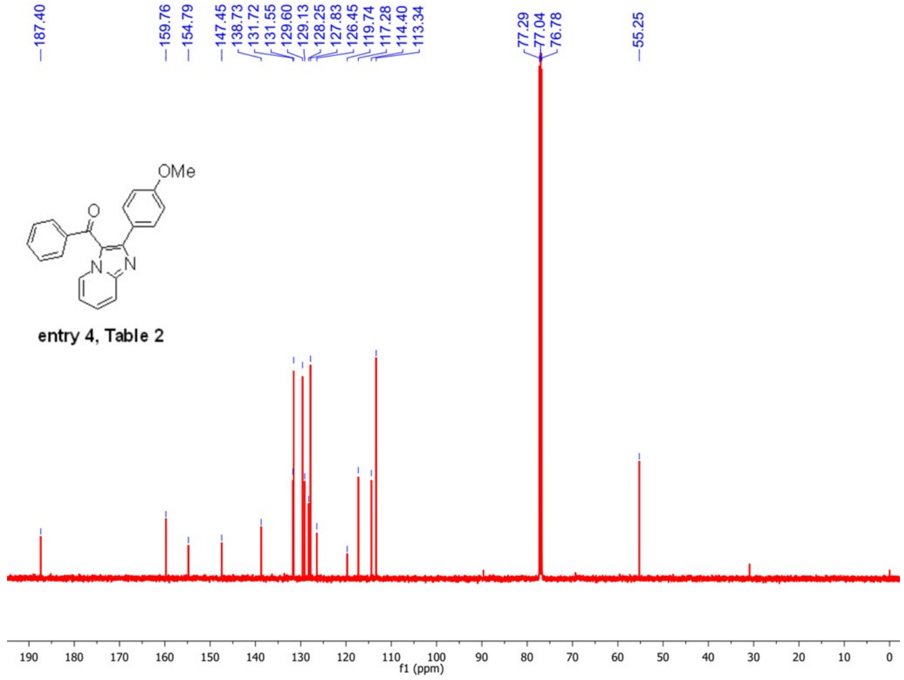

Fig. S10 ^{13}C spectrum of (2-(4-methoxyphenyl)imidazo[1,2-*a*]pyridin-3-yl)(phenyl)methanone.

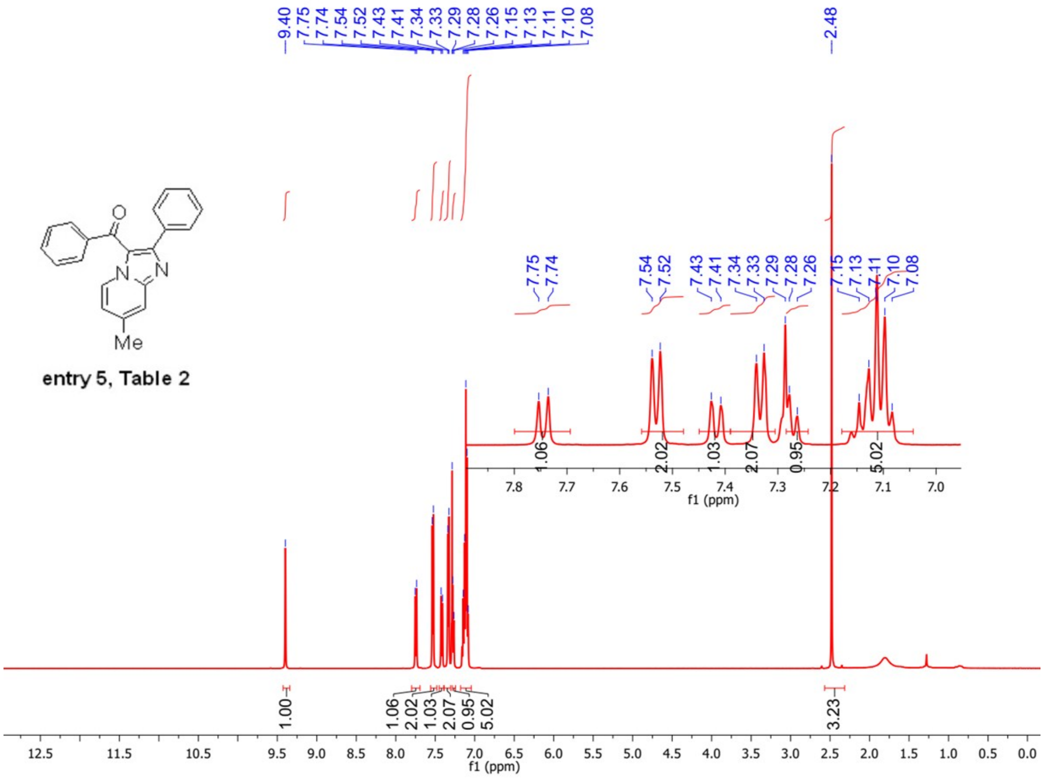

Fig. S11 ^{1}H spectrum of (7-methyl-2-phenylimidazo[1,2-*a*]pyridin-3-yl)(phenyl)methanone.

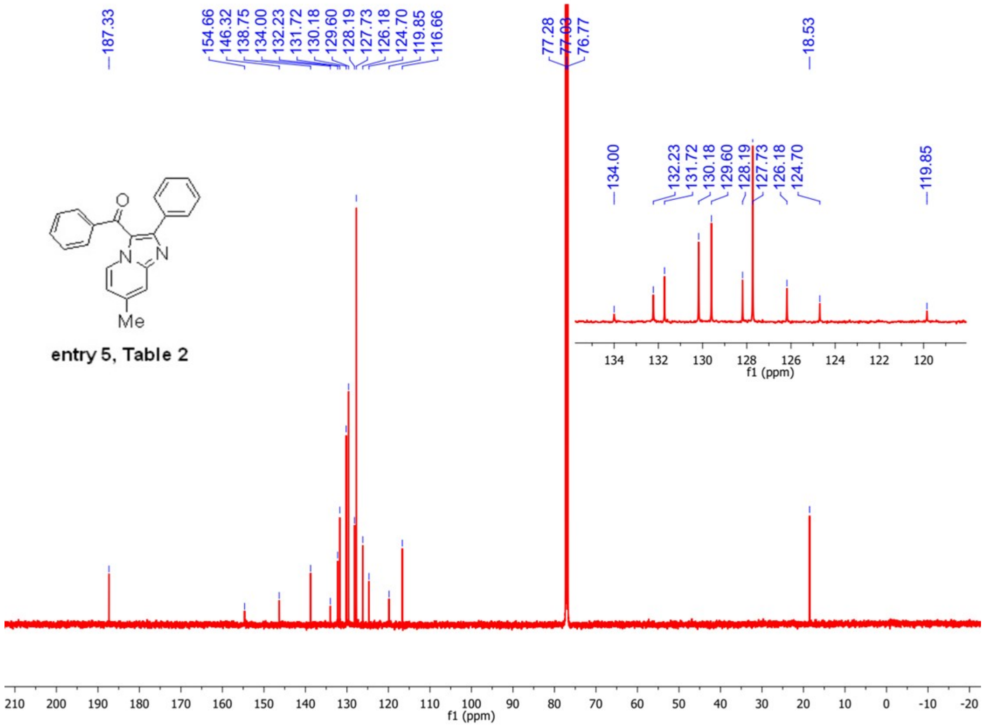

Fig. S12 ^{13}C spectrum of (7-methyl-2-phenylimidazo[1,2-*a*]pyridin-3-yl)(phenyl)methanone.

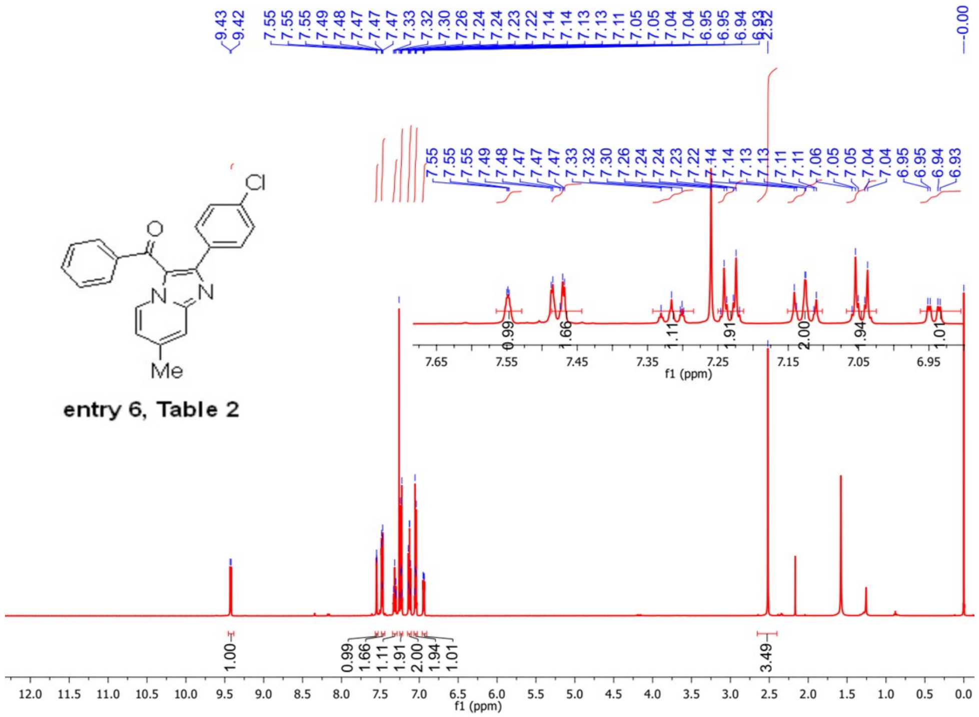

Fig. S13 ^{1}H spectrum of (7-methyl-2-(4-chlorophenyl)imidazo[1,2-*a*]pyridin-3-yl)(phenyl)methanone.

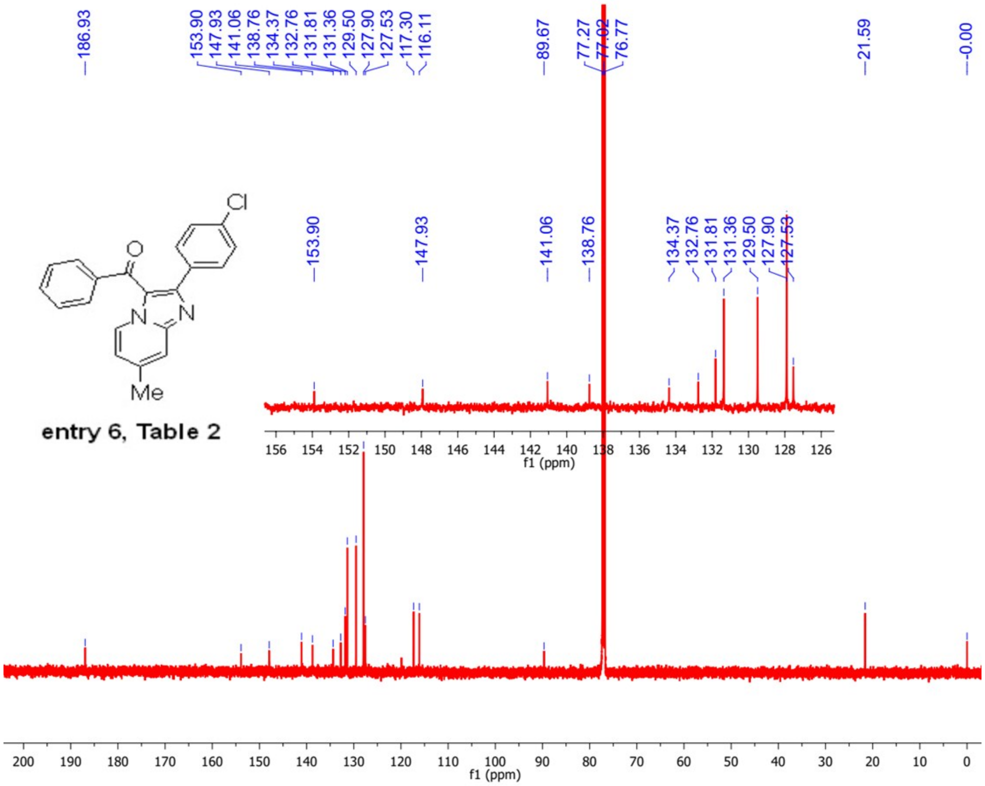

Fig. S14 ^{13}C spectrum of (7-methyl-2-(4-chlorophenyl)imidazo[1,2-*a*]pyridin-3-yl)(phenyl)methanone.

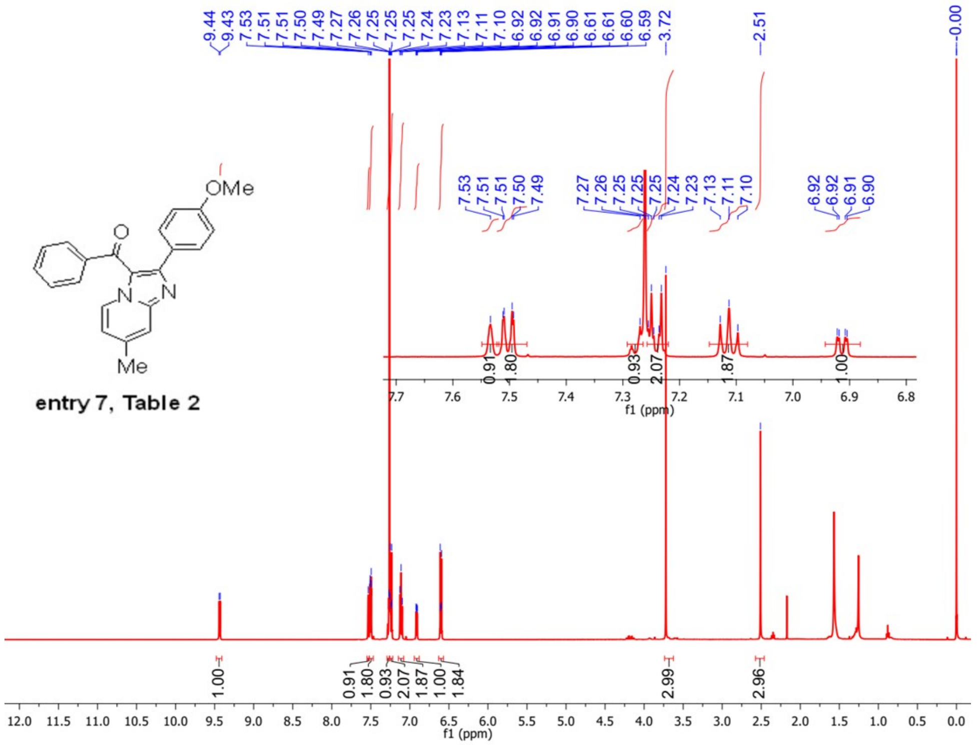

Fig. S15 ^{1}H spectrum of (7-methyl-2-(4-methoxyphenyl)imidazo[1,2-*a*]pyridin-3-yl)(phenyl)methanone.

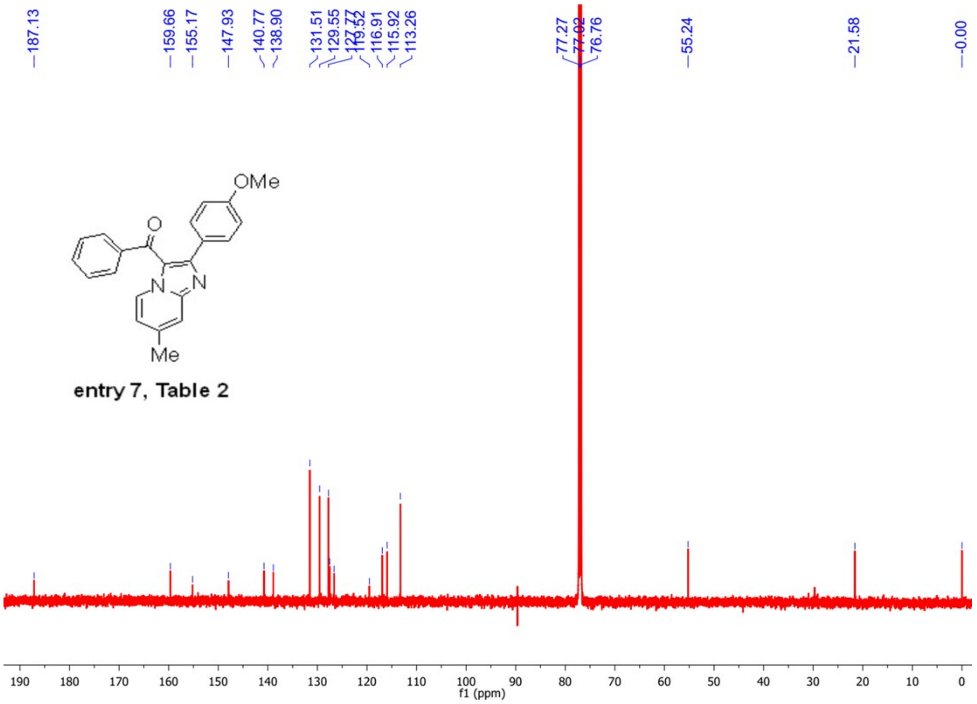

Fig. S16 ^{13}C spectrum of (7-methyl-2-(4-methoxyphenyl)imidazo[1,2-*a*]pyridin-3-yl)(phenyl)methanone.

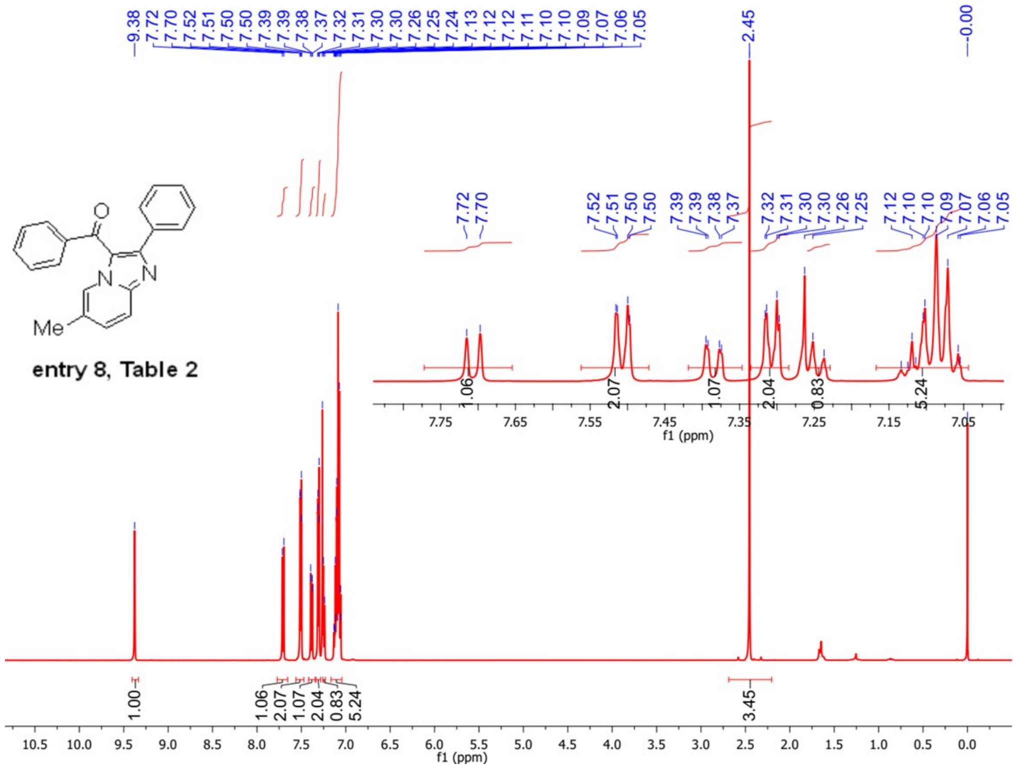

Fig. S17 ^{1}H spectrum of (6-methyl-2-phenyl)imidazo[1,2-*a*]pyridin-3-yl)(phenyl)methanone.

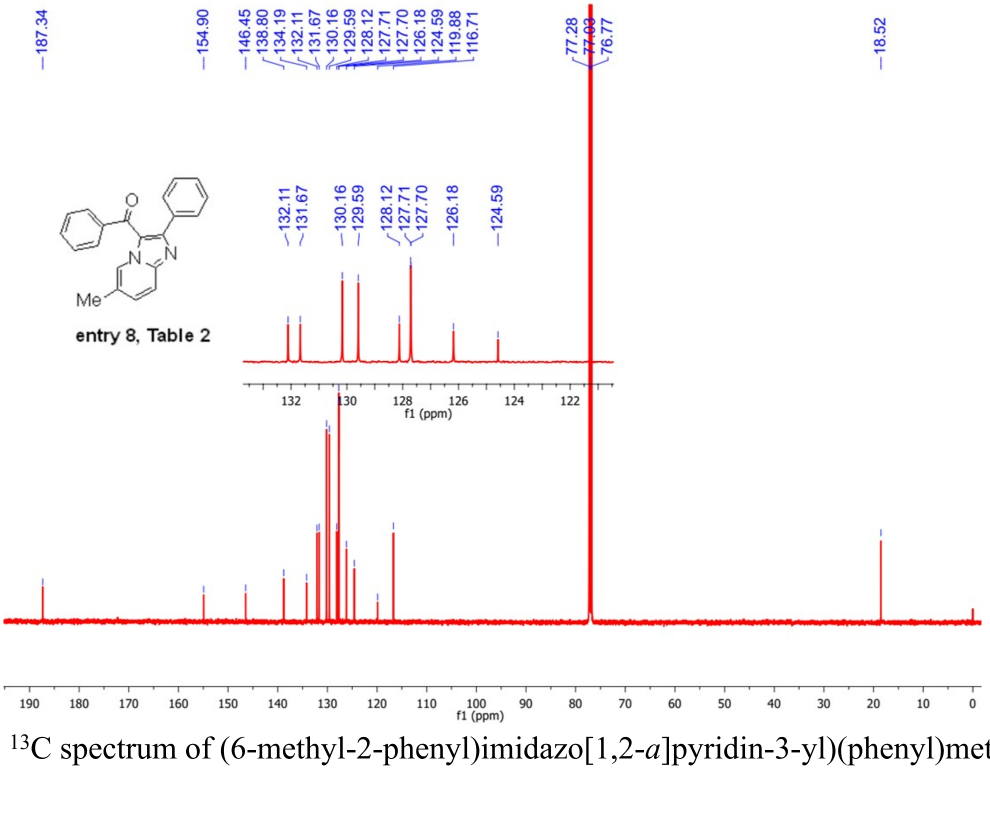

Fig. S18 ^{13}C spectrum of (6-methyl-2-phenyl)imidazo[1,2-*a*]pyridin-3-yl)(phenyl)methanone.

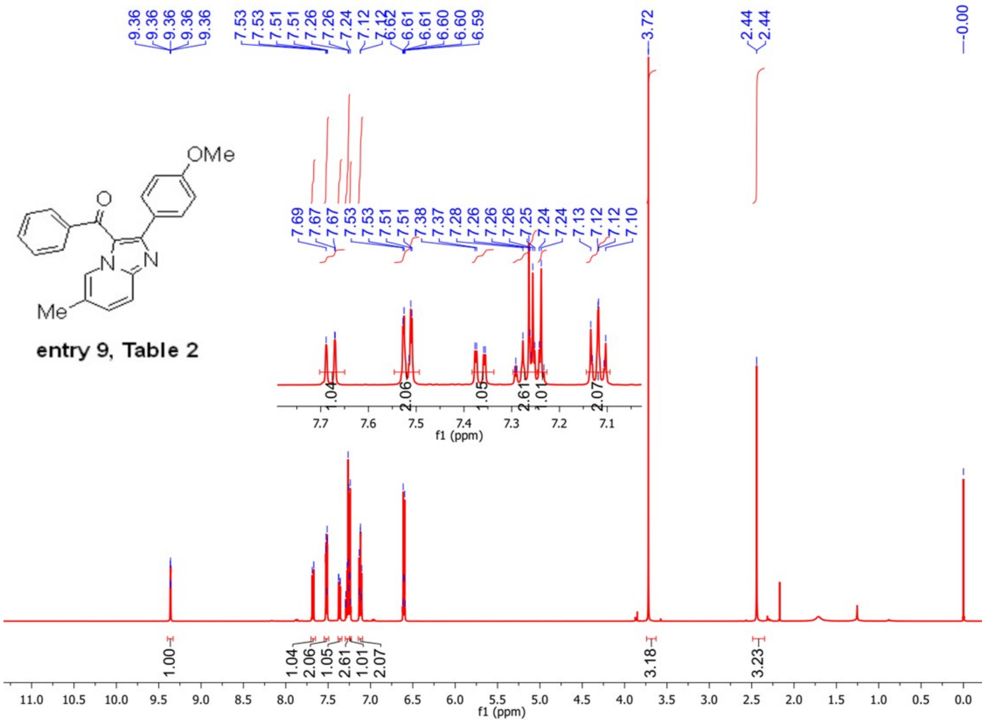

Fig. S19 ^{1}H spectrum of (6-methyl-2-(4-methoxyphenyl)imidazo[1,2-*a*]pyridin-3-yl)(phenyl)methanone.

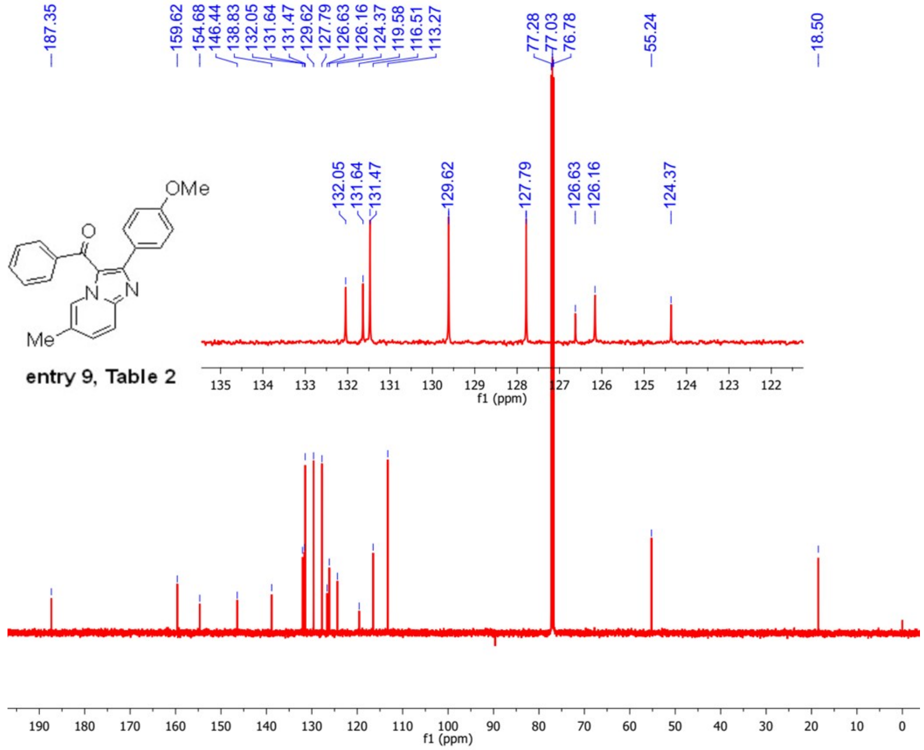

Fig. S20 ^{13}C spectrum of (6-methyl-2-(4-methoxyphenyl)imidazo[1,2-*a*]pyridin-3-yl)(phenyl)methanone.

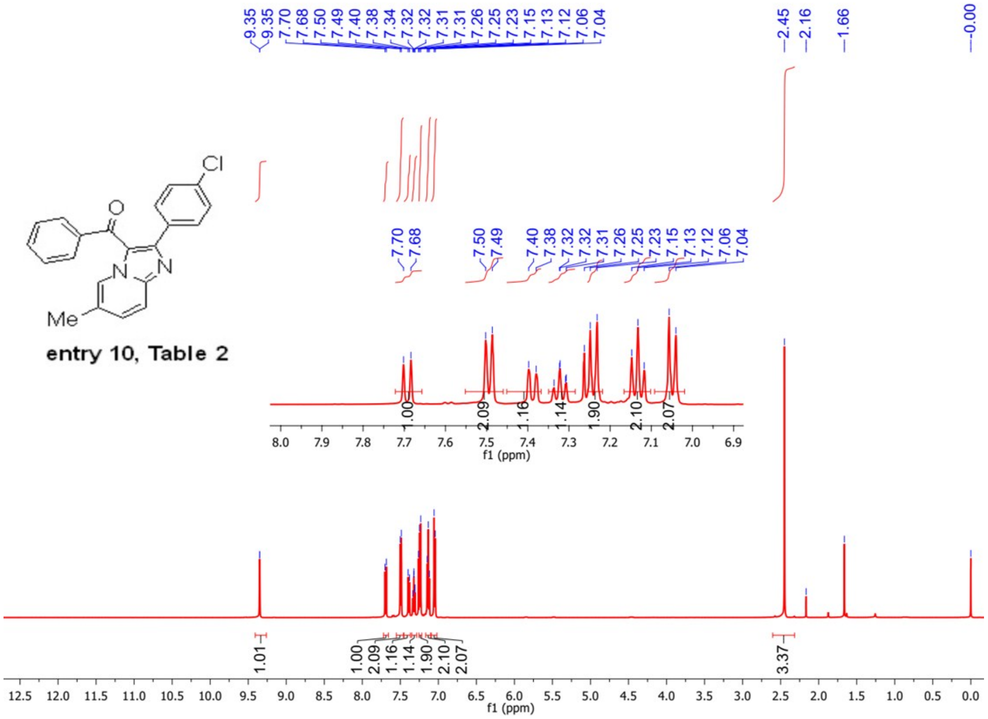

Fig. S21 ^{1}H spectrum of (6-methyl-2-(4-chlorophenyl)imidazo[1,2-*a*]pyridin-3-yl)(phenyl)methanone.

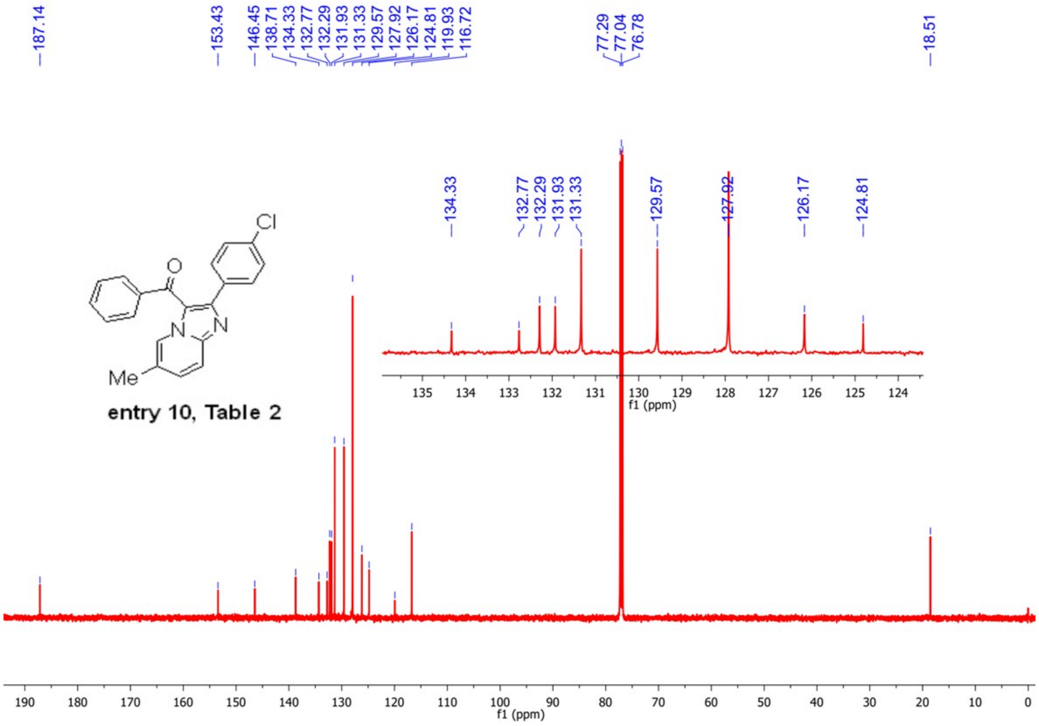

Fig. S22 ^{13}C spectrum of (6-methyl-2-(4-chlorophenyl)imidazo[1,2-*a*]pyridin-3-yl)(phenyl)methanone.

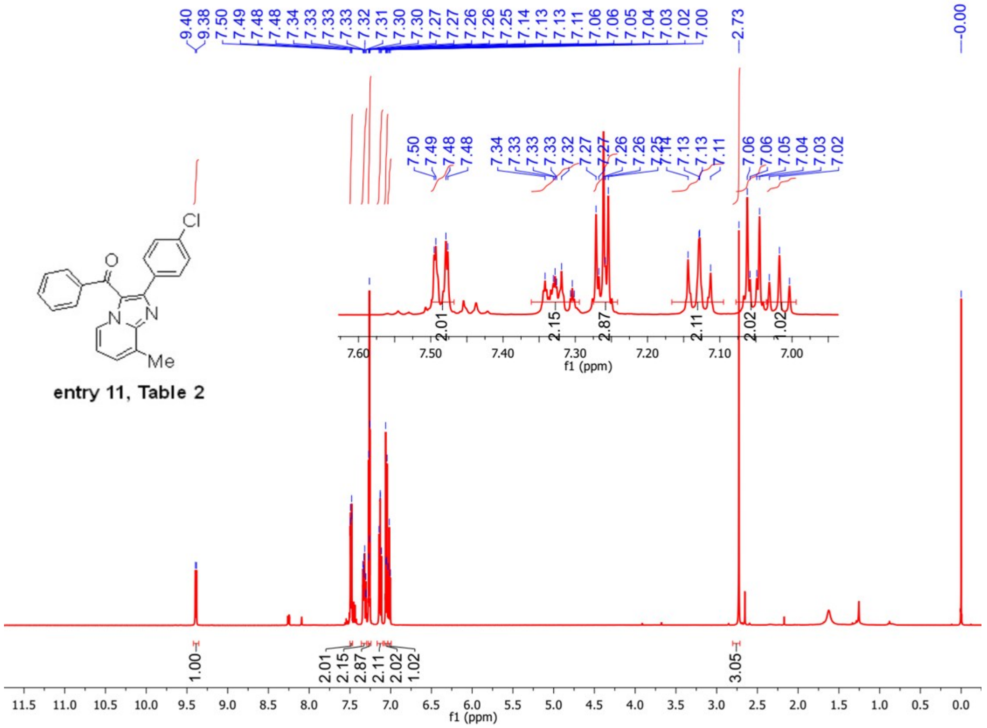

Fig. S23 ^{1}H spectrum of (2-(4-chlorophenyl)-8-methylimidazo[1,2-*a*]pyridin-3-yl)(phenyl)methanone.

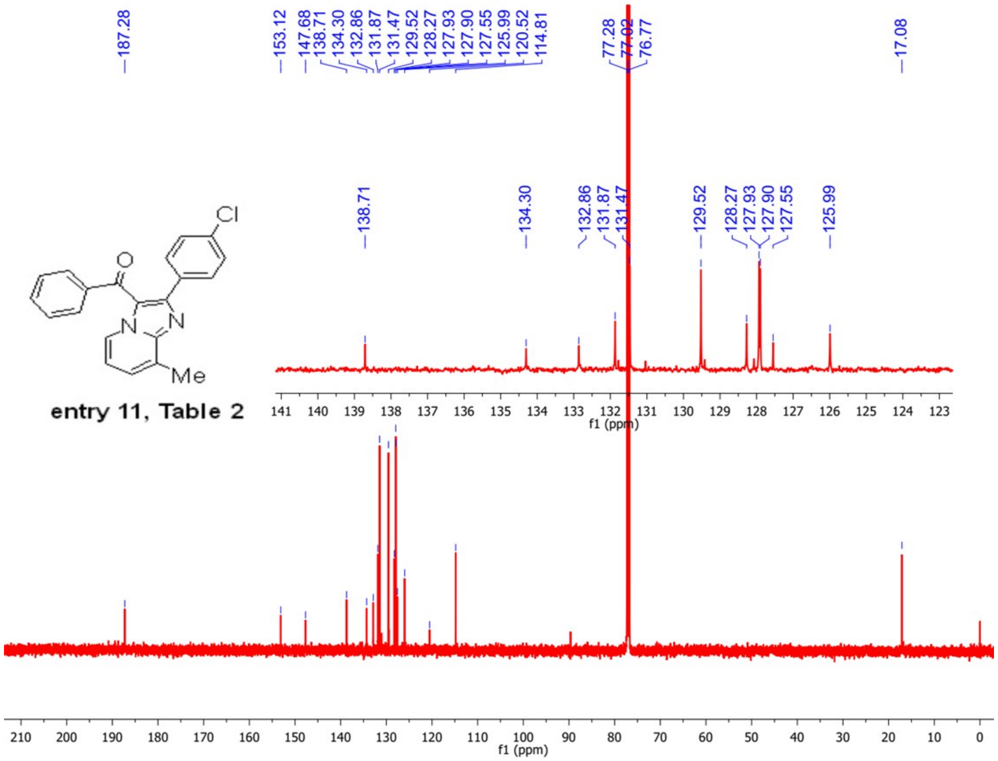

Fig. S24 ^{13}C spectrum of (2-(4-chlorophenyl)-8-methylimidazo[1,2-*a*]pyridin-3-yl)(phenyl)methanone.

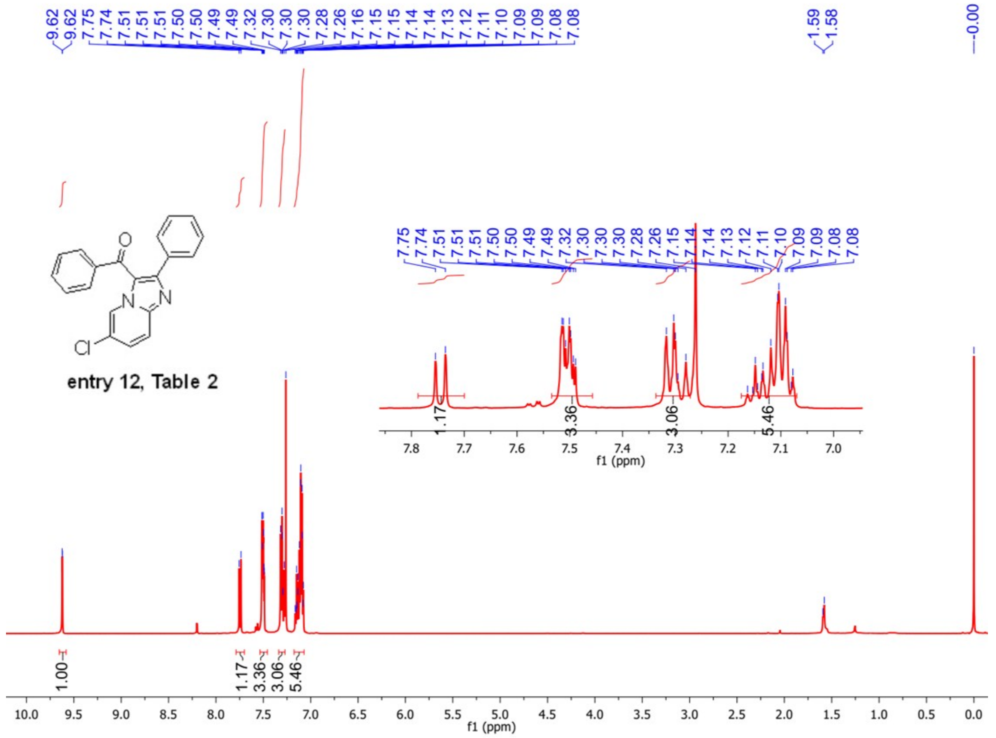

Fig. S25 ^{1}H spectrum of (6-chloro-2-phenylimidazo[1,2-*a*]pyridin-3-yl)(phenyl)methanone.

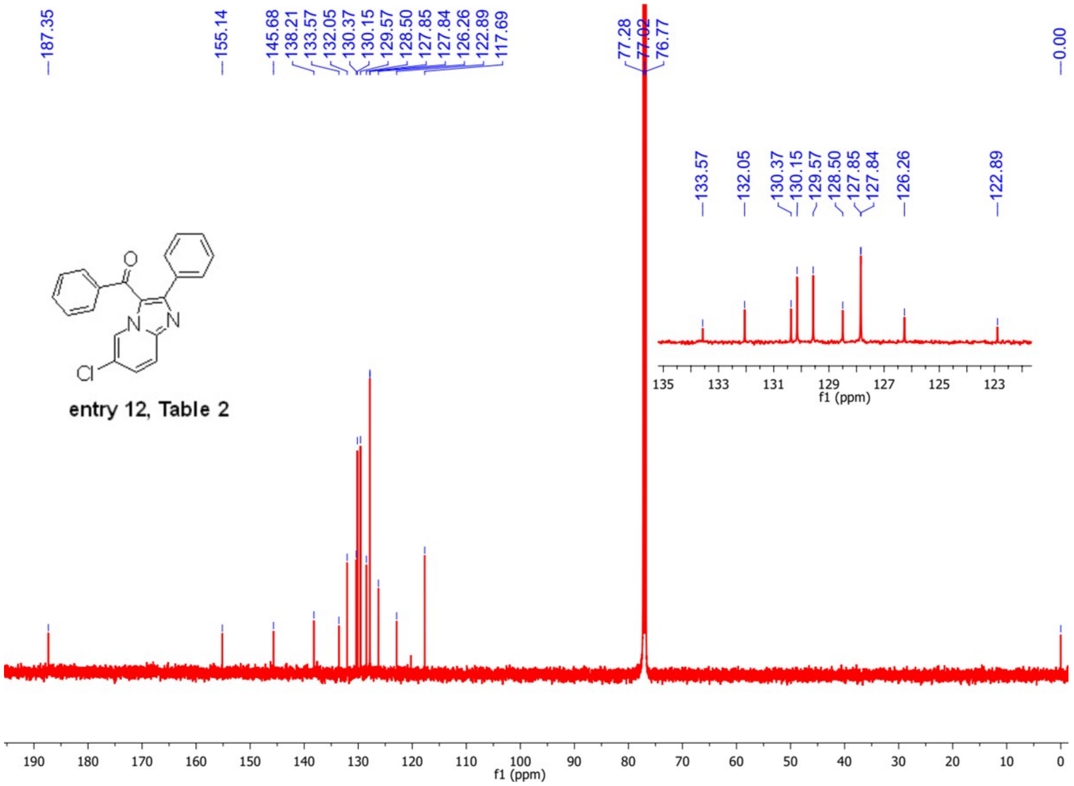

Fig. S26 ^{13}C spectrum of (6-chloro-2-phenylimidazo[1,2-*a*]pyridin-3-yl)(phenyl)methanone.

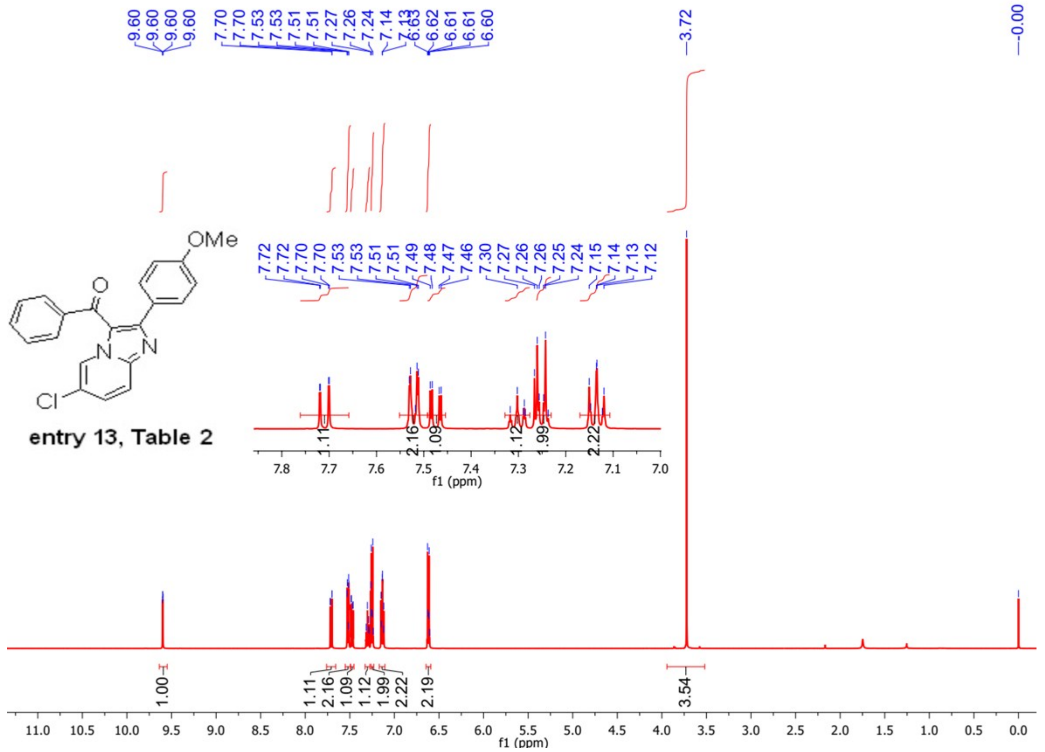

Fig. S27 ^{1}H spectrum of (6-chloro-2-(4-methoxyphenyl)imidazo[1,2-*a*]pyridin-3-yl)(phenyl)methanone.

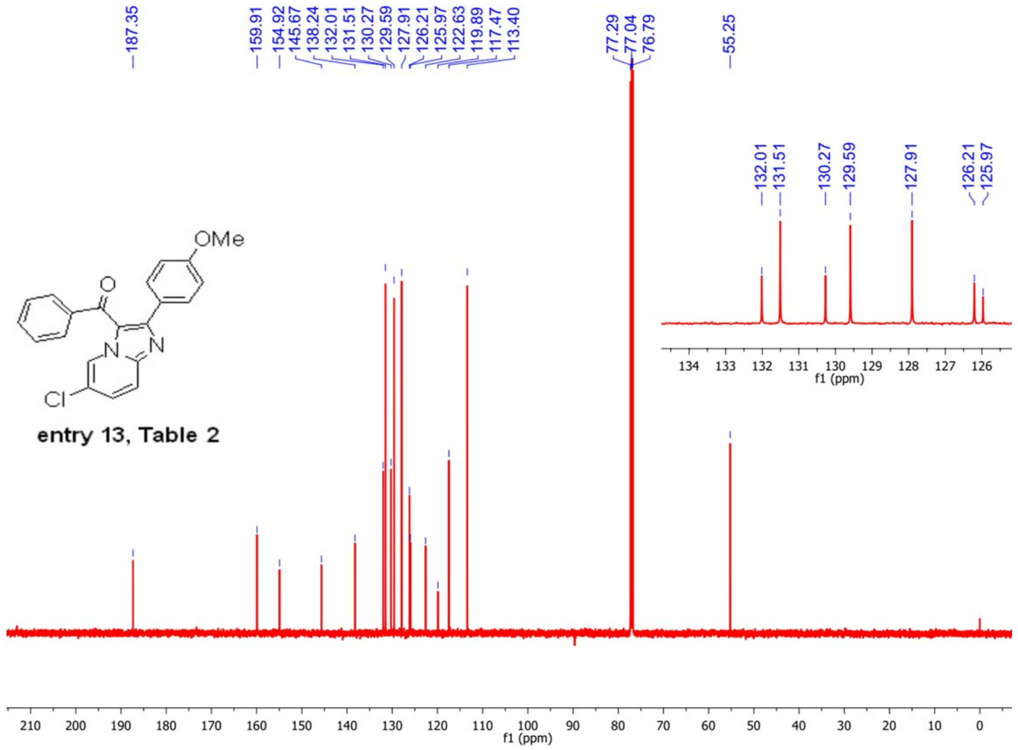

Fig. S28 ^{13}C spectrum of (6-chloro-2-(4-methoxyphenyl)imidazo[1,2-*a*]pyridin-3-yl)(phenyl)methanone.

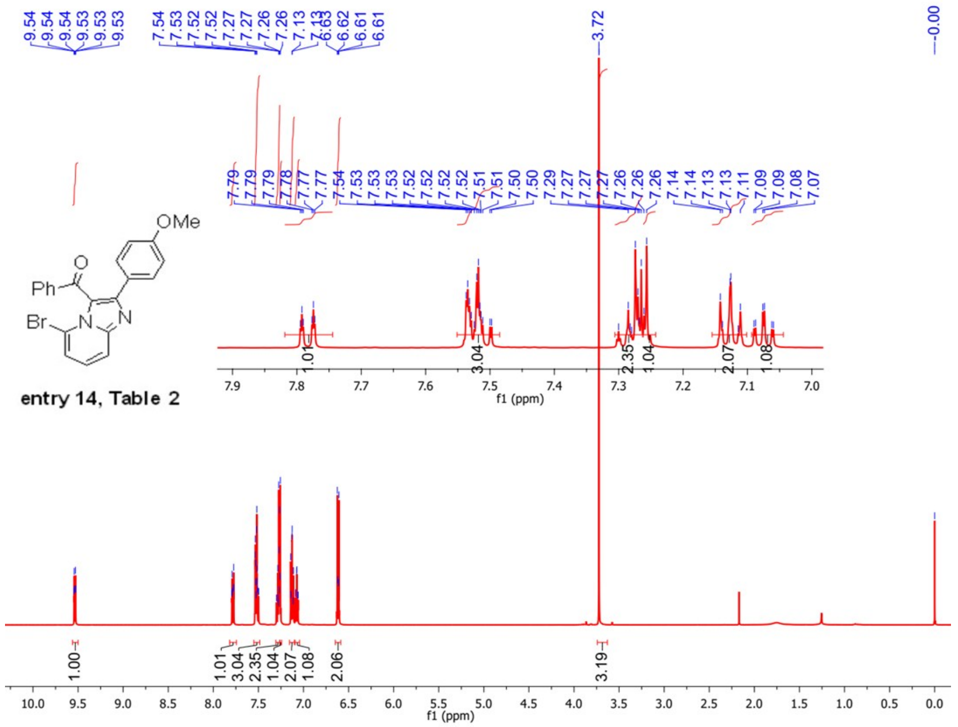

Fig. S29 ^{1}H spectrum of (5-bromo-2-(4-methoxyphenyl)imidazo[1,2-*a*]pyridin-3-yl)(phenyl)methanone.

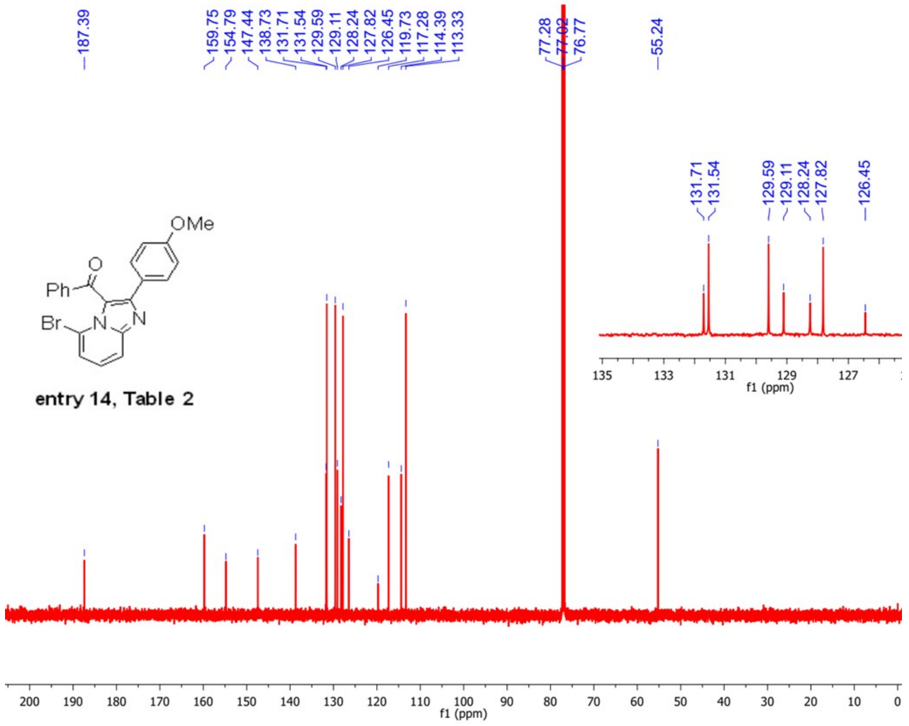

Fig. S30 ^{13}C spectrum of (5-bromo-2-(4-methoxyphenyl)imidazo[1,2-*a*]pyridin-3-yl)(phenyl)methanone.

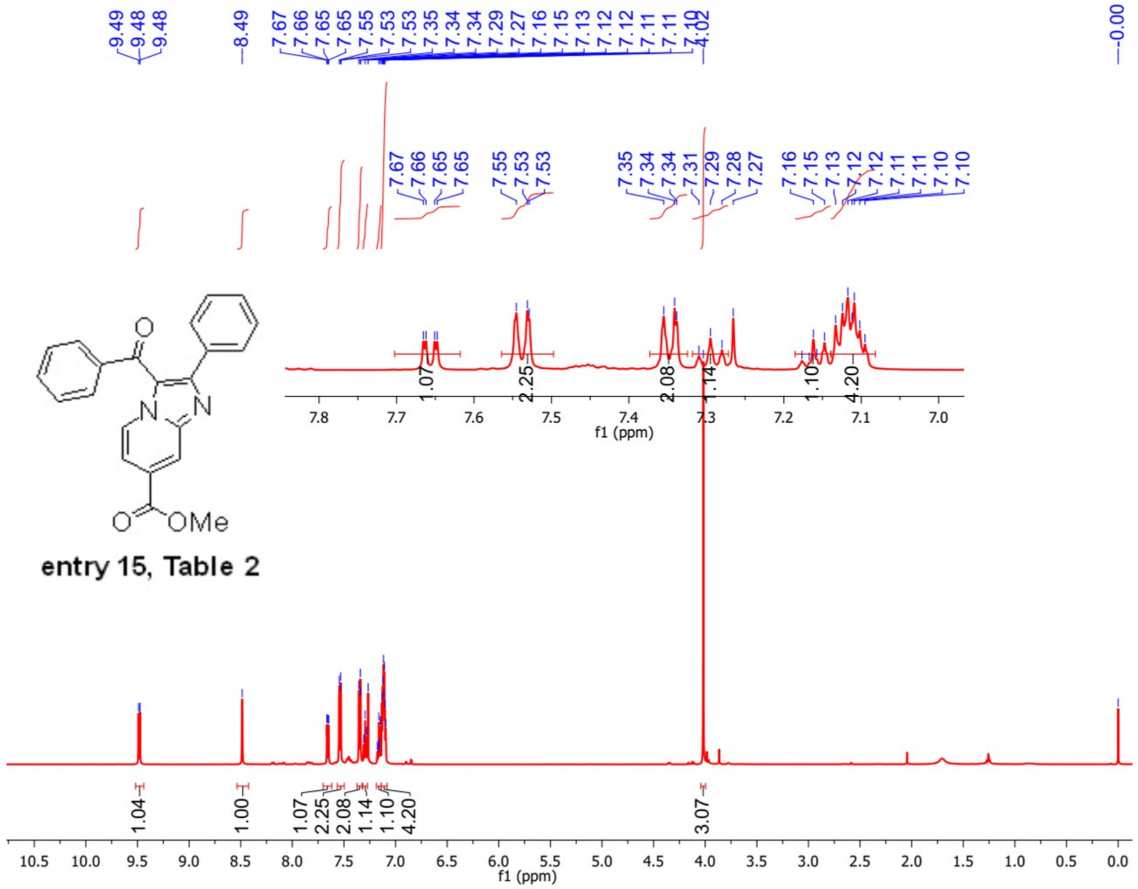

Fig. S31 ^{1}H spectrum of methyl 3-benzoyl-2-phenylimidazo[1,2-*a*]pyridine-6-carboxylate.

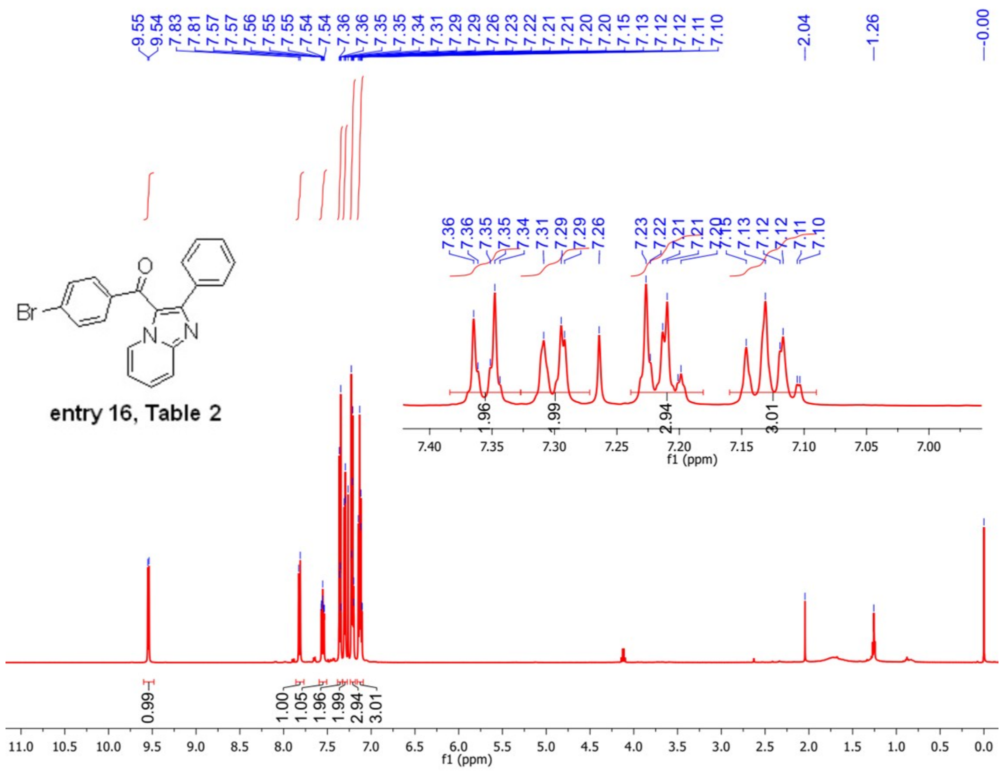

Fig. S32 ^{1}H spectrum of (4-bromophenyl)(2-phenylimidazo[1,2-*a*]pyridin-3-yl)methanone.

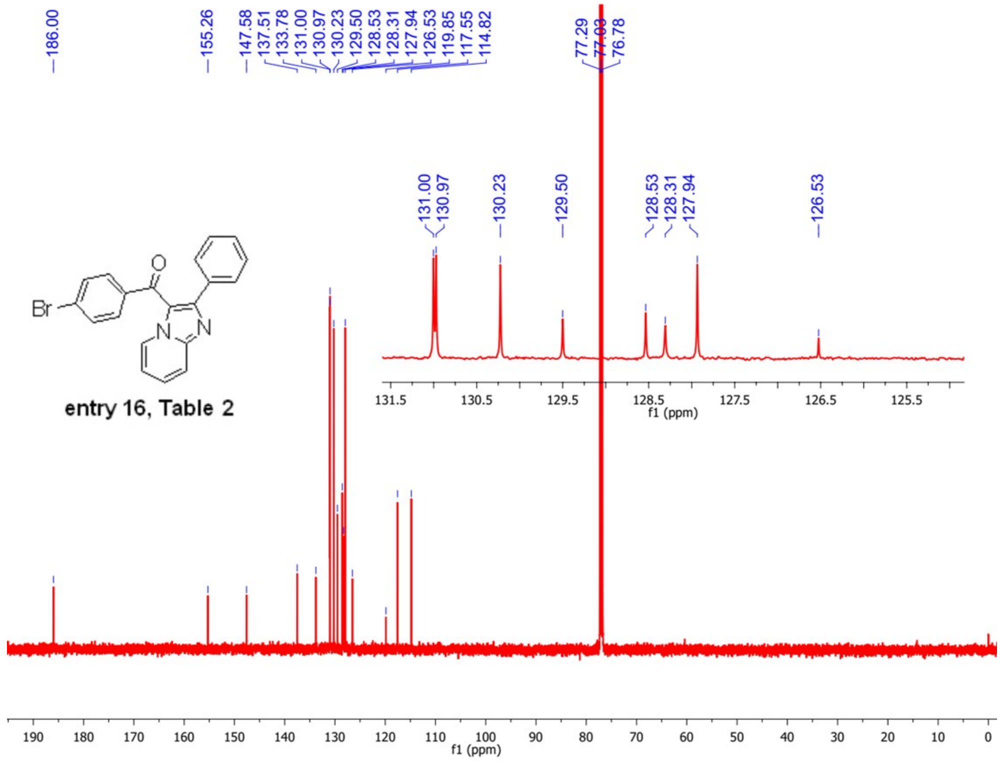

Fig. S33 ^{13}C spectrum of (4-bromophenyl)(2-phenylimidazo[1,2-*a*]pyridin-3-yl)methanone.

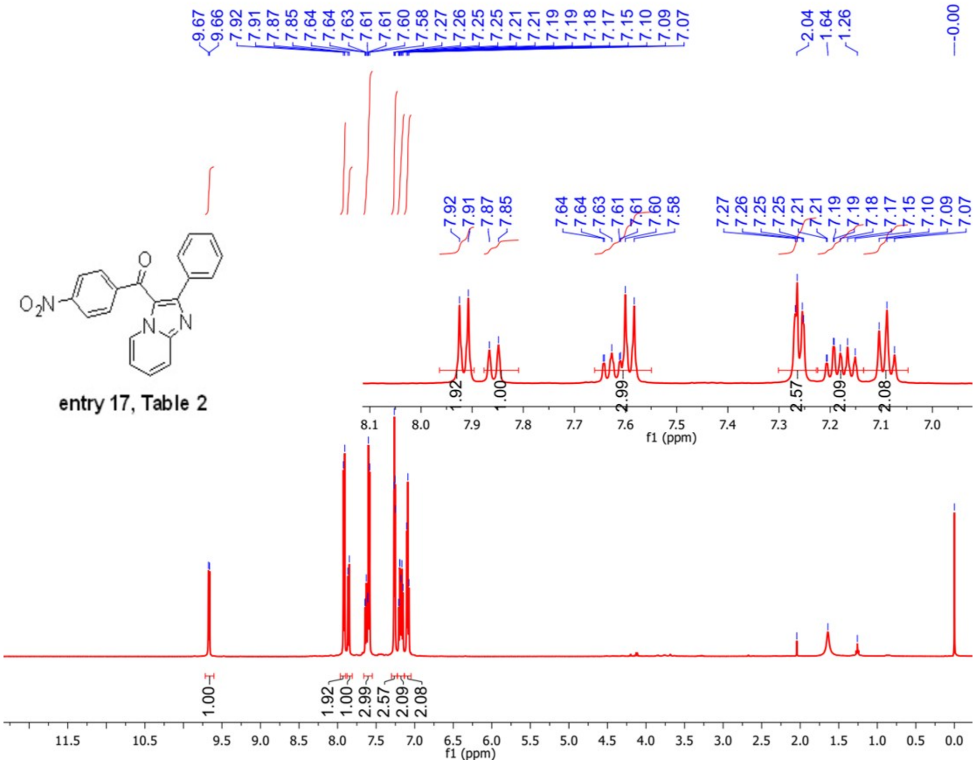

Fig. S34 ^{1}H spectrum of (4-nitrophenyl)(2-phenylimidazo[1,2-*a*]pyridin-3-yl)methanone.

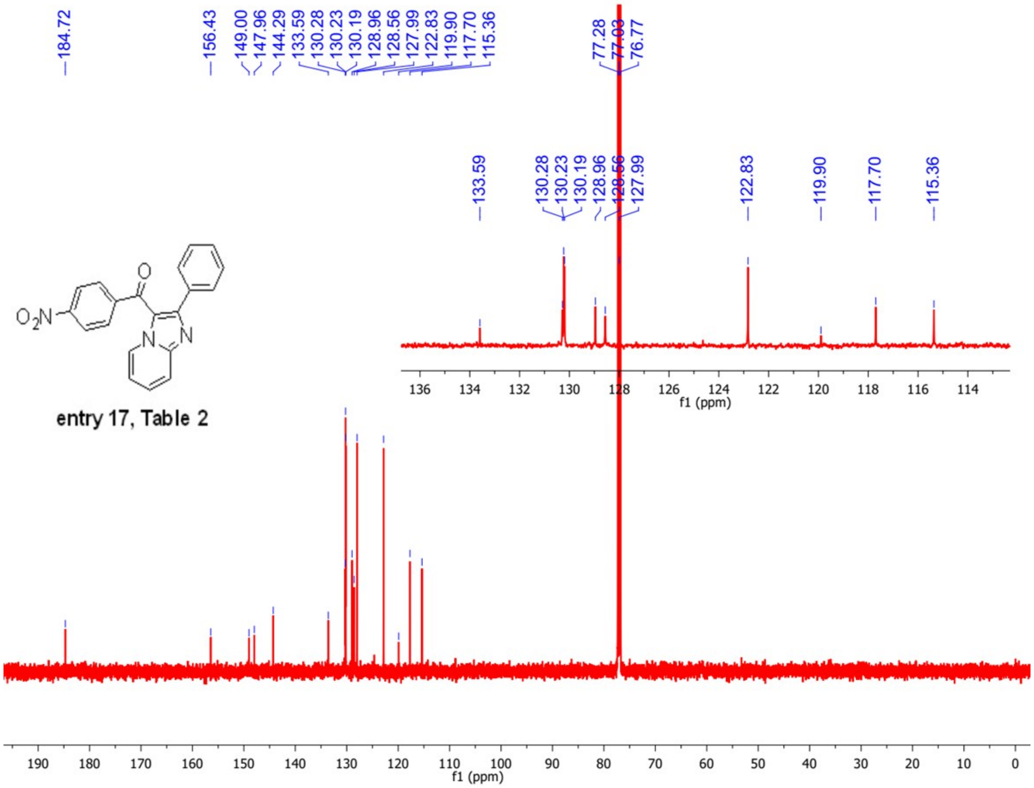

Fig. S35 ^{13}C spectrum of (4-nitrophenyl)(2-phenylimidazo[1,2-*a*]pyridin-3-yl)methanone.

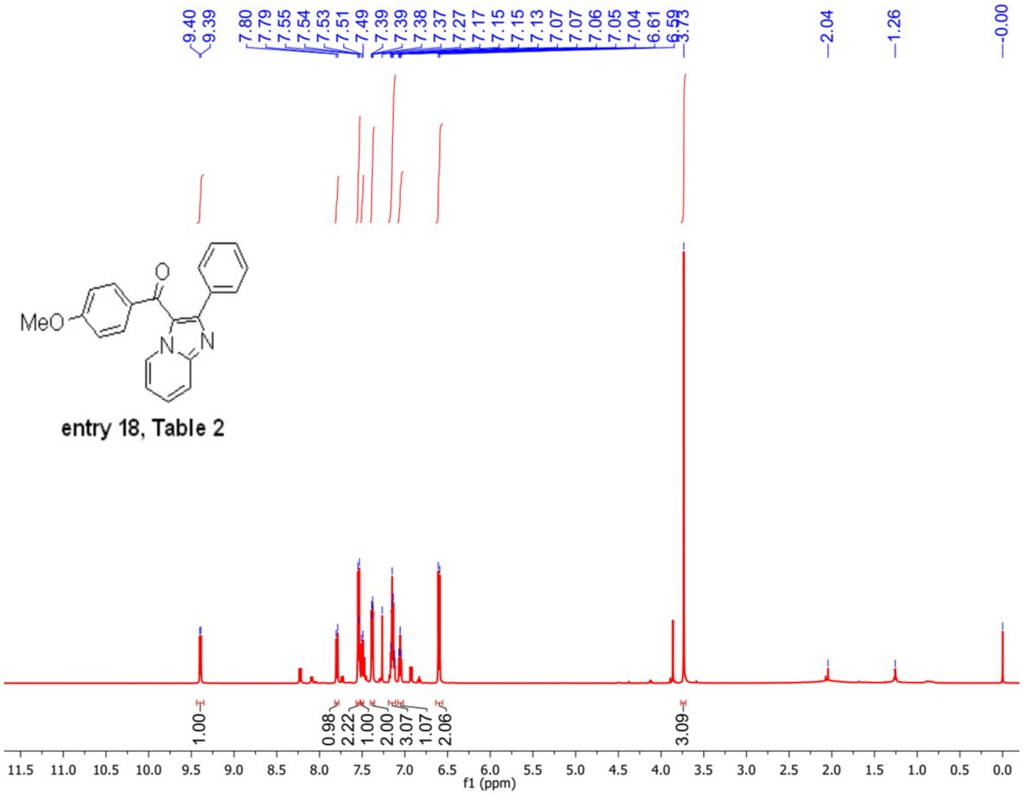

Fig. S36 ^{1}H spectrum of (4-methoxyphenyl)(2-phenylimidazo[1,2-*a*]pyridin-3-yl)methanone.

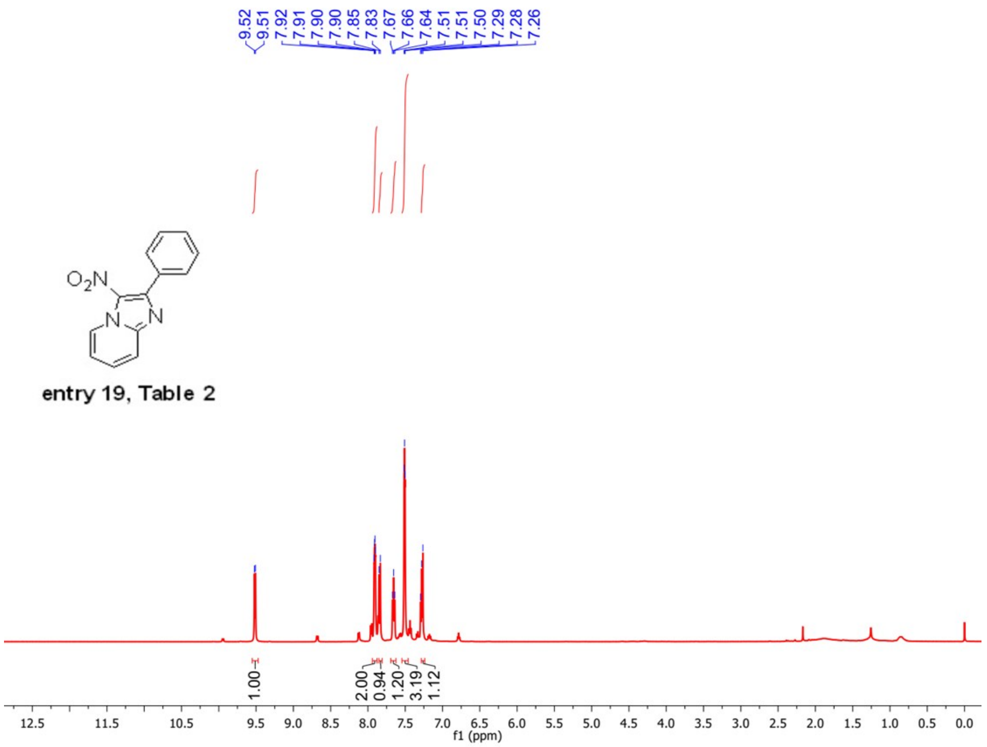

Fig. S37 ^{1}H spectrum of 3-nitro-2-phenylimidazo[1,2-*a*]pyridine.

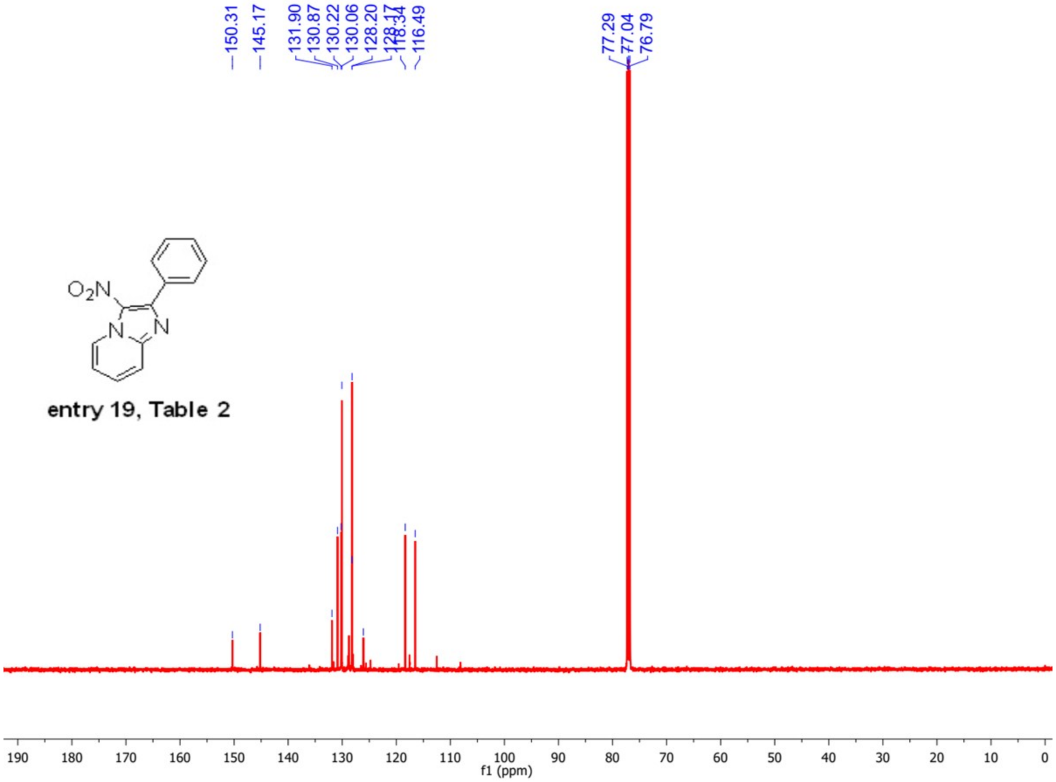

Fig. S38 ^{13}C spectrum of 3-nitro-2-phenylimidazo[1,2-*a*]pyridine.

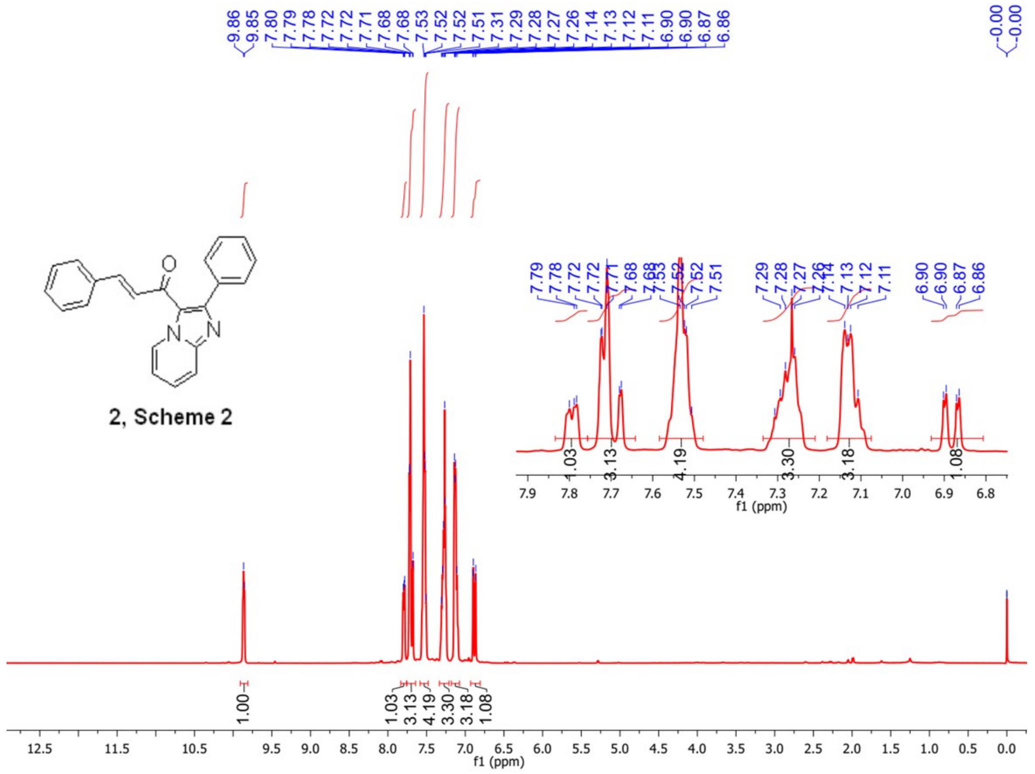

Fig. S39 ^{1}H spectrum of (*E*)-3-phenyl-1-(2-phenylimidazo[1,2-*a*]pyridin-3-yl)prop-2-en-1-one.

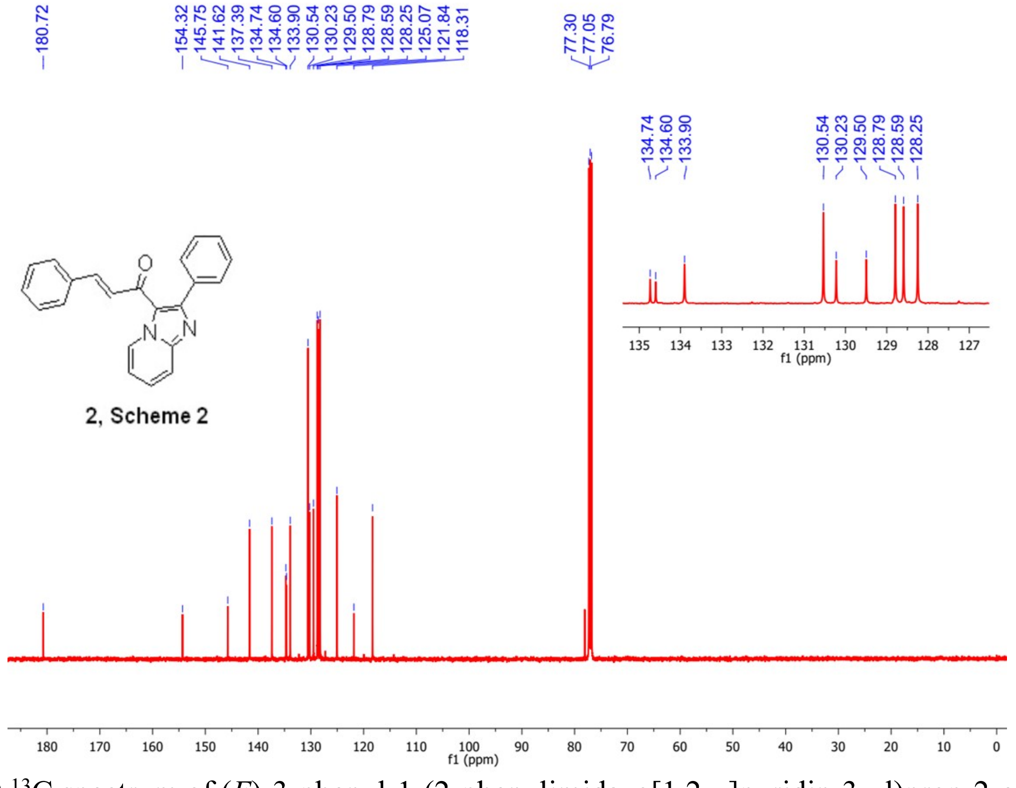

Fig. S40 ^{13}C spectrum of (*E*)-3-phenyl-1-(2-phenylimidazo[1,2-*a*]pyridin-3-yl)prop-2-en-1-one.
